# Supplementary material for: Nucleotide Analogues Bearing a C2′ or C3′-Stereogenic All-Carbon Quaternary Center as SARS-CoV-2 RdRp Inhibitors
Source: Molecules. 2022 Jan 17;27(2):564. doi: 10.3390/molecules27020564 (PMC8781509; doi:10.3390/molecules27020564)

Supporting Information

Nucleotide Analogues bearing a C2′ or C3′-Stereogenic All-Carbon Quaternary Center as SARS-CoV-2 RdRp Inhibitors

Amarender Manchoju[1], Renaud Zelli[1], Gang Wang[1], Carla Eymard[1], Adrian Oo[2], Mona Nemer[3]*, Michel Prévost[1]*, Baek Kim[2,4]*, and Yvan Guindon [1,3,5]*

[1]Bio-Organic Chemistry Laboratory, Institut de Recherches Cliniques de Montréal, Montréal, Québec H2W 1R7, Canada.
[2]Department of Pediatrics, School of Medicine, Emory University, Atlanta, Georgia, USA.
[3]Department of Biochemistry, Microbiology and Immunology, University of Ottawa, Ottawa, ON, K1N 6N5, Canada.
[4]Children's Healthcare of Atlanta, Atlanta, Georgia, USA.
[5]Department of Chemistry, Université de Montréal, Montréal, QC H3C 3J7
*Correspondence authors: mnemer@uottawa.ca (Mona Nemer), michel.prevost@ircm.qc.ca (Michel Prévost), baek.kim@emory.edu (Baek Kim), yvan.guindon@ircm.qc.ca (Yvan Guindon).

Stereochemical Proofs:

The relative *β* configurations of protected and deprotected nucleoside analogues **26-28** and **29** were determined by relevant nuclear Overhauser effect (nOe) enhancements (2D NOESY).

In addition, NOESY experiments further confirmed the 1’,2’-trans stereochemistry.

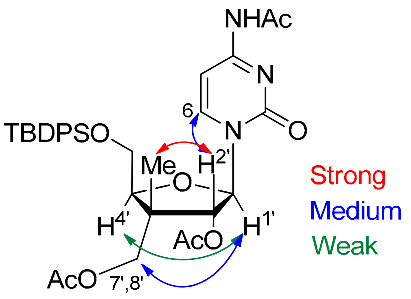

26

NOESY confirmed
β relative stereochemistry

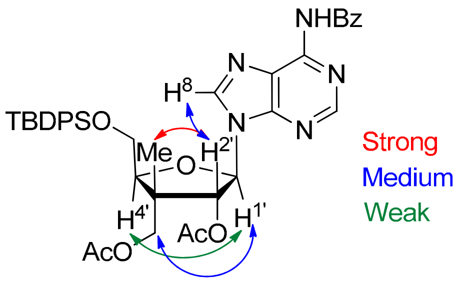

27

NOESY confirmed
β relative stereochemistry

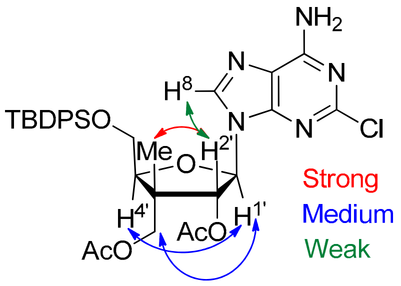

28

NOESY confirmed
β relative stereochemistry

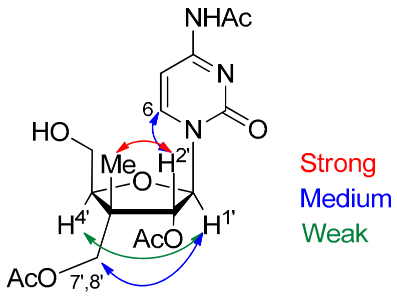

29

NOESY confirmed
β relative stereochemistry

The N^9 regiochemistry of the purine ring of compounds **30-31** and **33-34** was determined from HSQC and HMBC experiments. In addition, NOESY experiments further confirmed the 1’,2’-*trans* stereochemistry.

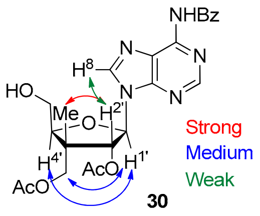

NOESY confirmed
β relative stereochemistry

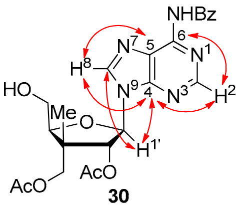

From HSQC

H^{8} (8.82 ppm) ⟷ C^{8} (144.5 ppm)
H^{2} (8.70 ppm) ⟷ C^{2} (153.1 ppm)
H$^{1'}$ (6.35 ppm) ⟷ C$^{1'}$ (88.4 ppm)

From HMBC

H^{8} (8.82 ppm) ⟷ C^{5} (125.2 ppm)
H^{8} (8.82 ppm) ⟷ C^{4} (153.4 ppm)
H^{2} (8.70 ppm) ⟷ C^{6} (151.2 ppm)
H^{2} (8.70 ppm) ⟷ C^{4} (153.4 ppm)
H$^{1'}$ (6.35 ppm) ⟷ C^{4} (153.4 ppm)
H$^{1'}$ (6.35 ppm) ⟷ C^{8} (144.5 ppm)

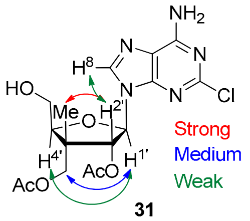

NOESY confirmed
β relative stereochemistry

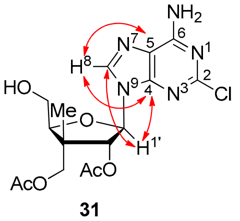

From HSQC

H^{8} (8.41 ppm) ⟷ C^{8} (141.6 ppm)
H$^{1'}$ (6.12 ppm) ⟷ C$^{1'}$ (151.7 ppm)

From HMBC

H^{8} (8.41 ppm) ⟷ C^{5} (119.4 ppm)
H^{8} (8.41 ppm) ⟷ C^{4} (151.7 ppm)
H$^{1'}$ (6.12 ppm) ⟷ C^{8} (141.6 ppm)
H$^{1'}$ (6.12 ppm) ⟷ C^{4} (151.7 ppm)

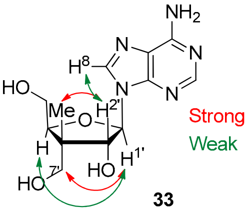

NOESY confirmed
β relative stereochemistry

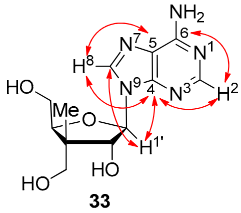

From HSQC

H^{8} (8.30 ppm) ⟷ C^{8} (142.4 ppm)
H^{2} (8.18 ppm) ⟷ C^{2} (153.3 ppm)
H$^{1'}$ (6.03 ppm) ⟷ C$^{1'}$ (91.4 ppm)

From HMBC

H^{8} (8.30 ppm) ⟷ C^{5} (121.1 ppm)
H^{8} (8.30 ppm) ⟷ C^{4} (149.9 ppm)
H^{2} (8.18 ppm) ⟷ C^{6} (157.6 ppm)
H^{2} (8.18 ppm) ⟷ C^{4} (149.9 ppm)
H$^{1'}$ (6.03 ppm) ⟷ C^{4} (149.9 ppm)
H$^{1'}$ (6.03 ppm) ⟷ C^{8} (142.4 ppm)

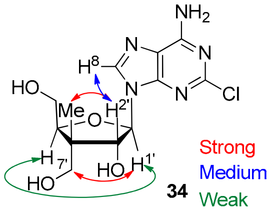

NOESY confirmed
β relative stereochemistry

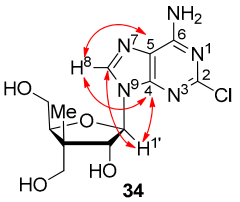

From HSQC

H^{8} (8.30 ppm) ⟷ C^{8} (142.4 ppm)
H$^{1'}$ (5.98 ppm) ⟷ C$^{1'}$ (91.1 ppm)

From HMBC

H^{8} (8.30 ppm) ⟷ C^{5} (119.9 ppm)
H^{8} (8.30 ppm) ⟷ C^{4} (151.4 ppm)
H$^{1'}$ (5.98 ppm) ⟷ C^{8} (142.4 ppm)
H$^{1'}$ (5.98 ppm) ⟷ C^{4} (151.4 ppm)

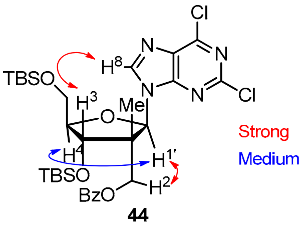

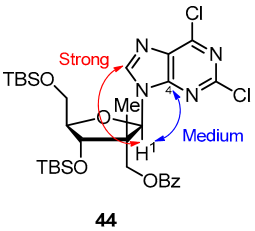

From HSQC

H^{1} (6.61 ppm) ⟷ C^{1} (88.16 ppm)
H^{8} (8.90 ppm) ⟷ C^{8} (145.00 ppm)

From HMBC

H^{1} (6.60 ppm) ⟷ C^{4} (152.83 ppm)
H^{1} (6.60 ppm) ⟷ C^{8} (145.00 ppm)

^{1}H-NMR (500 MHz, $CDCl_3$)

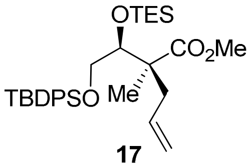

17

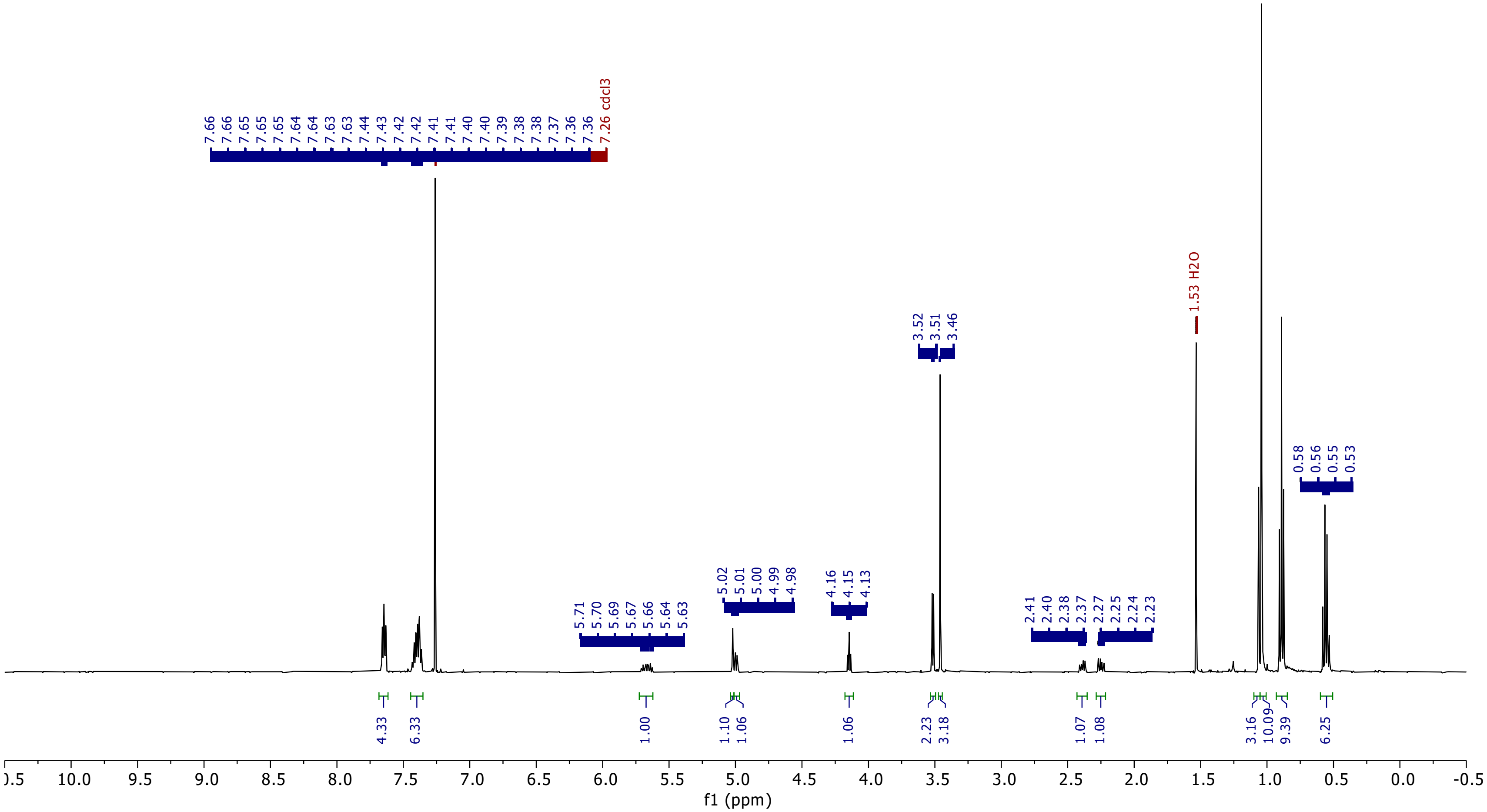

$^{13}C\{^{1}H\}$-NMR (126 MHz, $CDCl_3$)

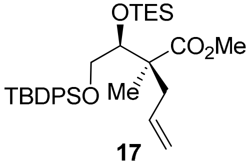

17

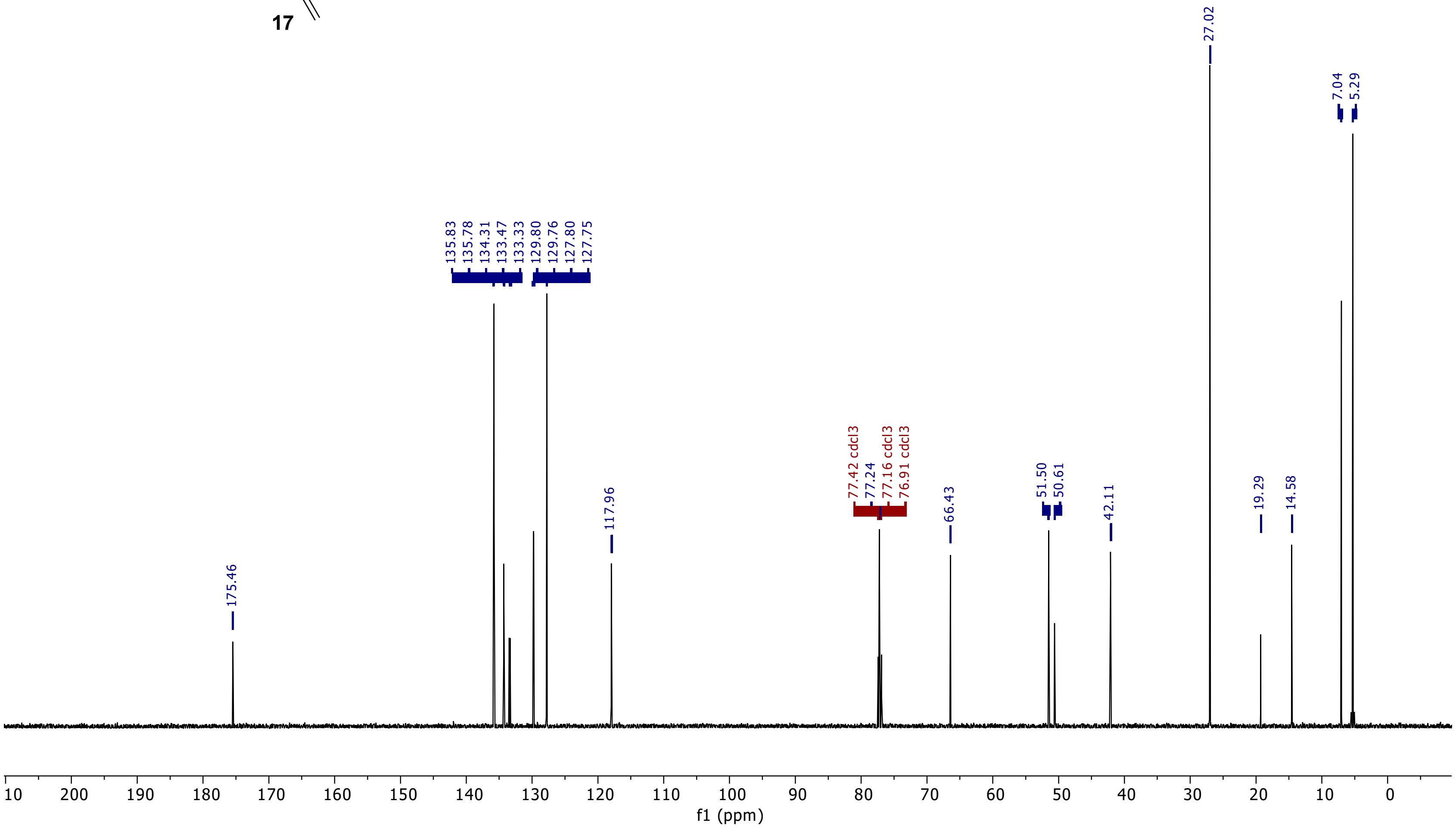

^{1}H-NMR (500 MHz, $CDCl_3$)

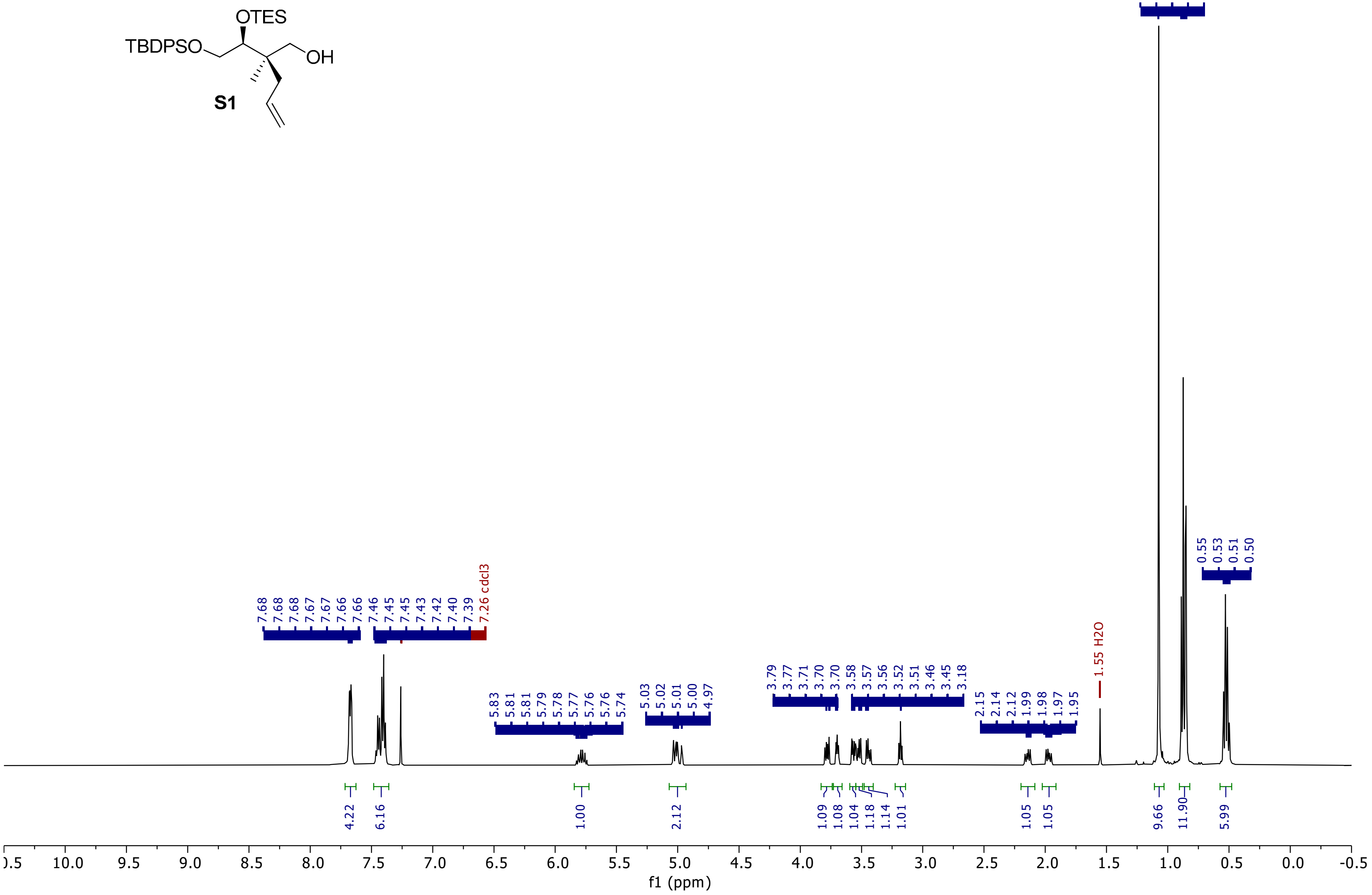

$^{13}C\{^{1}H\}$-NMR (126 MHz, $CDCl_3$)

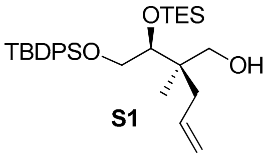

S1

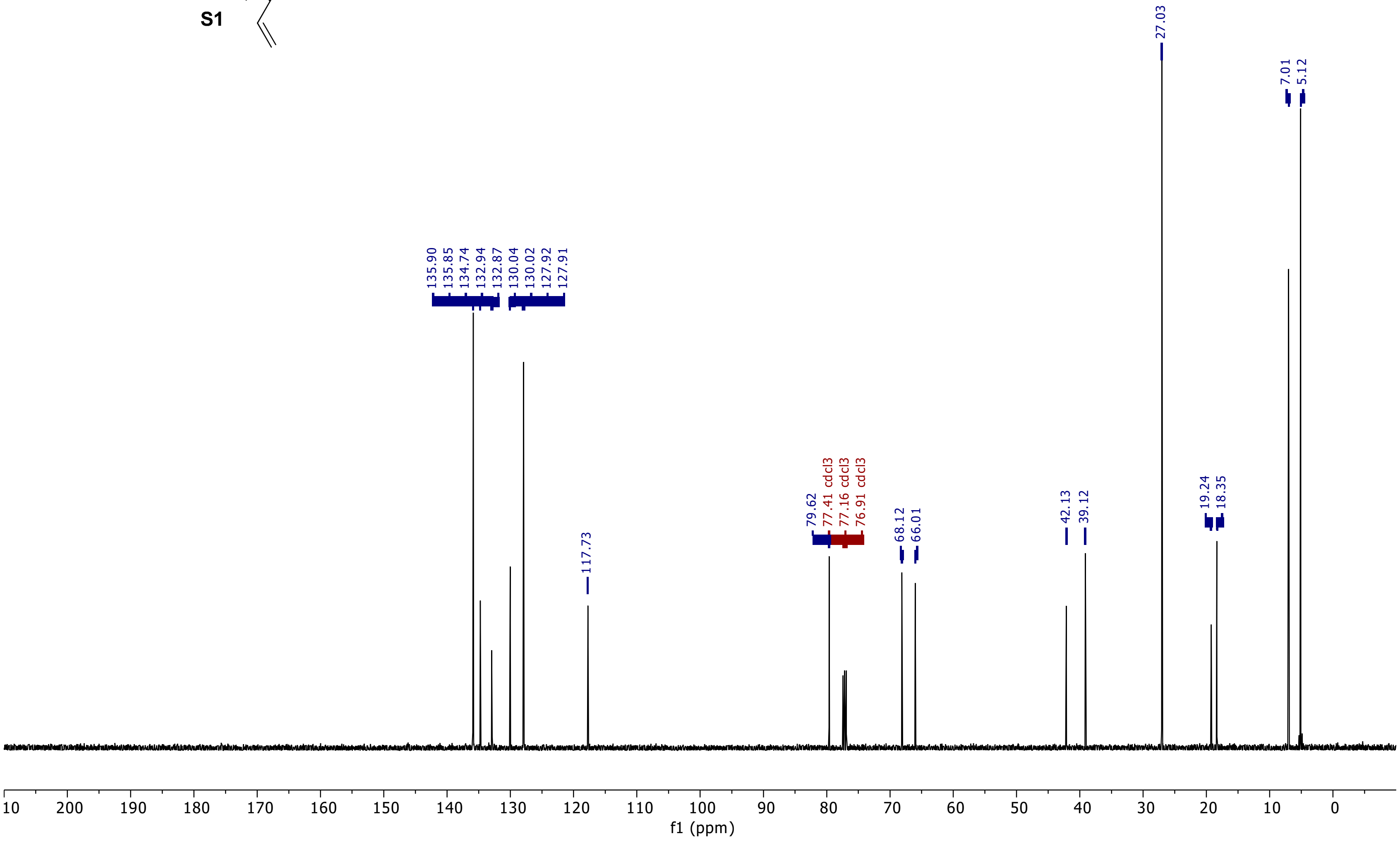

^{1}H-NMR (500 MHz, $CDCl_3$)

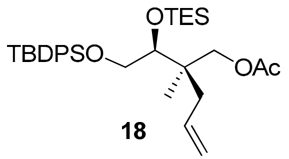

18

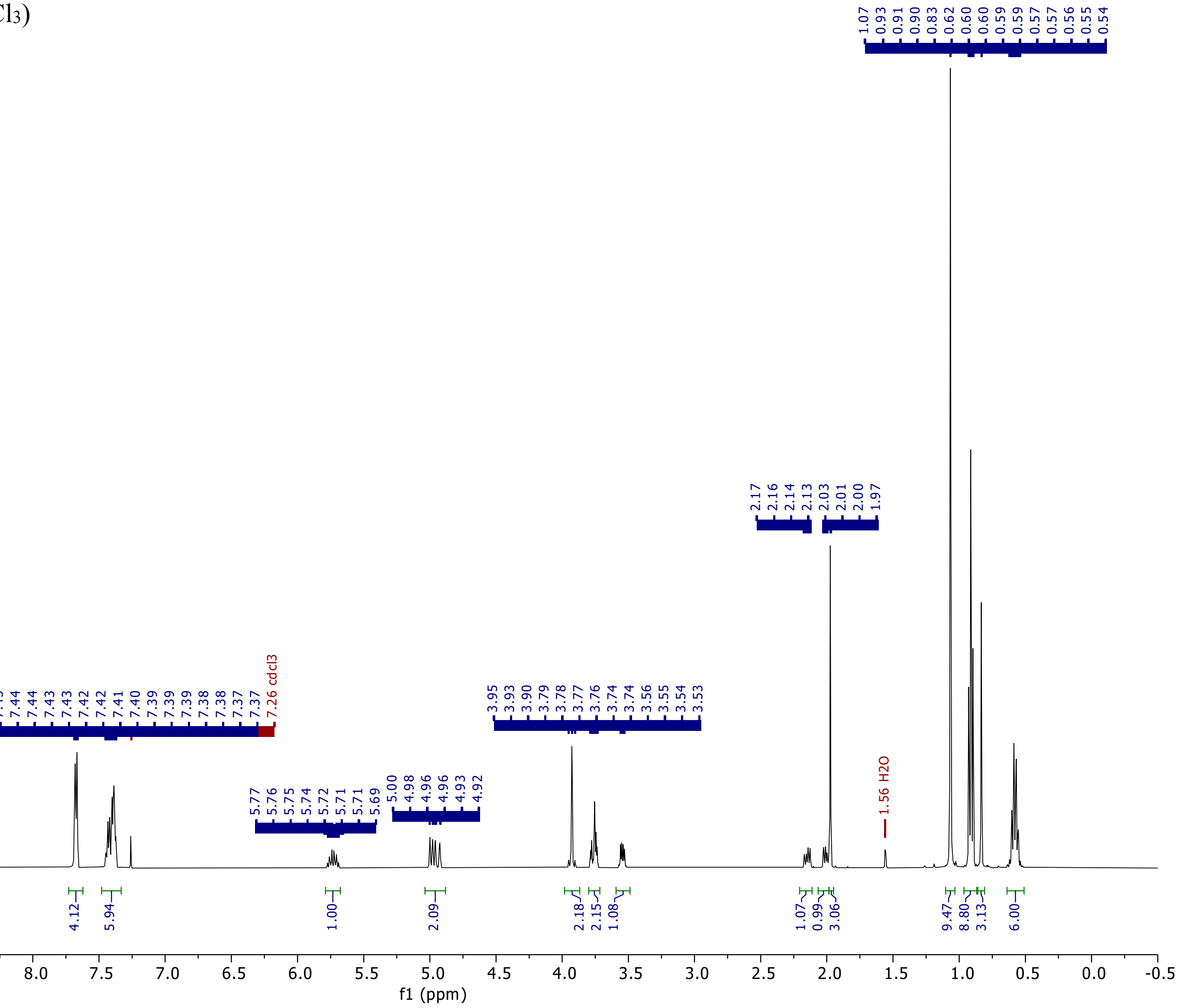

$^{13}C\{^{1}H\}$-NMR (126 MHz, $CDCl_3$)

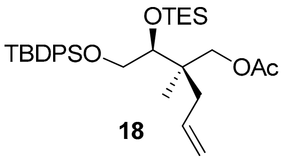

18

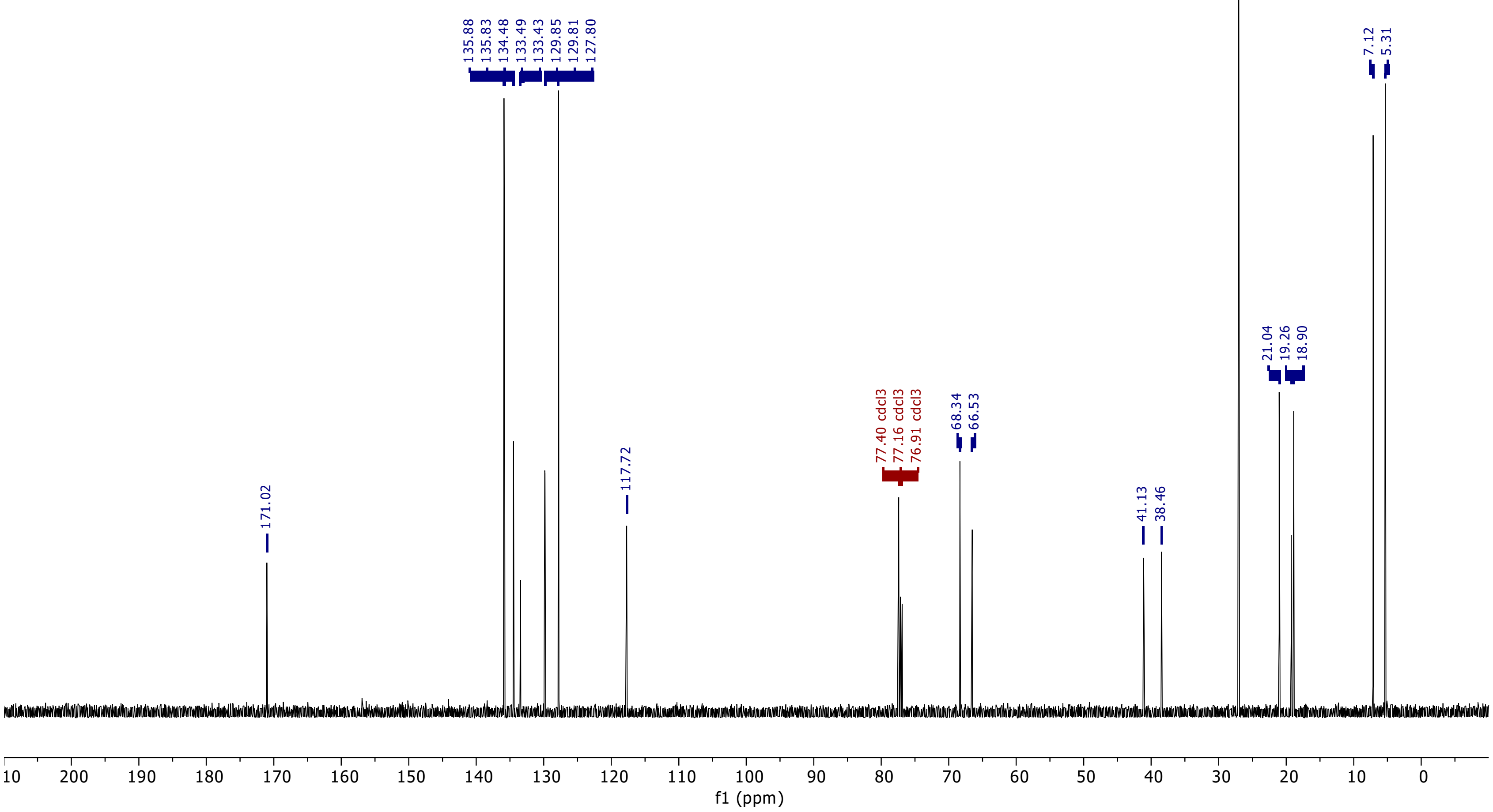

^{1}H-NMR (500 MHz, $CDCl_3$)

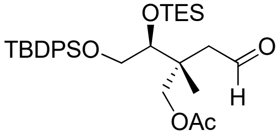

19

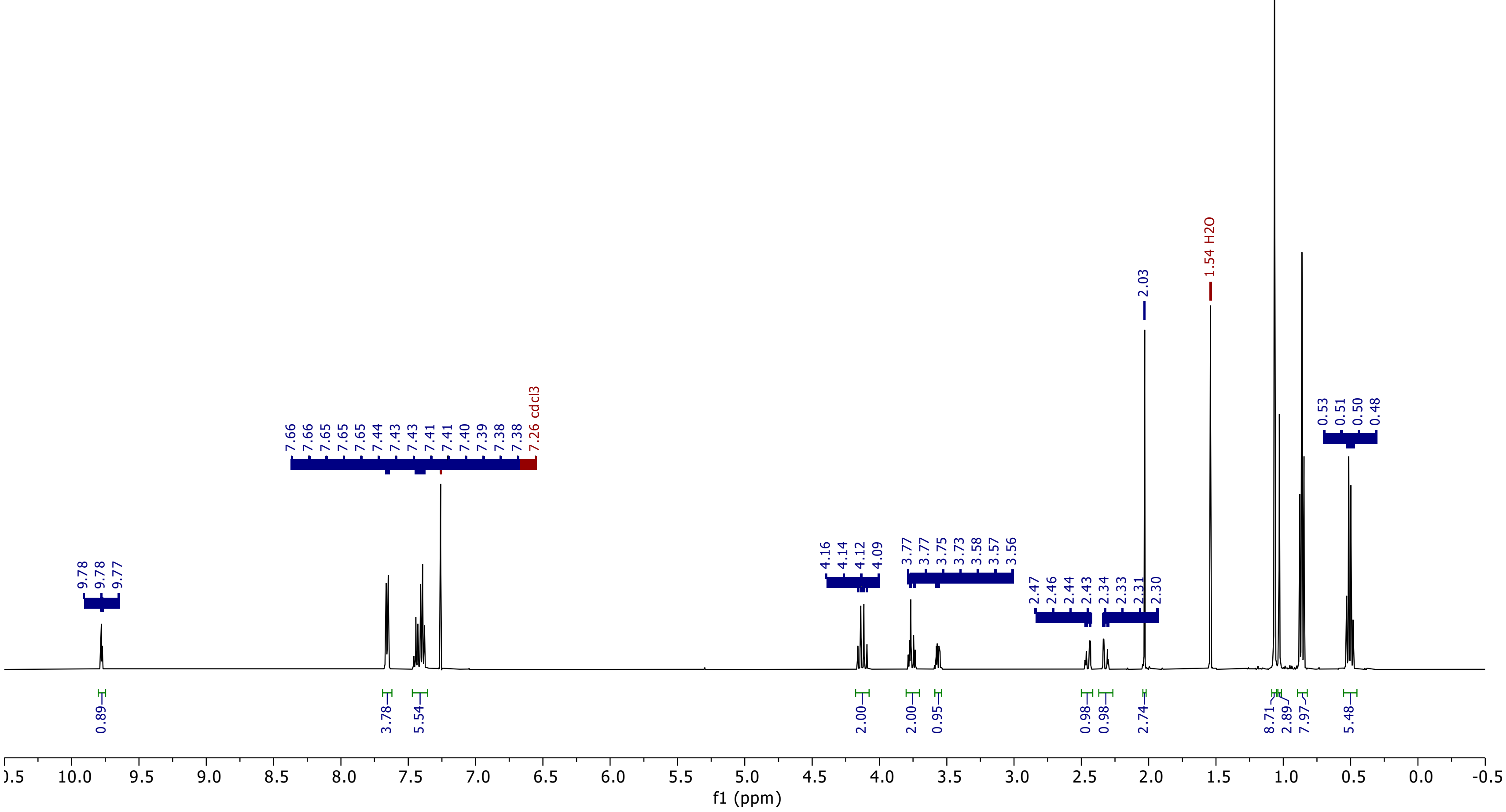

$^{13}C\{^{1}H\}$-NMR (126 MHz, $CDCl_3$)

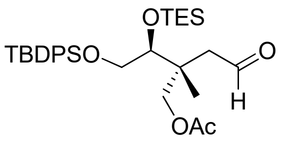

19

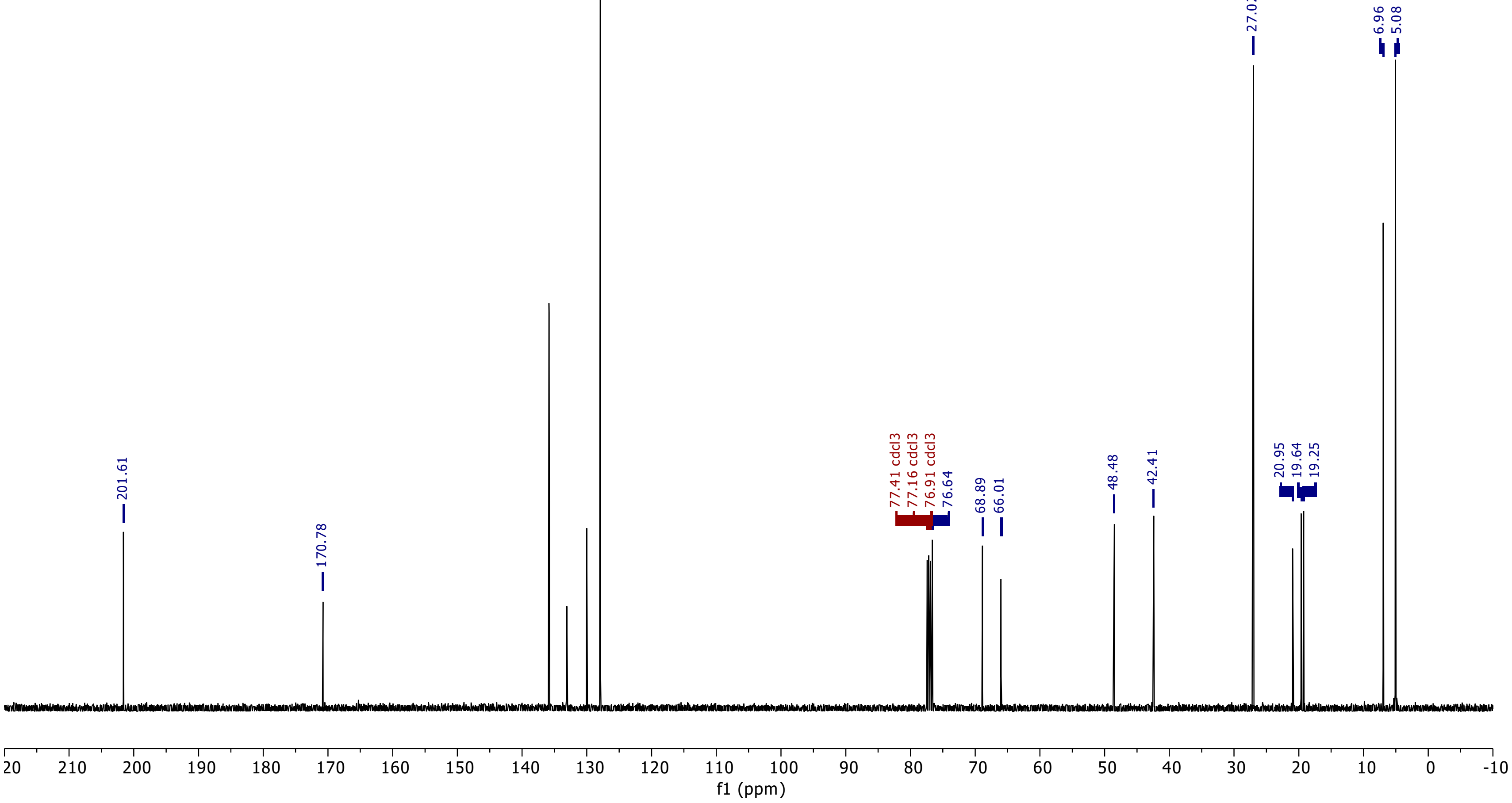

^{1}H-NMR (500 MHz, $CDCl_3$)

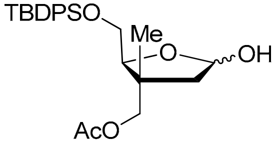

20a,b

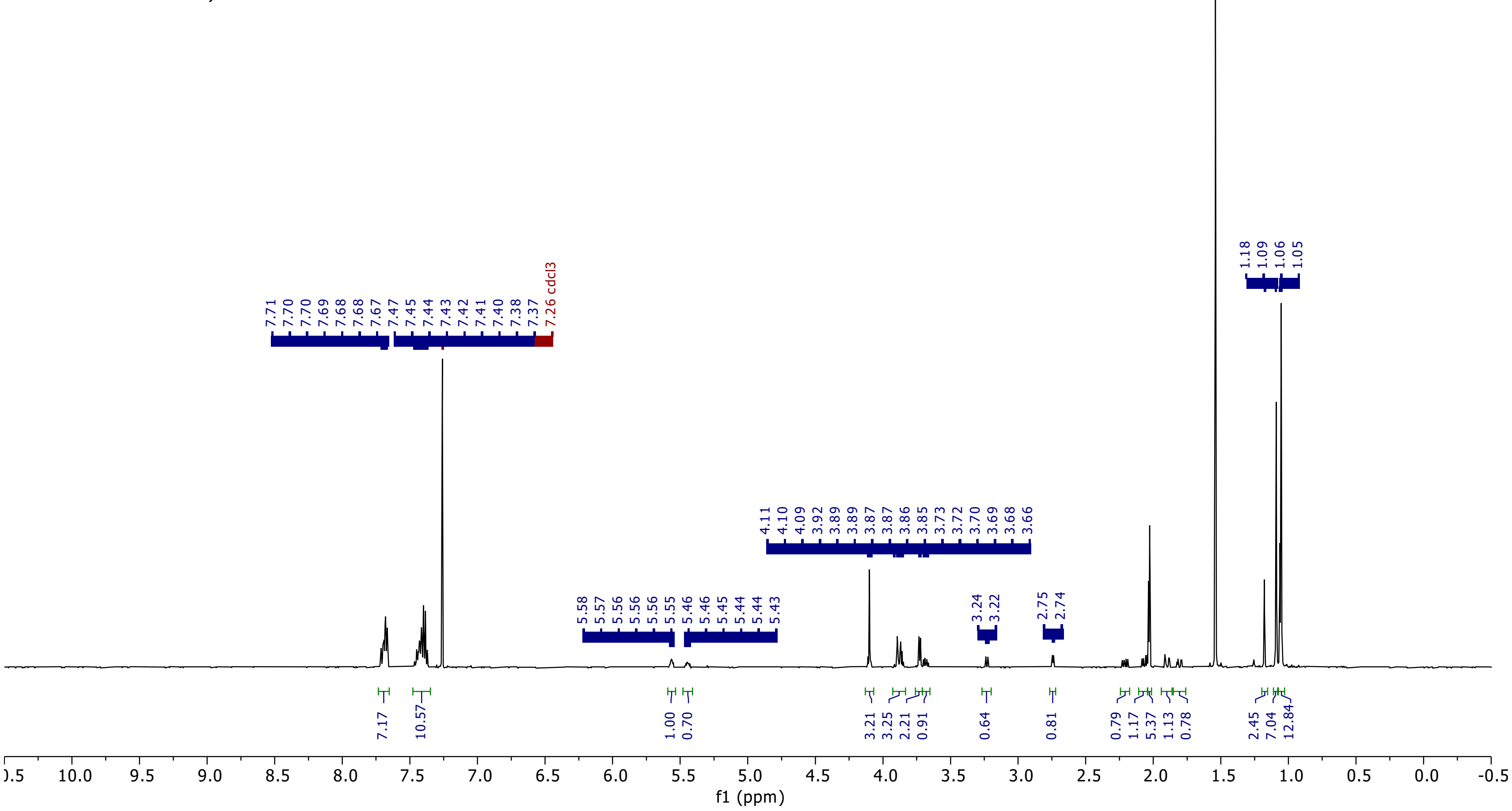

$^{13}C\{^1H\}$-NMR (126 MHz, $CDCl_3$)

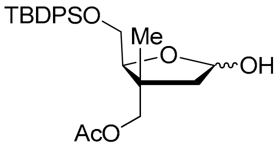

20a,b

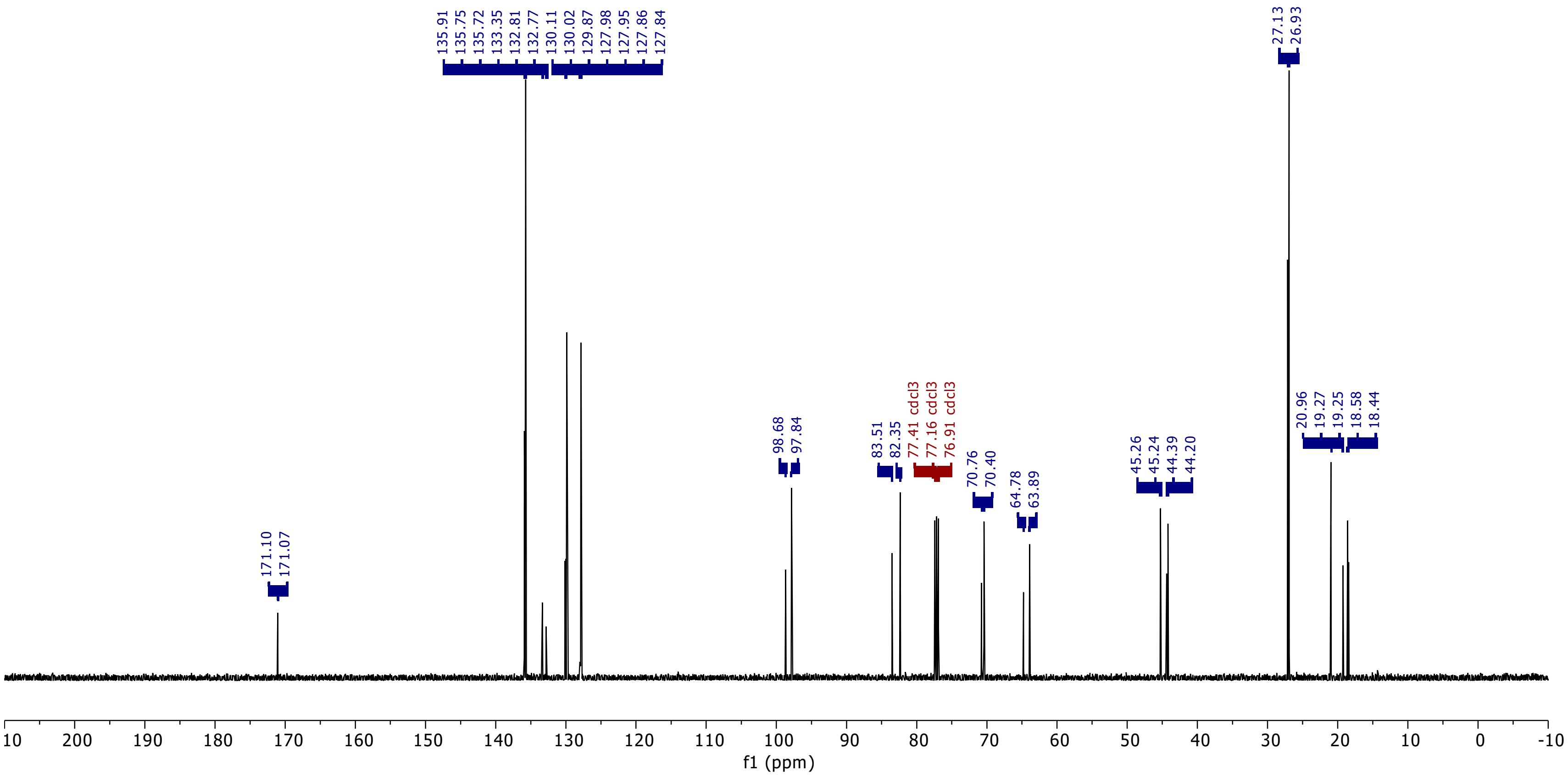

^{1}H-NMR (500 MHz, $CDCl_3$)

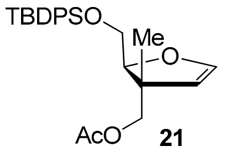

21

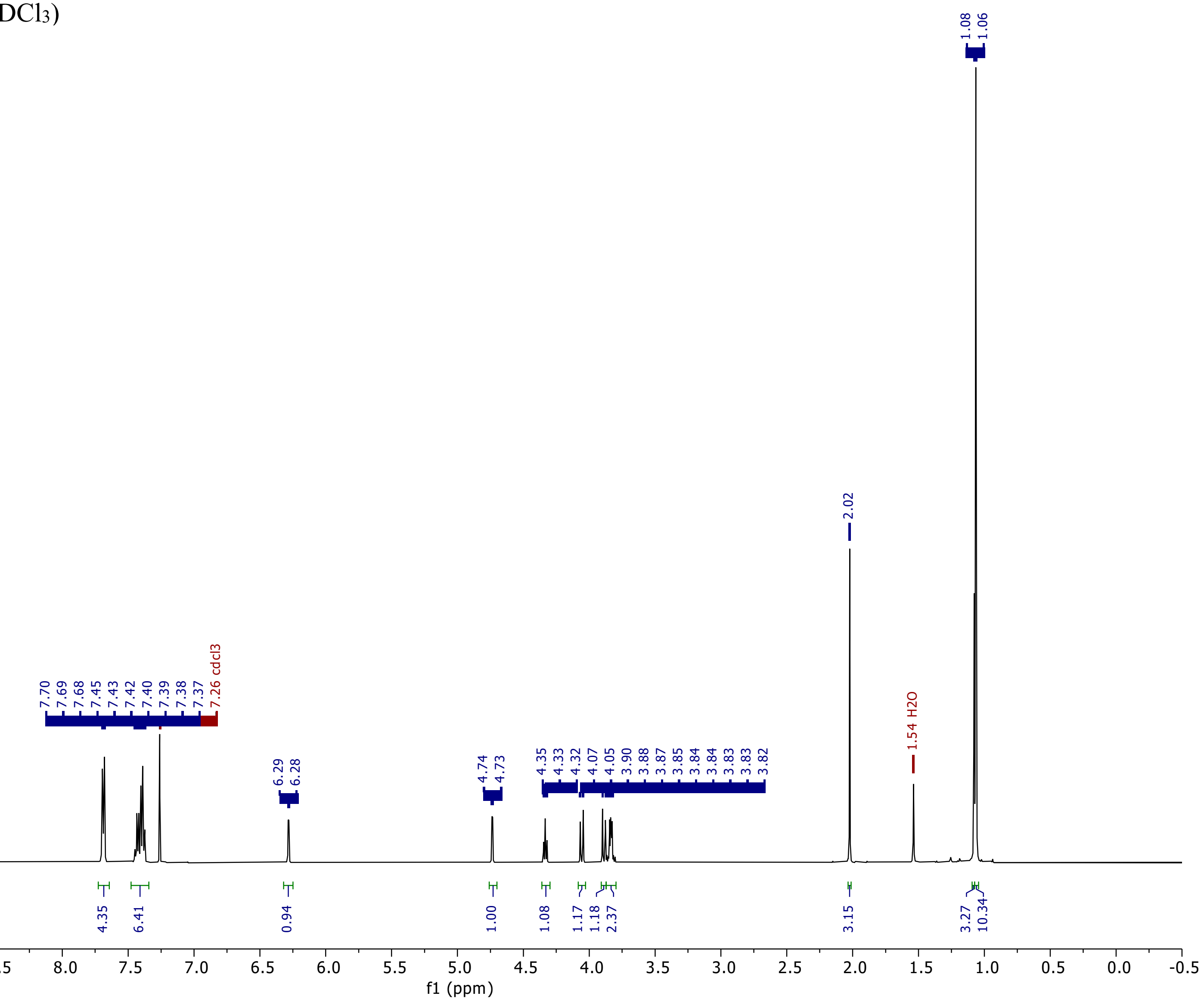

$^{13}C\{^{1}H\}$-NMR (126 MHz, $CDCl_3$)

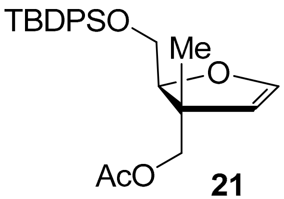

21

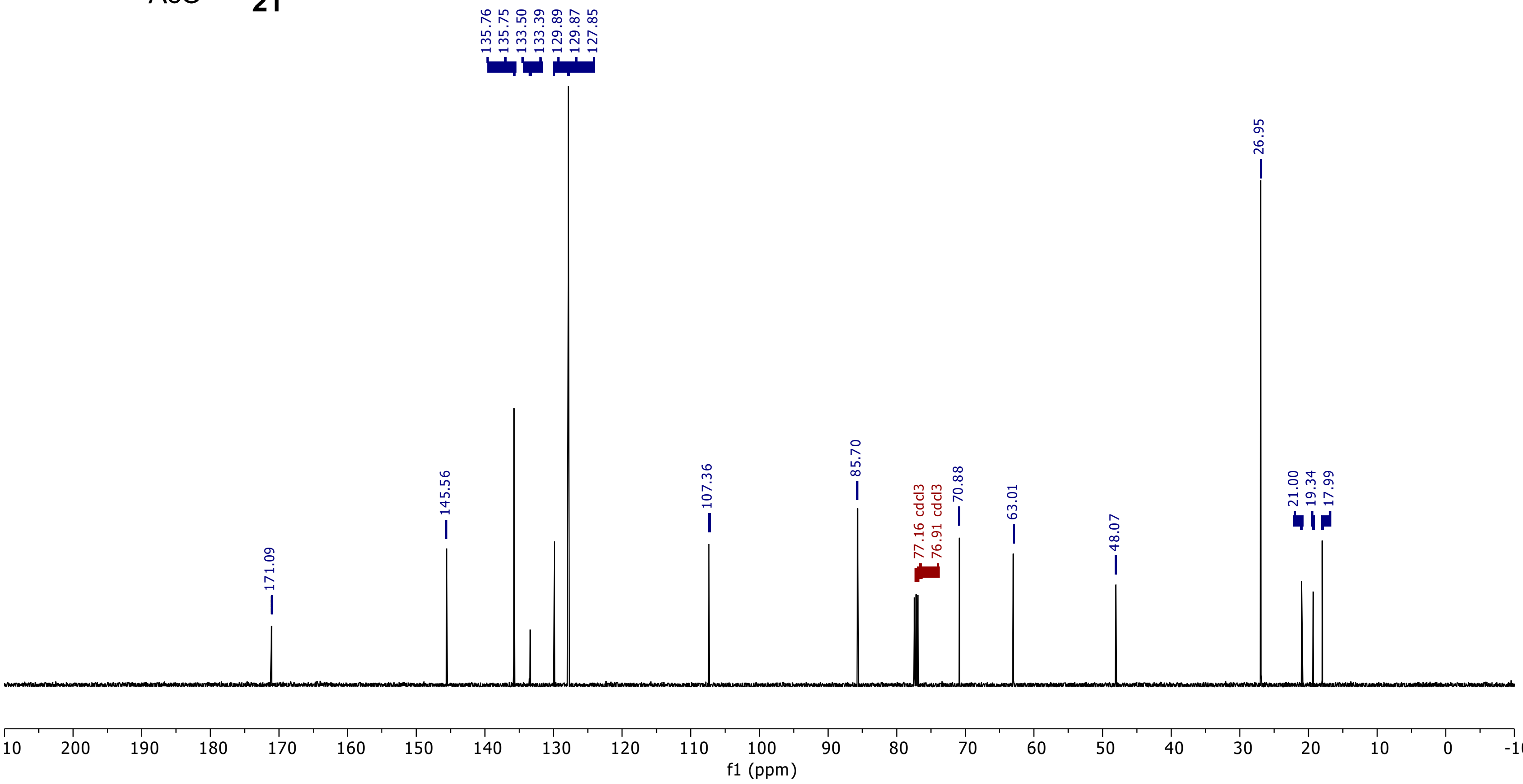

^{1}H-NMR (500 MHz, $CDCl_3$)

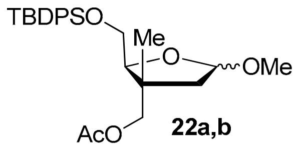

22a,b

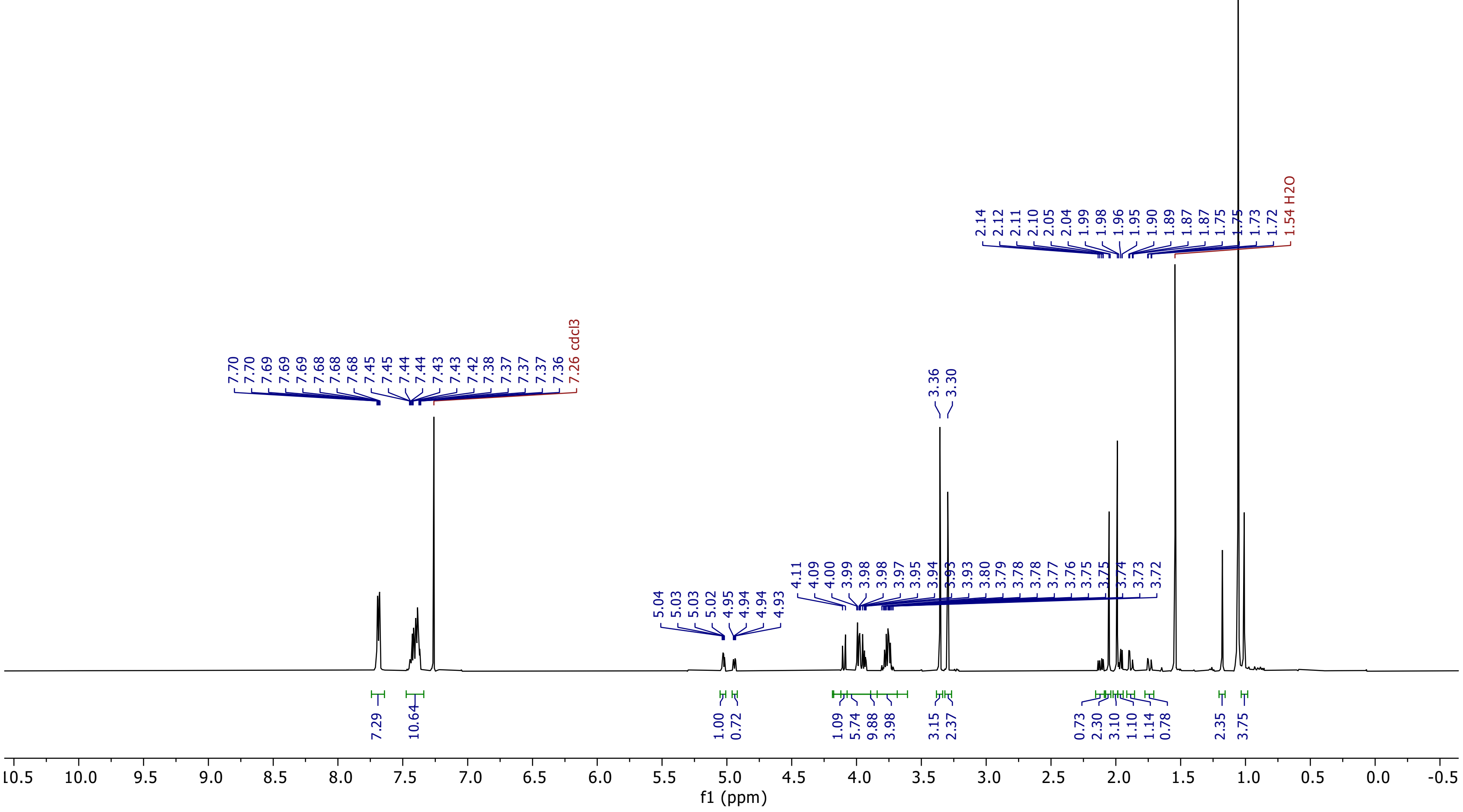

$^{13}C\{^1H\}$-NMR (126 MHz, $CDCl_3$)

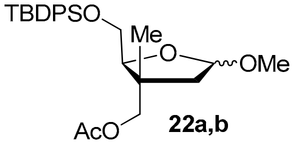

22a,b

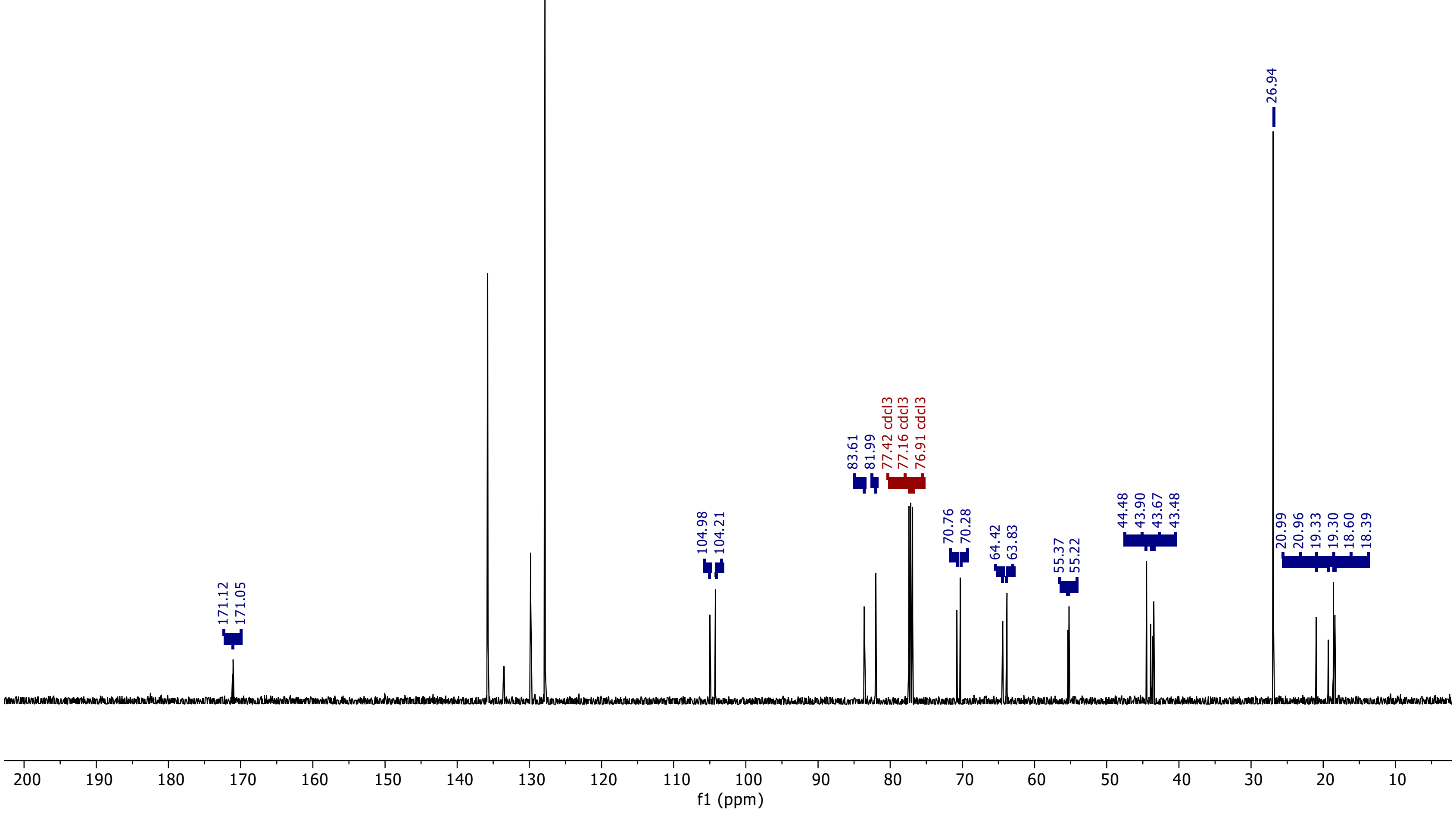

^{1}H-NMR (500 MHz, $CDCl_3$)

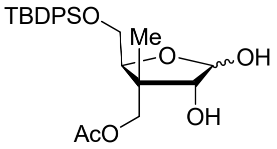

24a,b

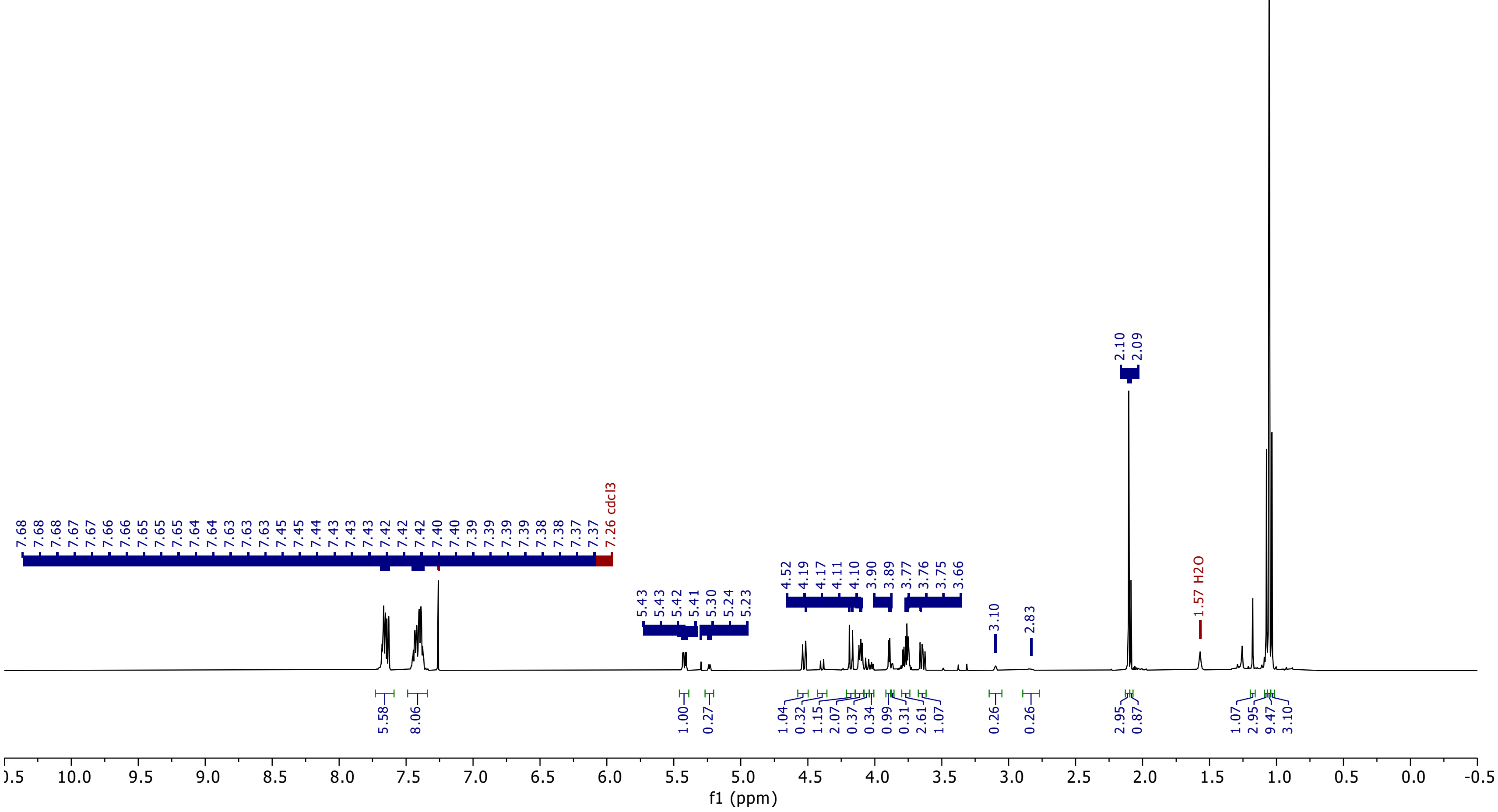

$^{13}C\{^{1}H\}$-NMR (126 MHz, $CDCl_3$)

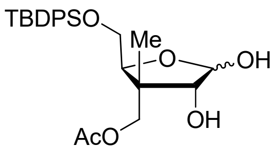

24a,b

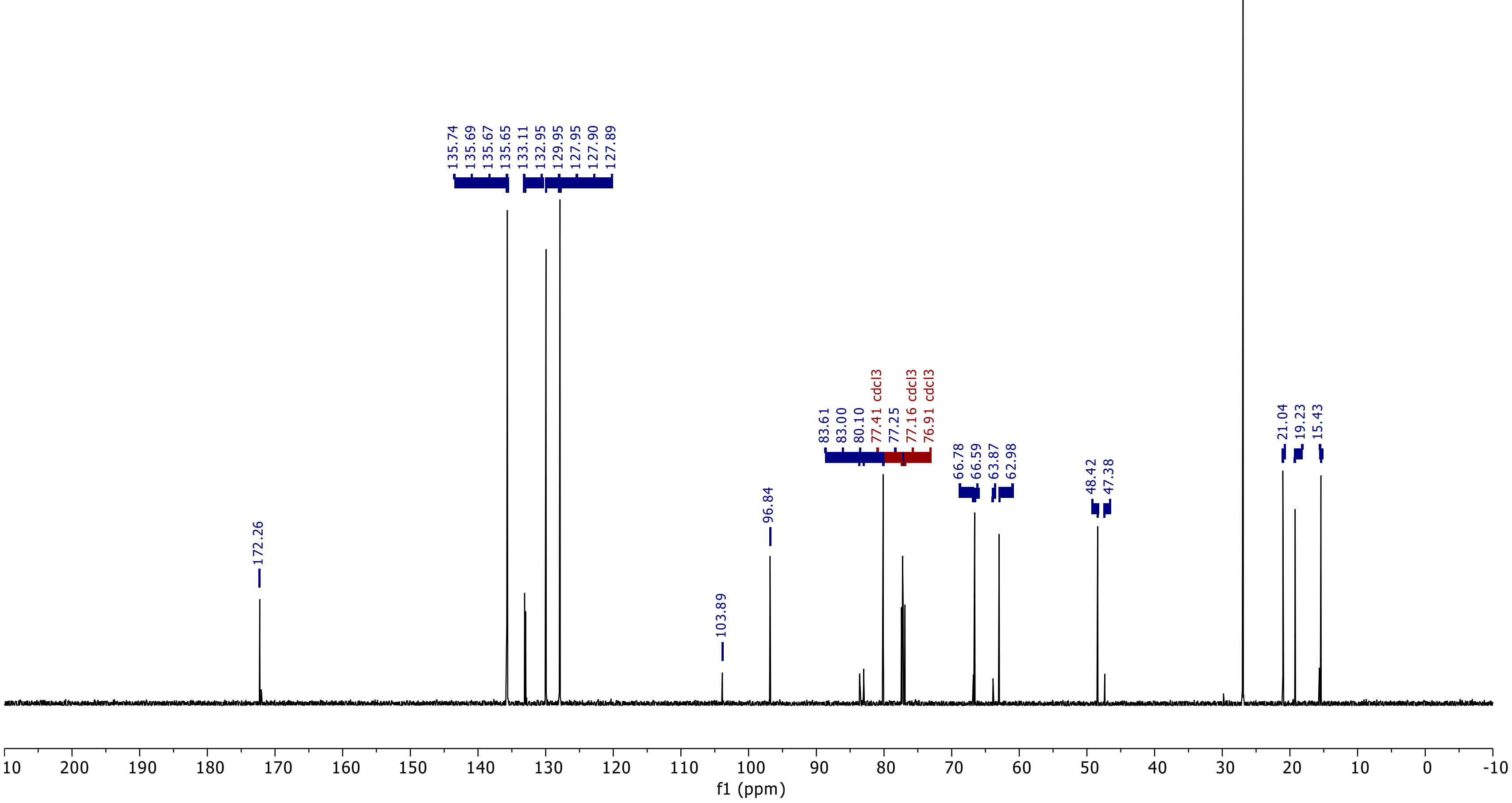

^{1}H-NMR (500 MHz, $CDCl_3$)

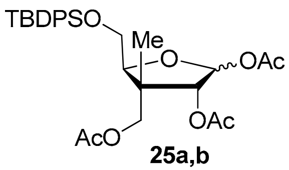

25a,b

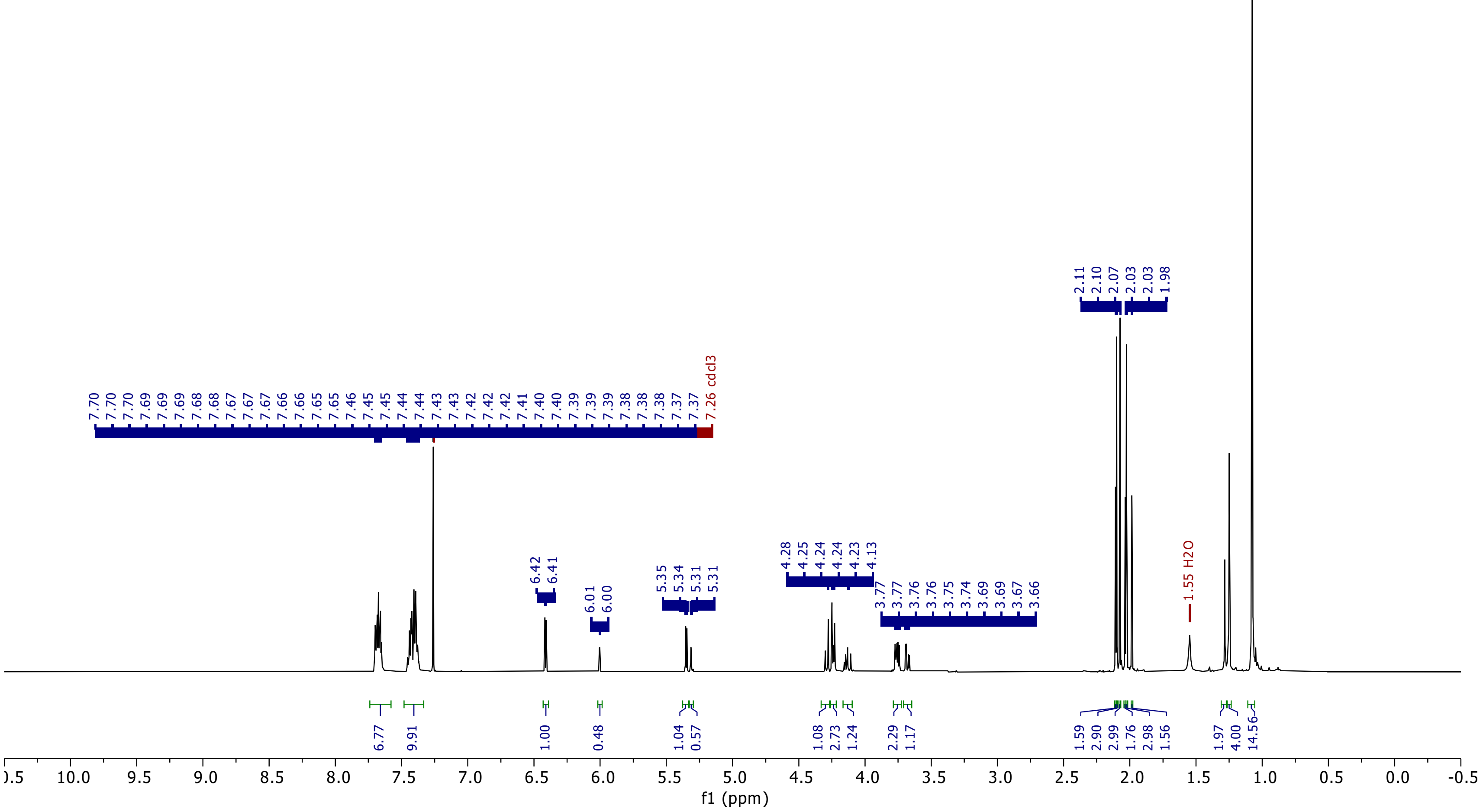

$^{13}C\{^{1}H\}$-NMR (126 MHz, $CDCl_3$)

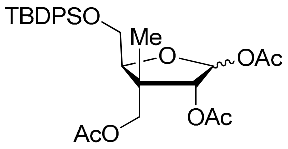

25a,b

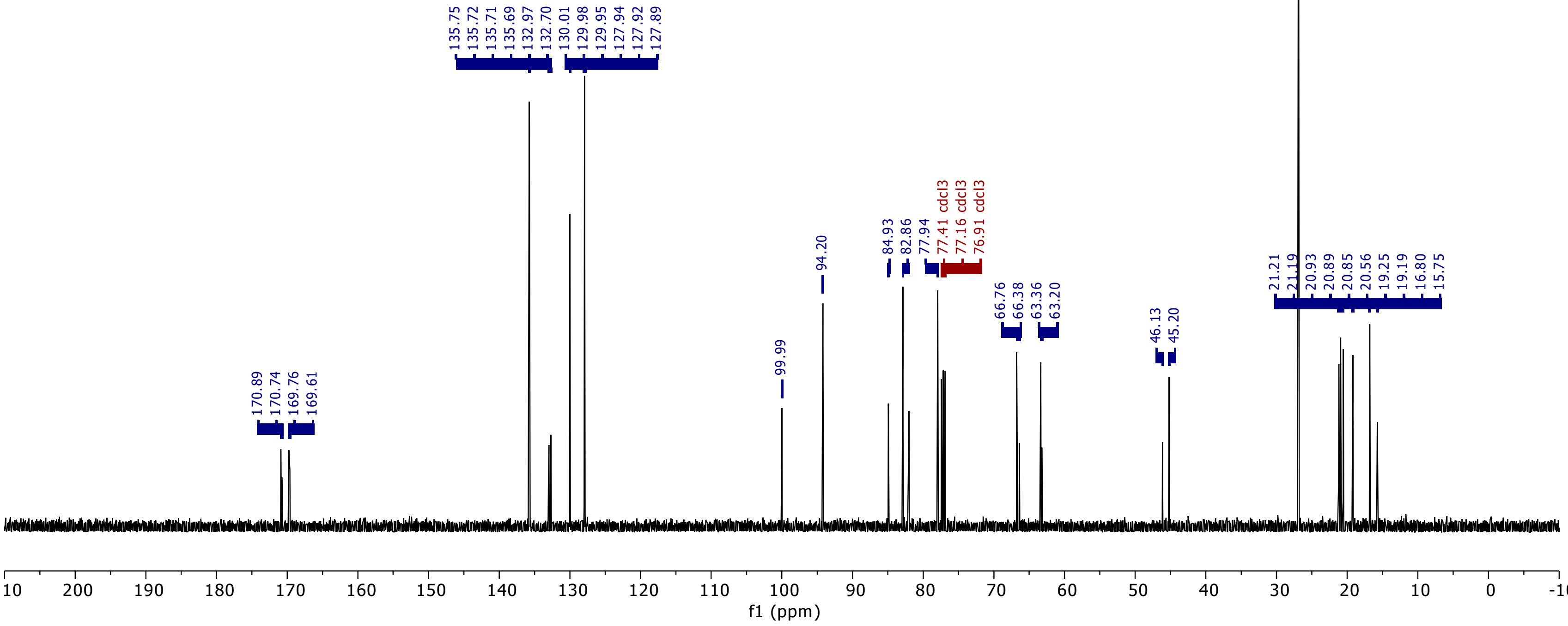

^{1}H-NMR (500 MHz, CD_3OD)

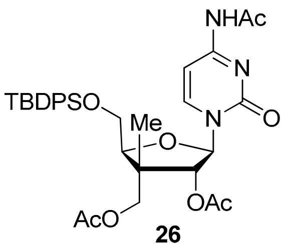

26

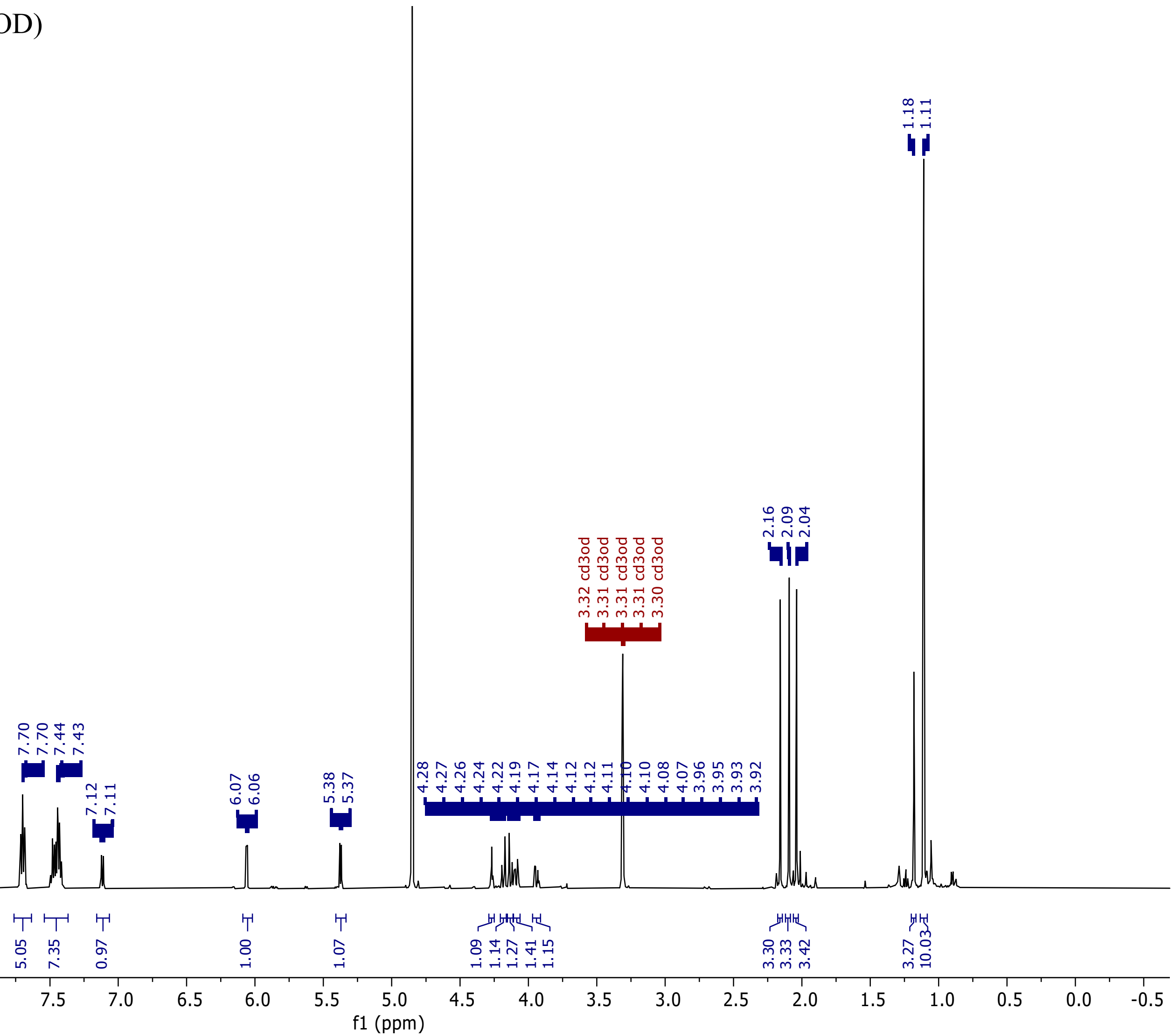

$^{13}C\{^{1}H\}$-NMR (126 MHz, CD_3OD)

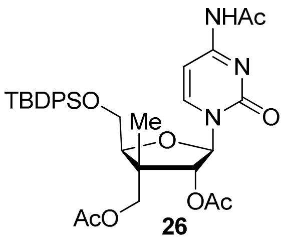

26

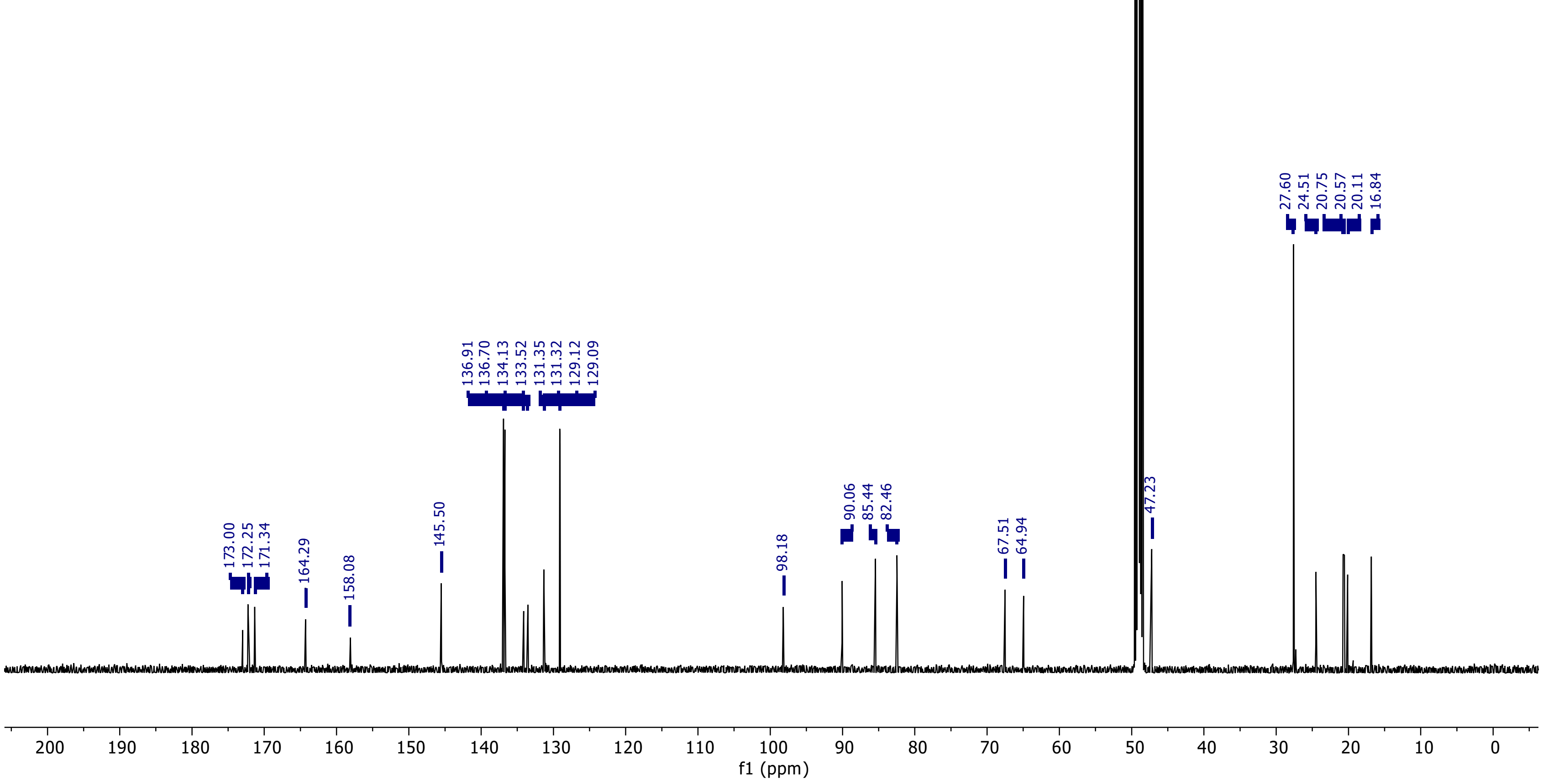

NOESY (500 MHz, CD_3OD)

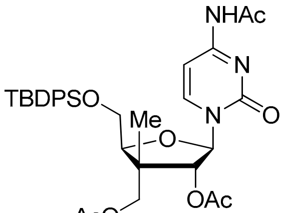

26

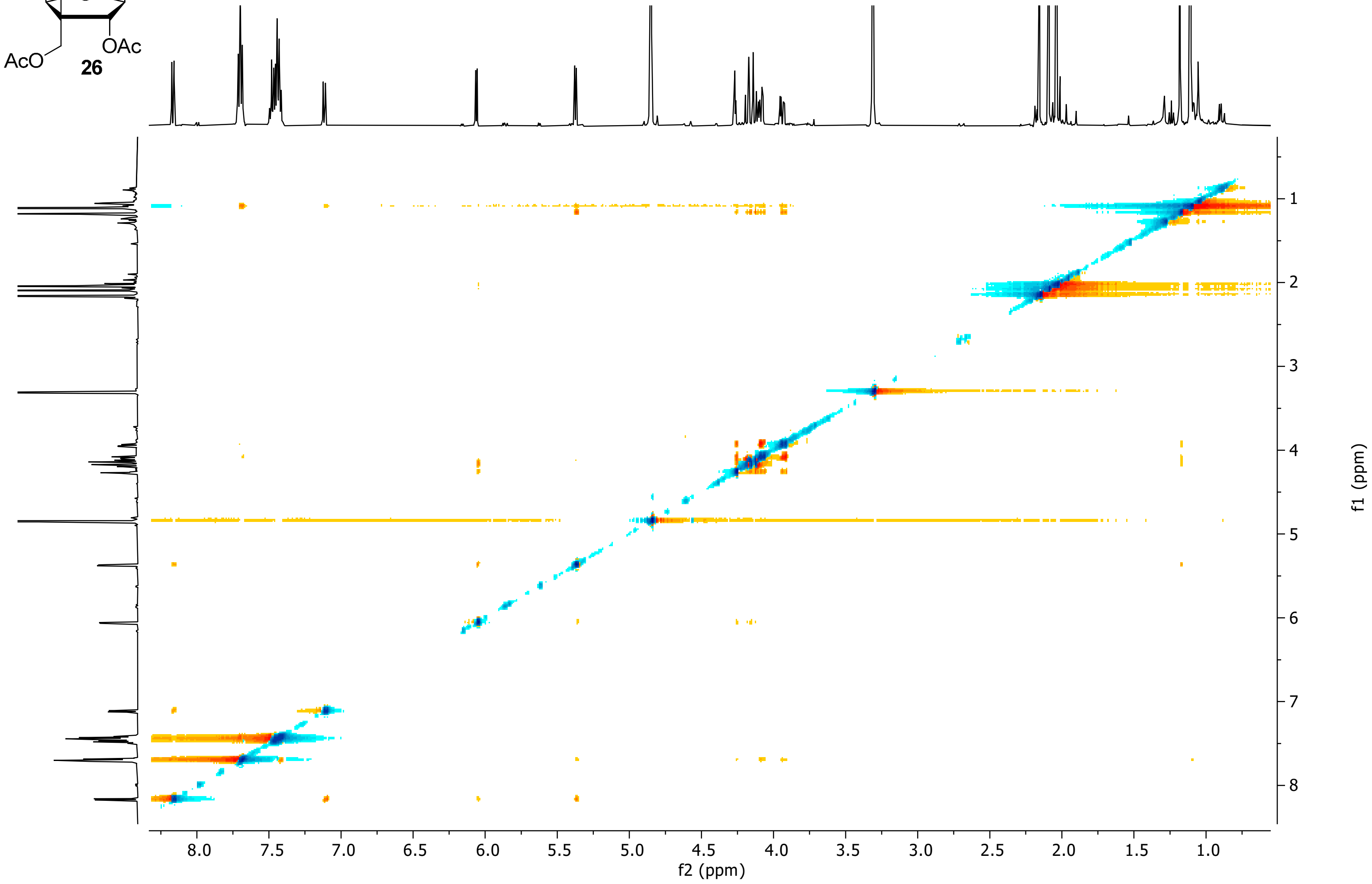

^{1}H-NMR (500 MHz, CD_3OD)

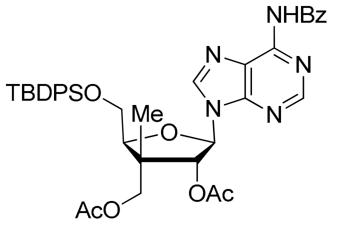

27

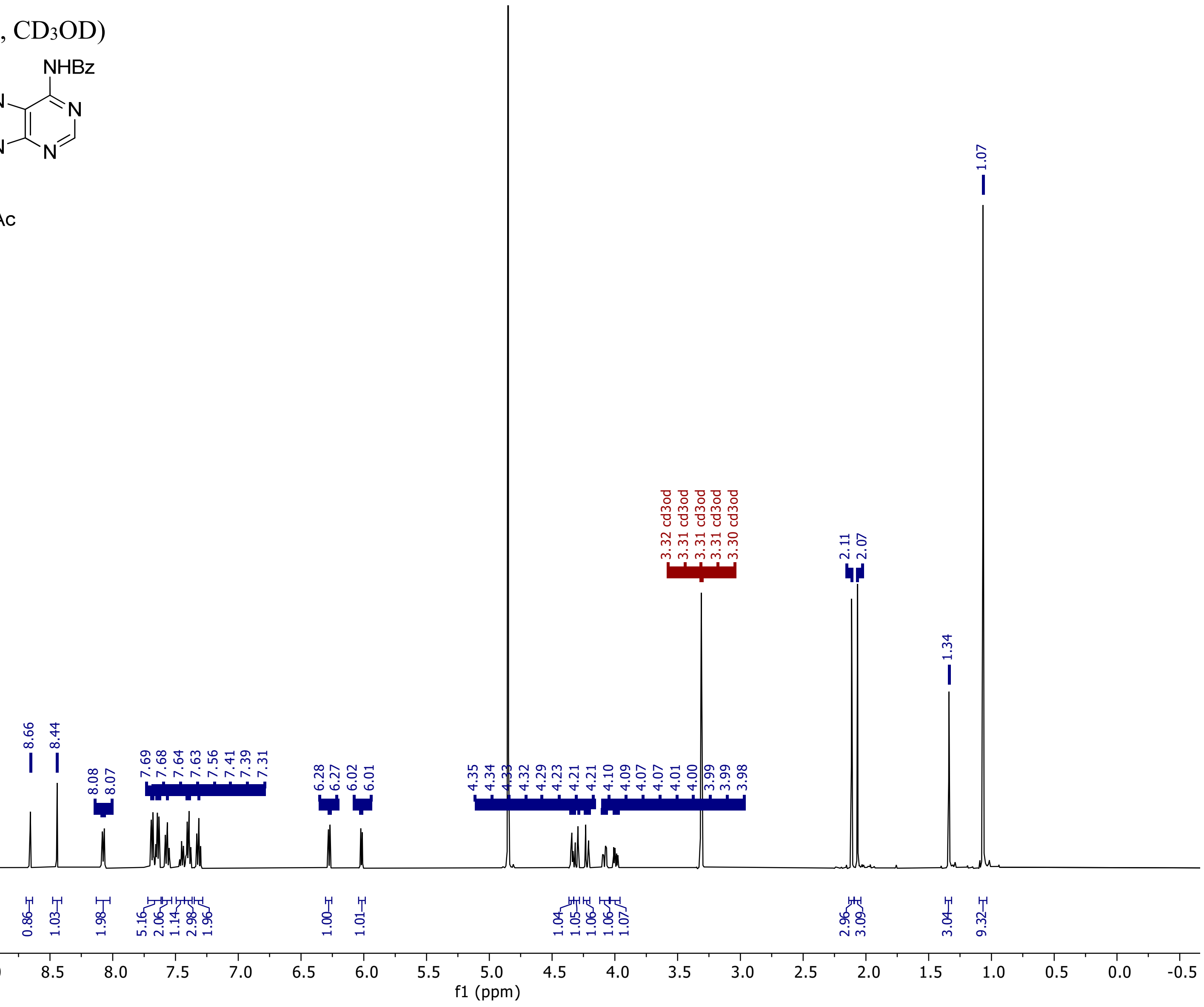

$^{13}C\{^{1}H\}$-NMR (126 MHz, CD_3OD)

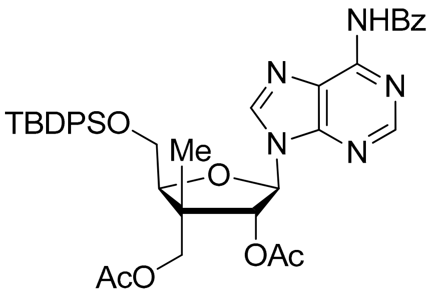

27

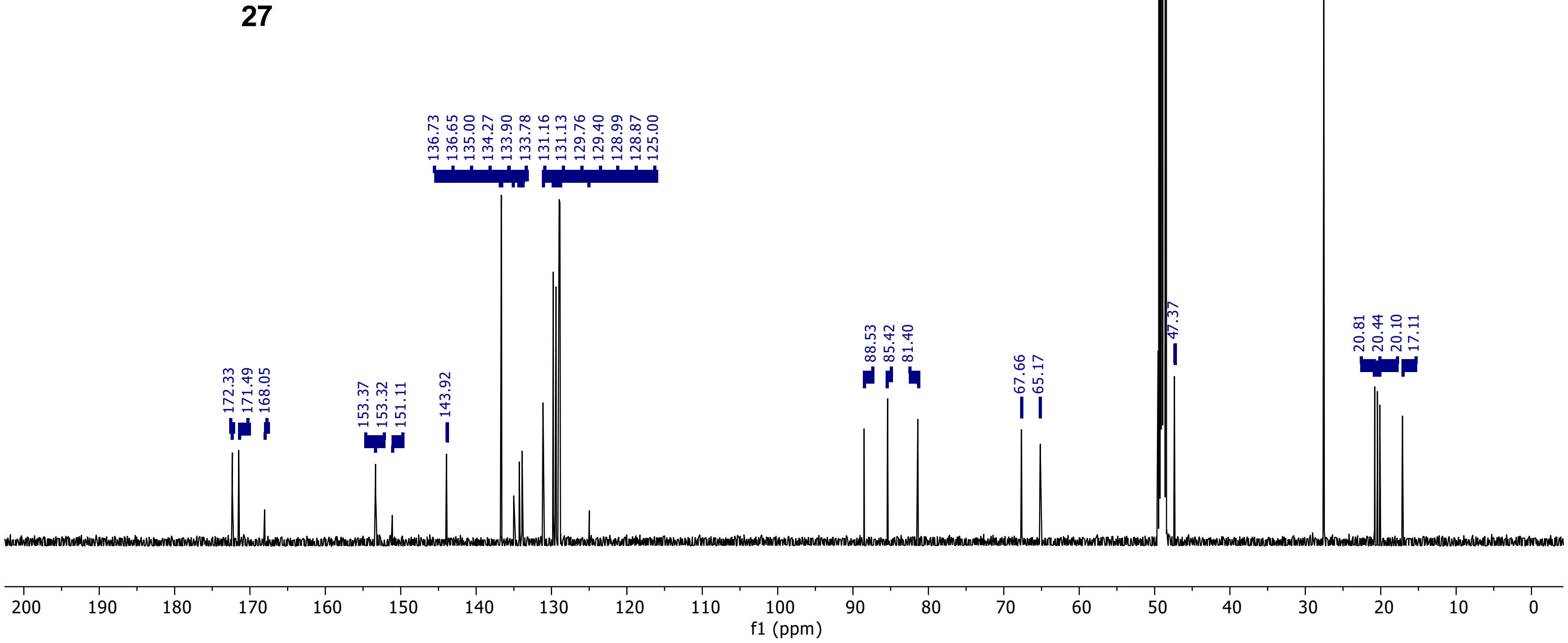

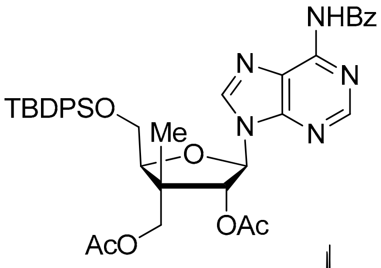

27

NOESY (500 MHz, CD3OD)

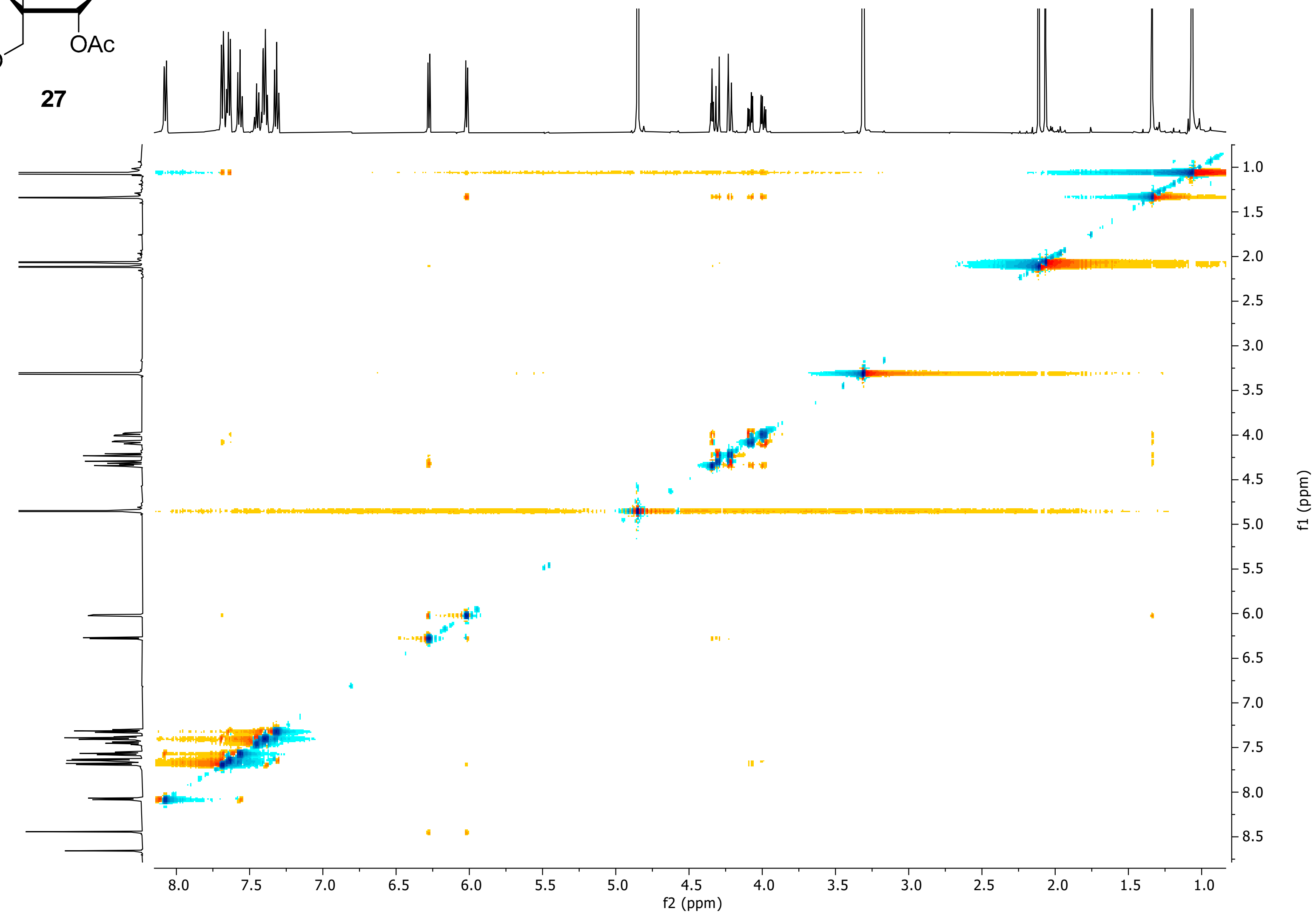

^{1}H-NMR (500 MHz, CD_3OD)

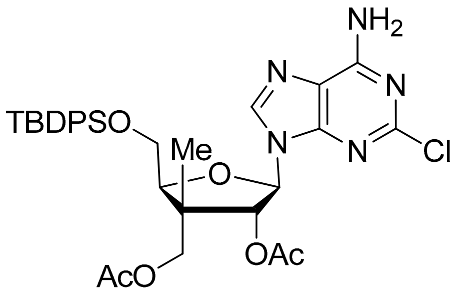

28

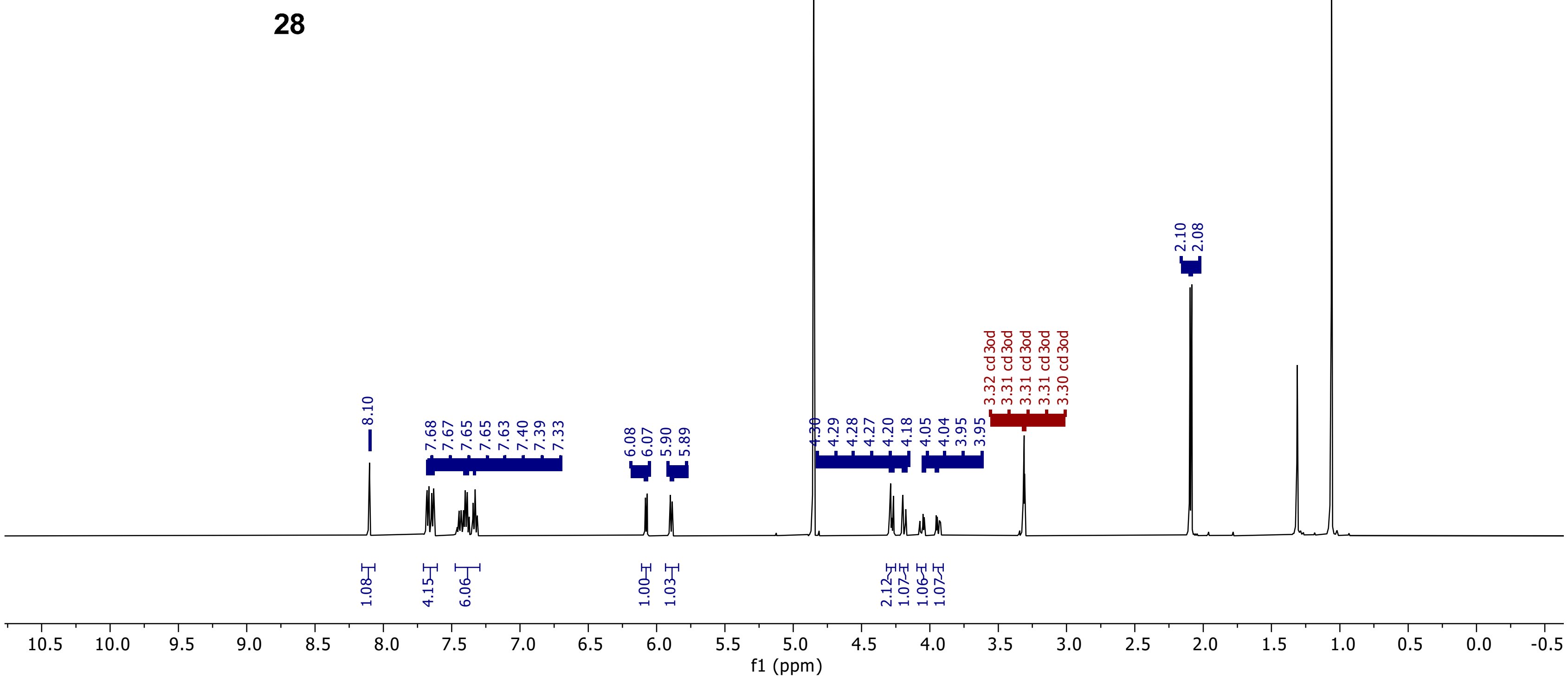

$^{13}C\{^{1}H\}$-NMR (126 MHz, CD_3OD)

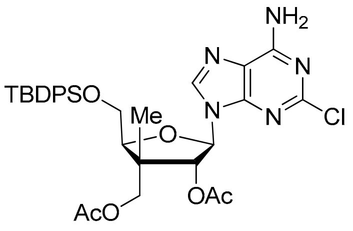

28

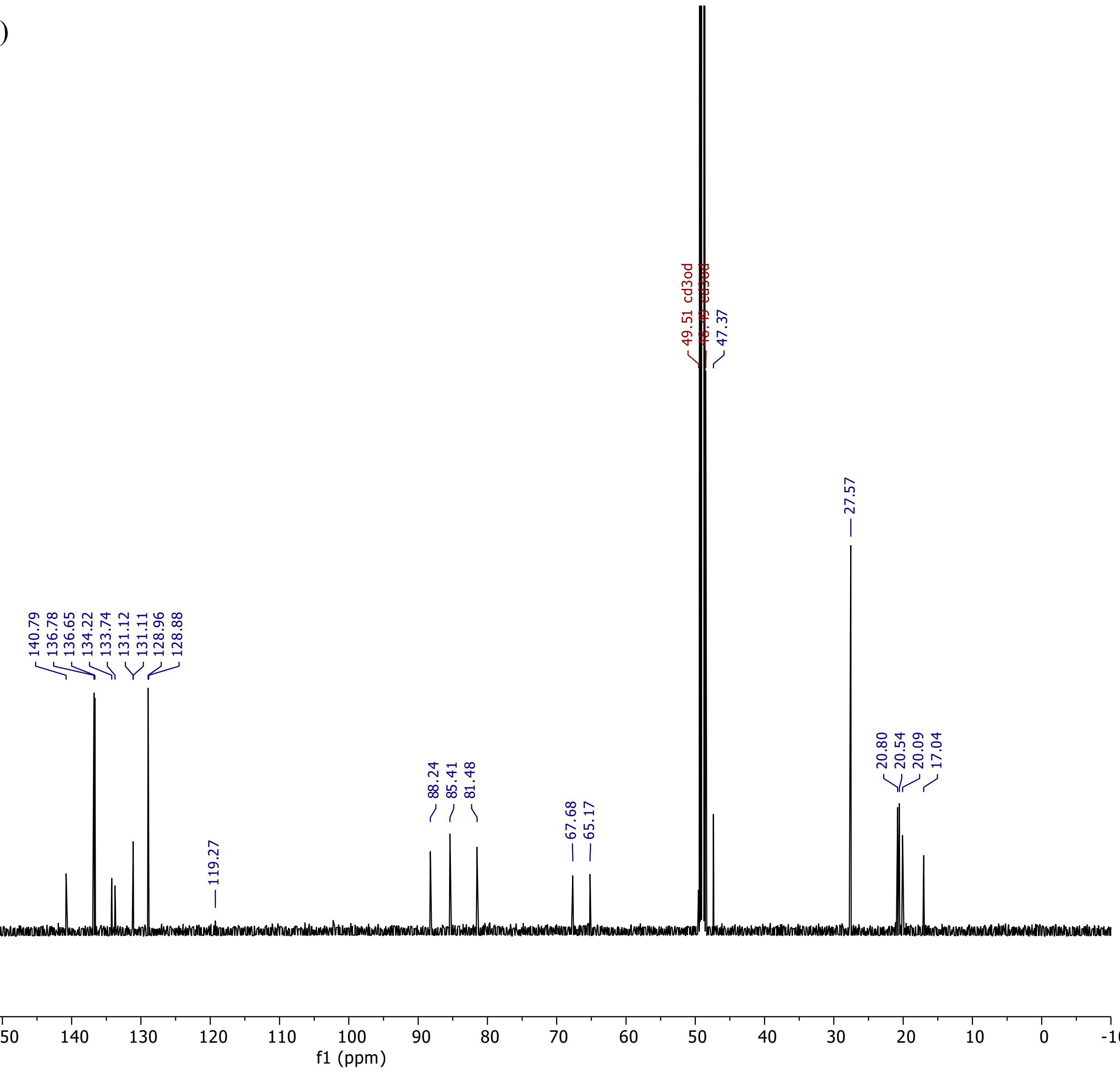

NOESY (500 MHz, CD_3OD)

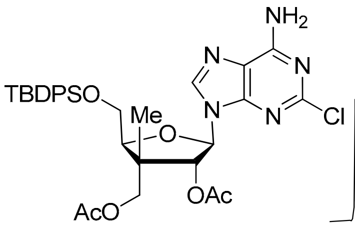

28

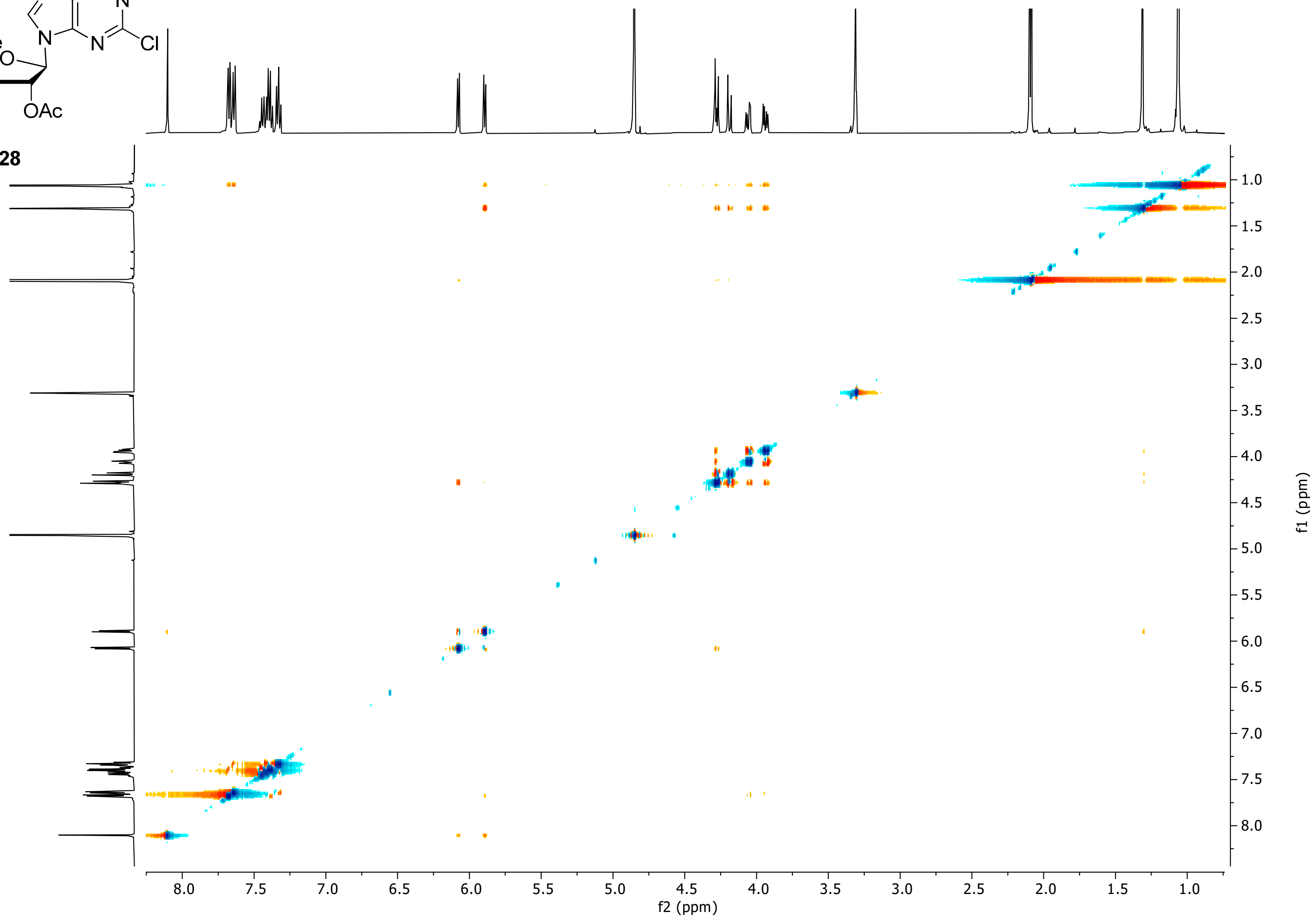

^{1}H-NMR (500 MHz, CD_3OD)

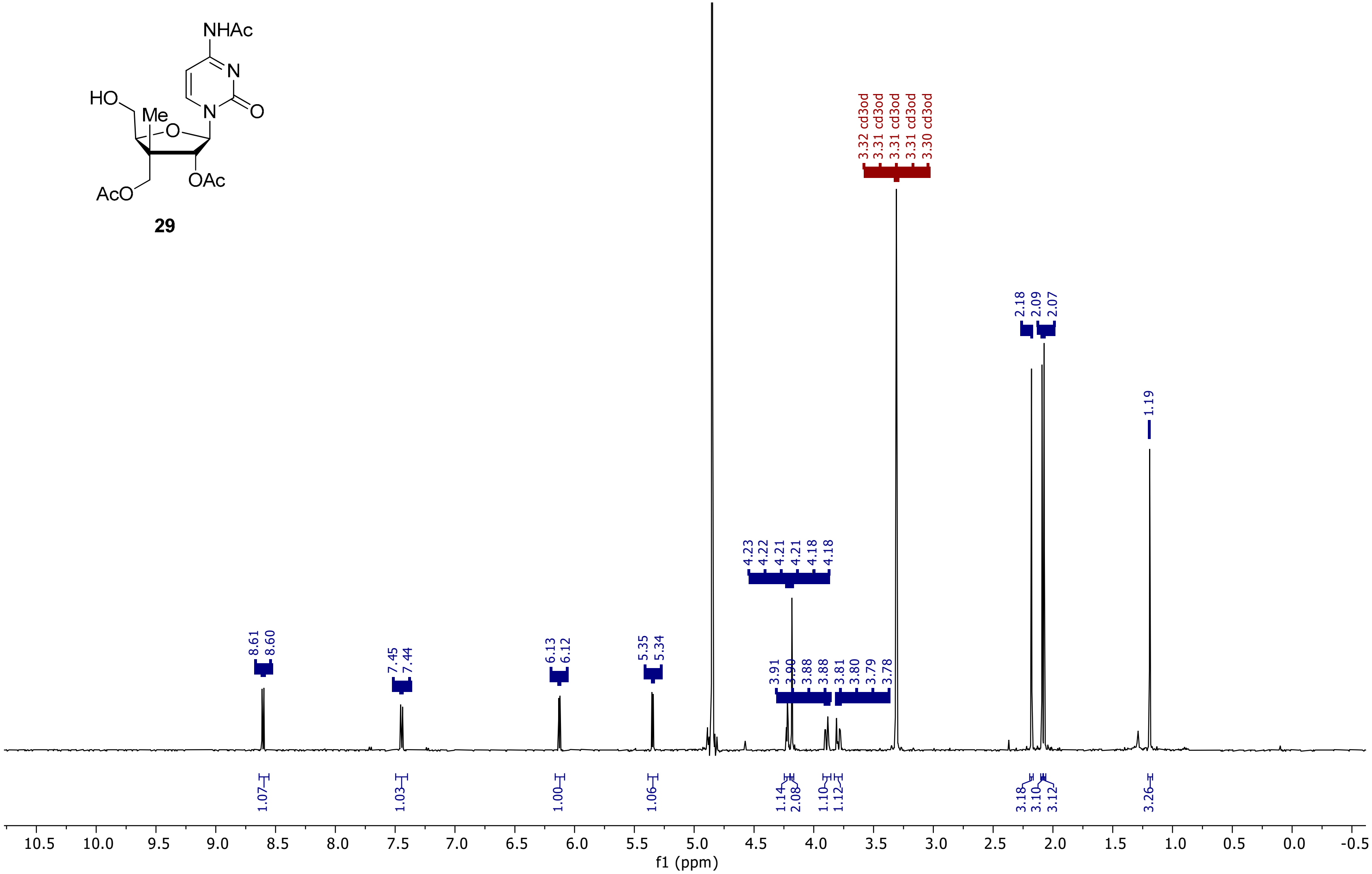

$^{13}C\{^{1}H\}$-NMR (126 MHz, CD_3OD)

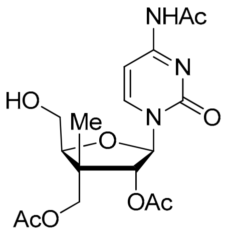

29

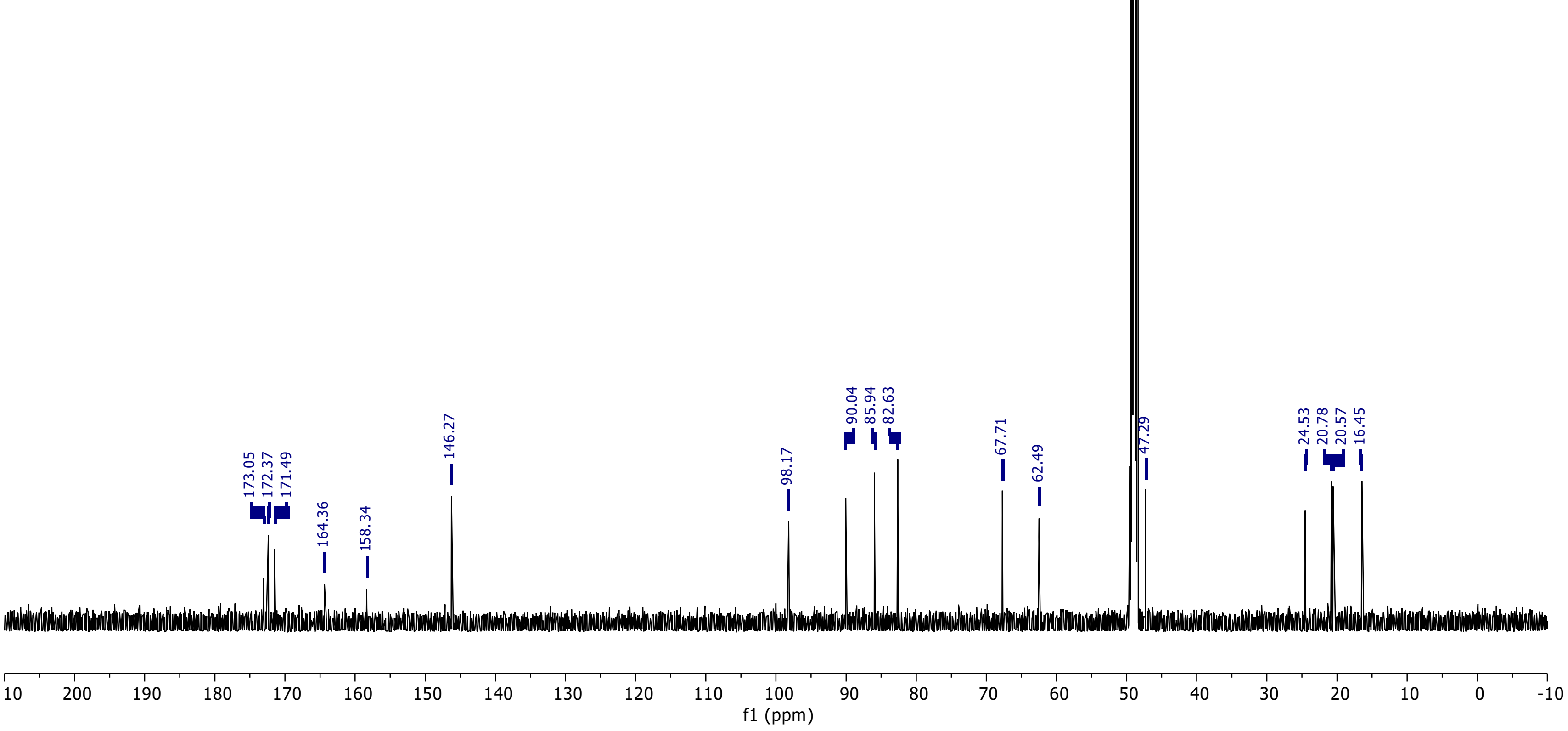

NOESY (500 MHz, CD_3OD)

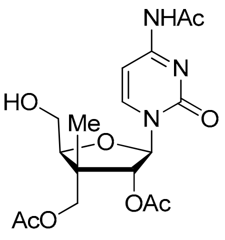

29

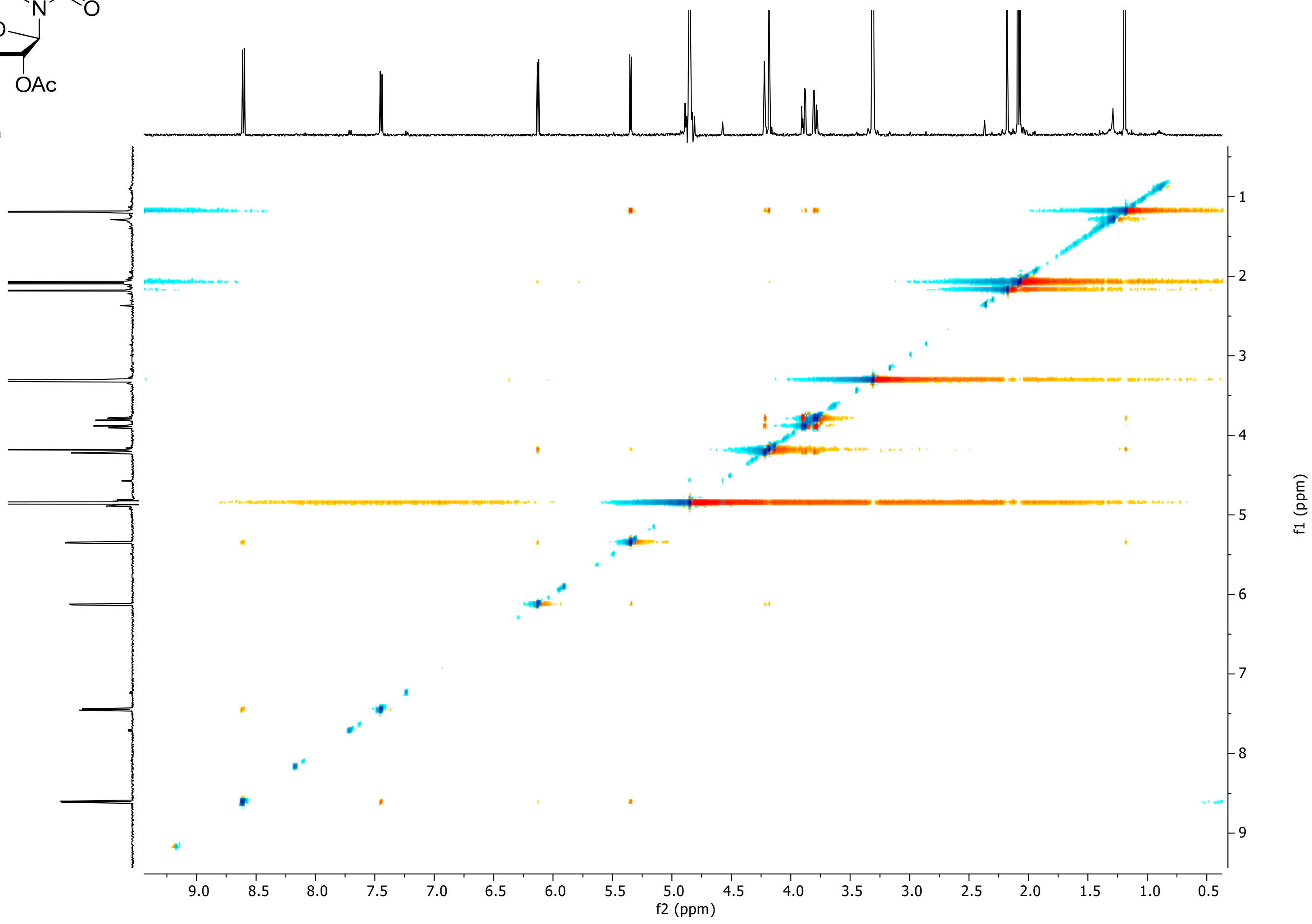

^{1}H-NMR (500 MHz, CD_3OD)

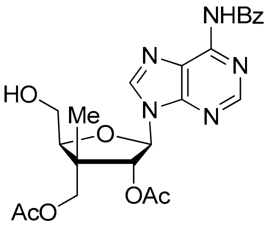

30

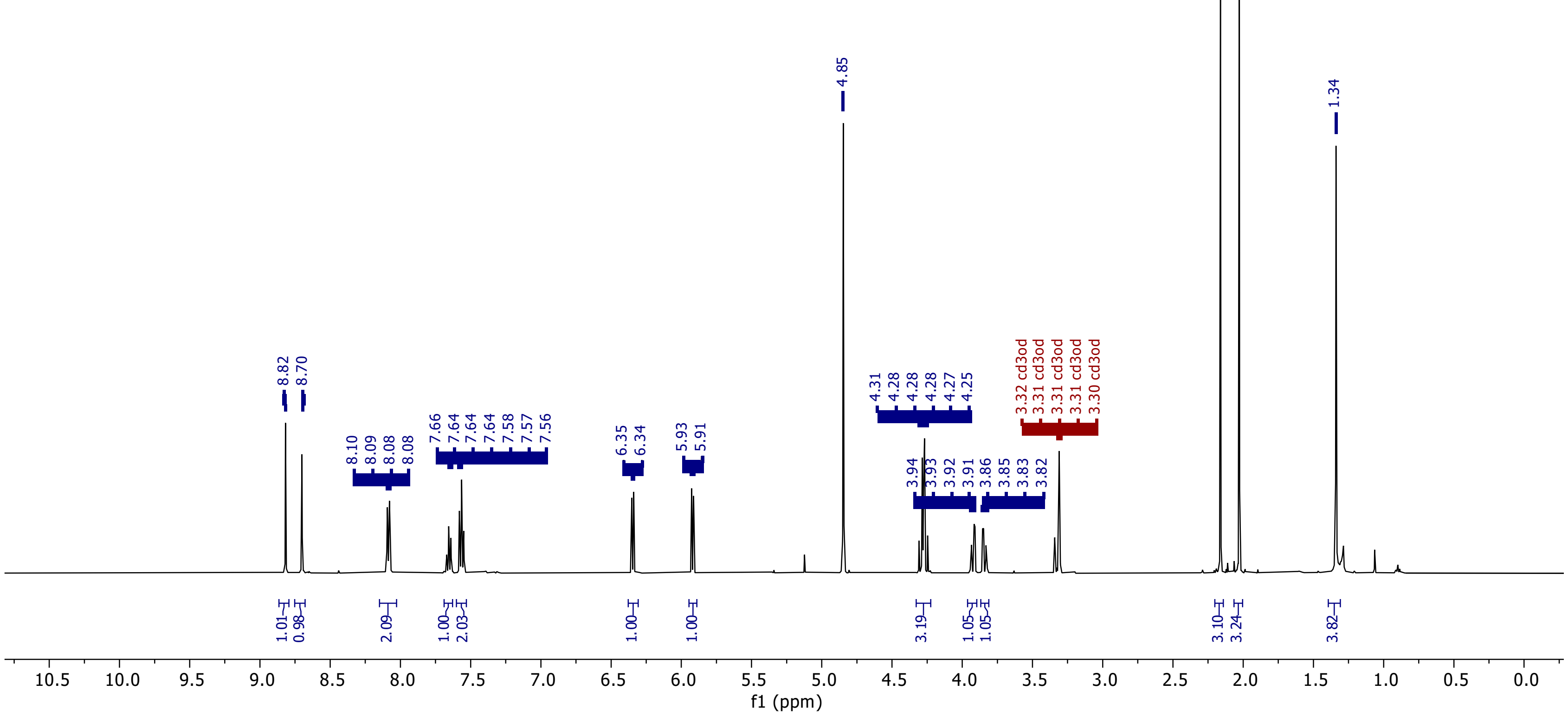

$^{13}C\{^{1}H\}$-NMR (126 MHz, CD_3OD)

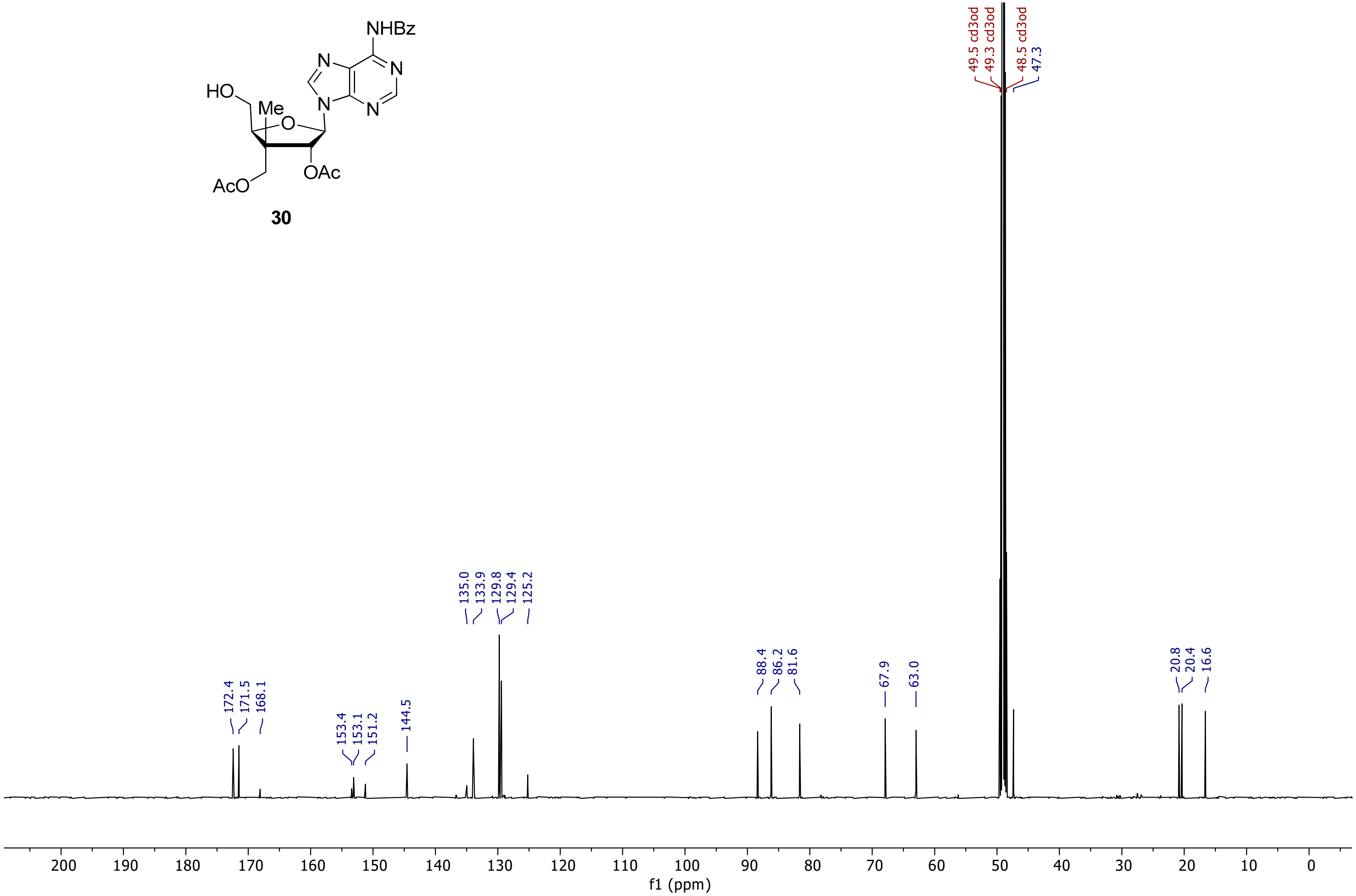

NOESY (500 MHz, CD_3OD)

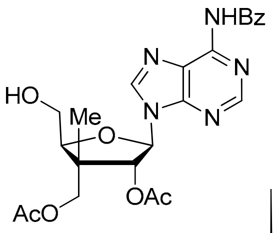

30

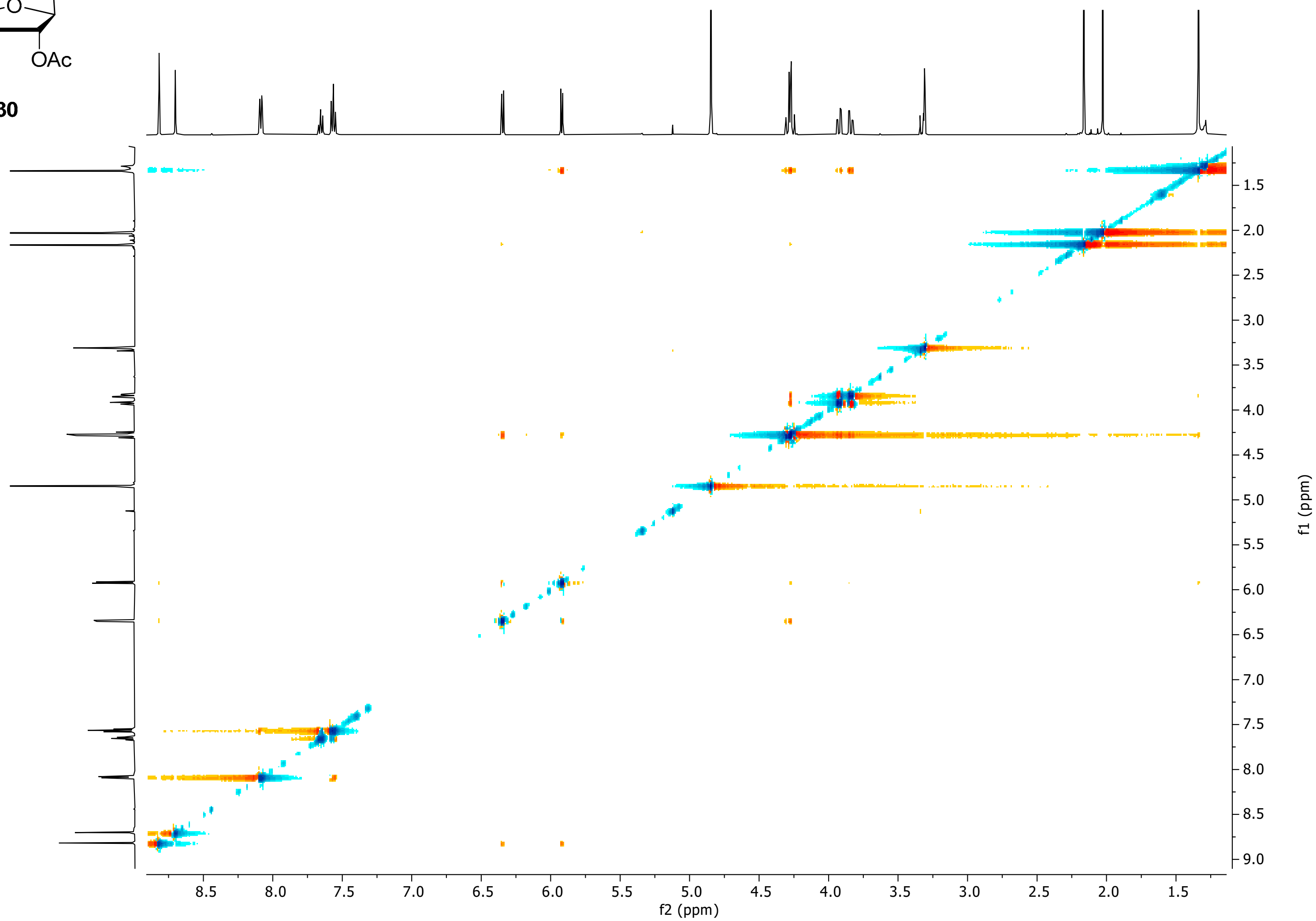

HMBC (500 MHz, CD_3OD)

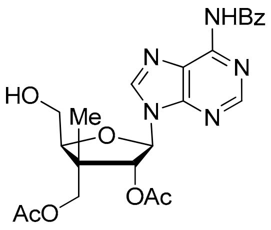

30

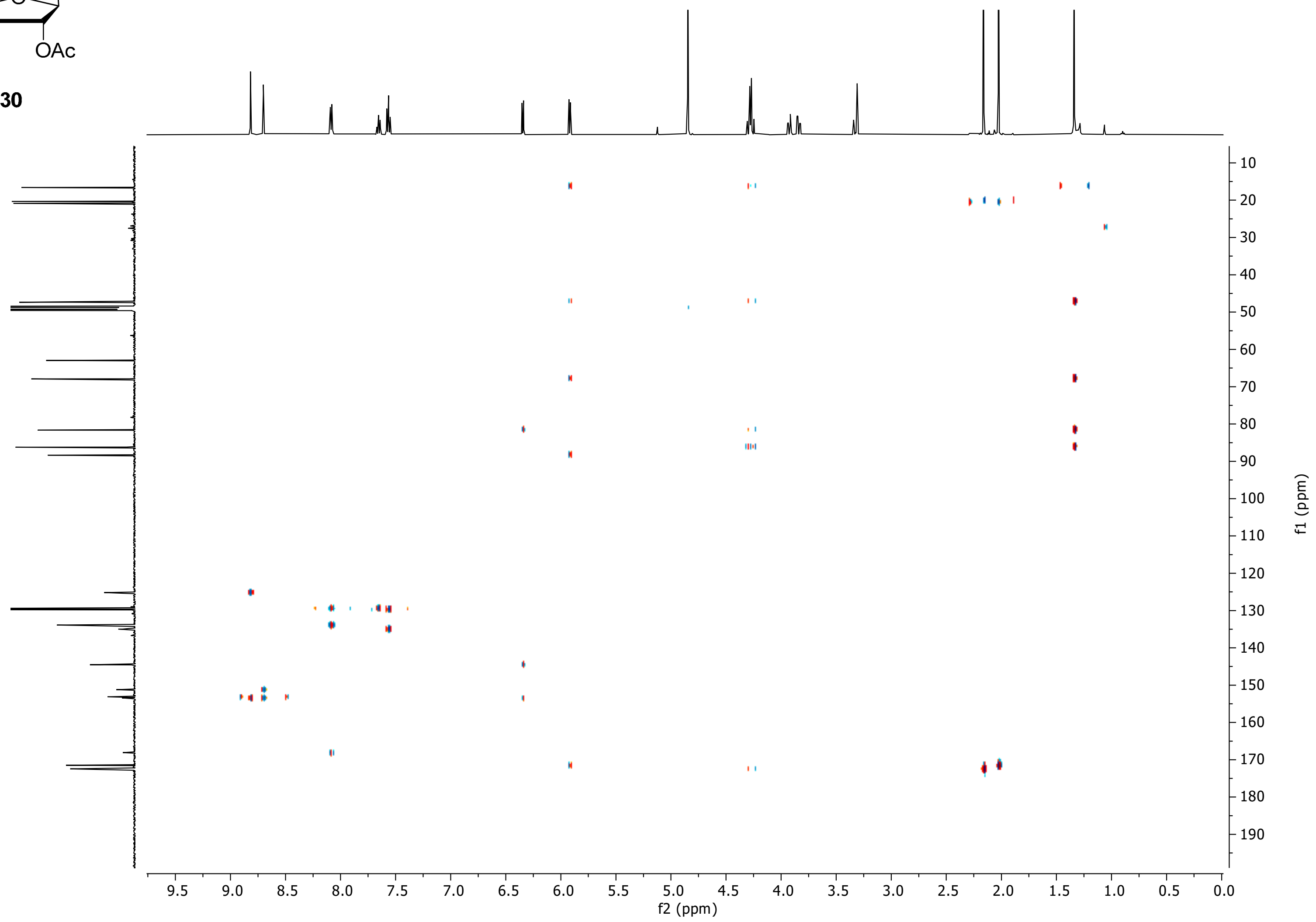

HSQC (500 MHz, CD_3OD)

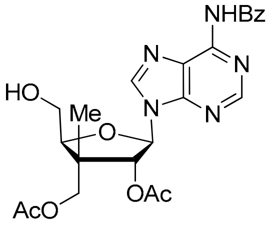

30

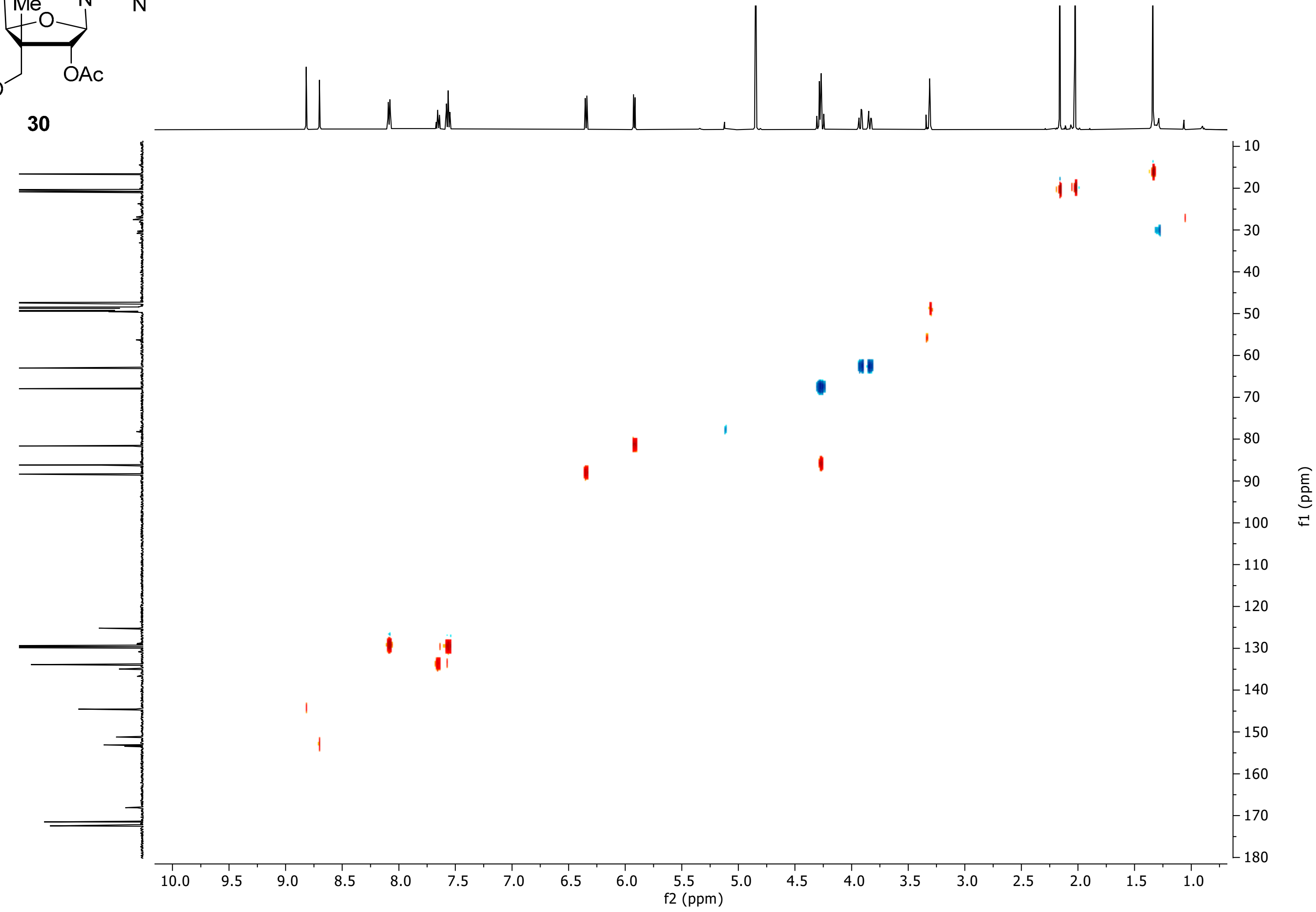

^{1}H-NMR (500 MHz, CD_3OD)

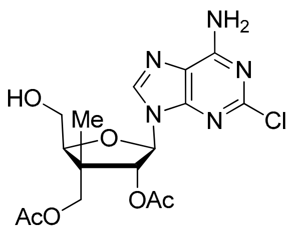

31

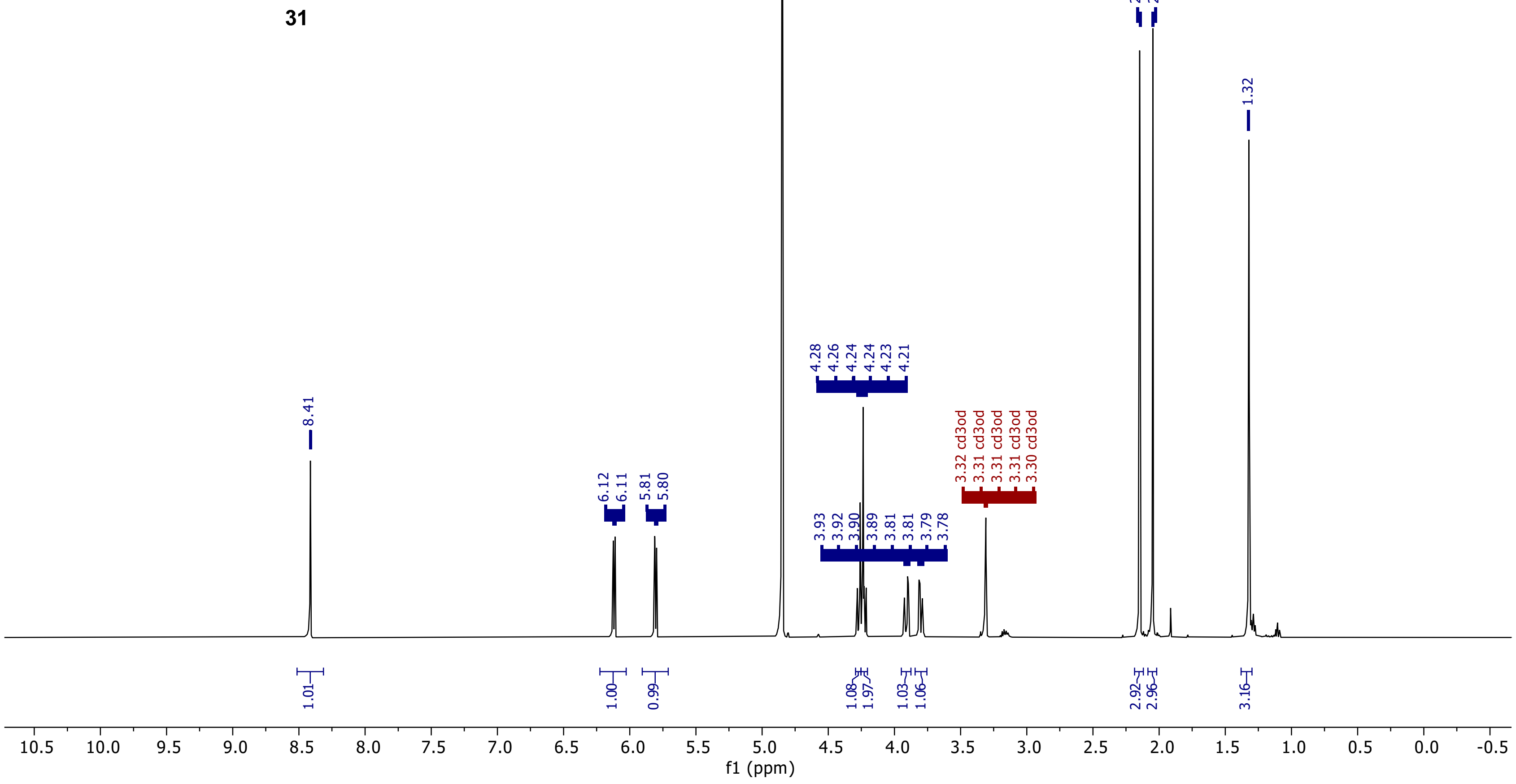

$^{13}C\{^{1}H\}$-NMR (126 MHz, CD_3OD)

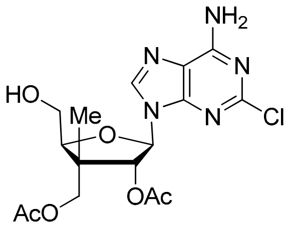

31

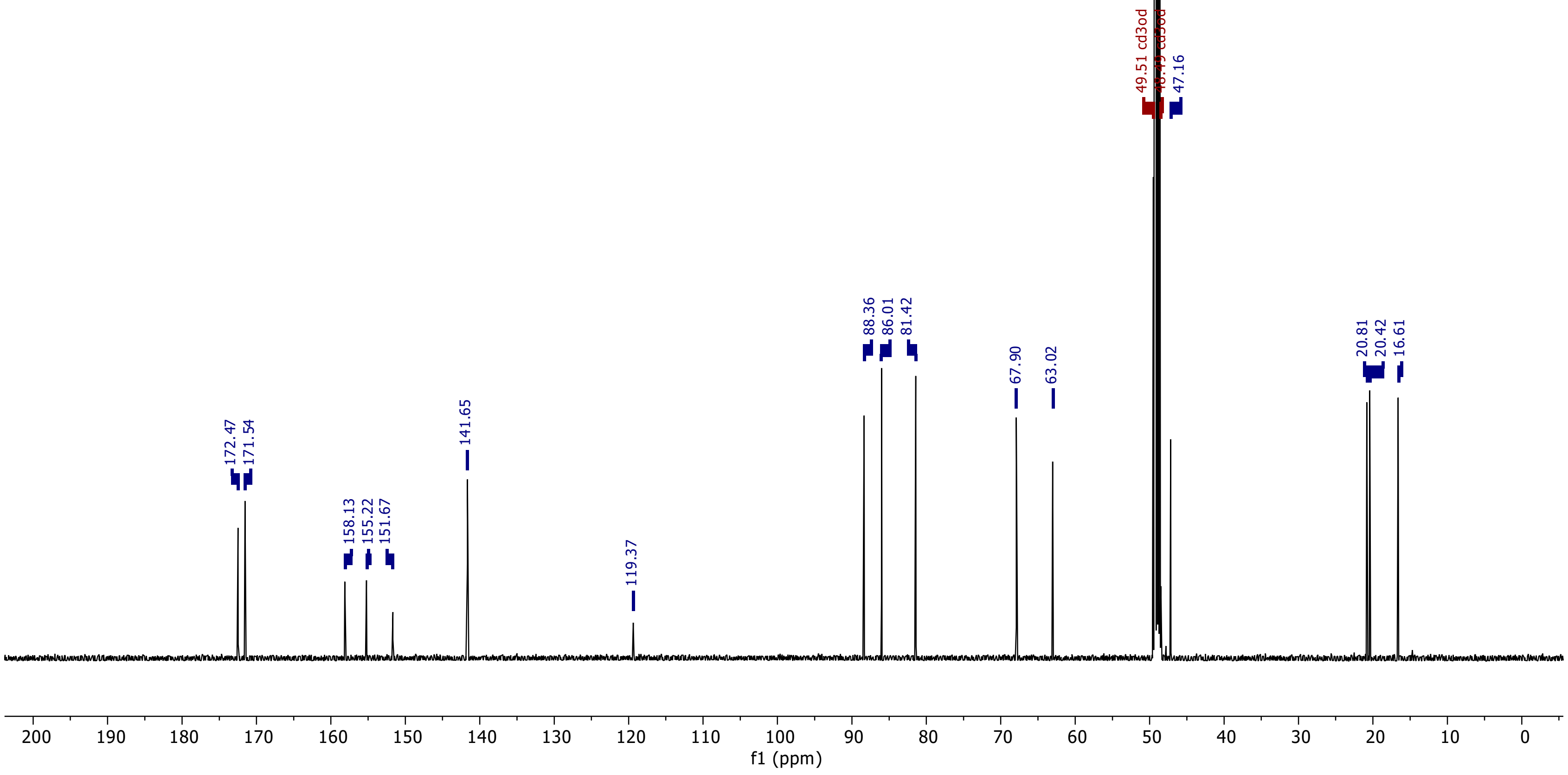

NOESY (500 MHz, CD_3OD)

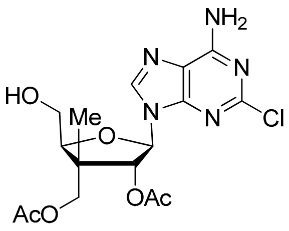

31

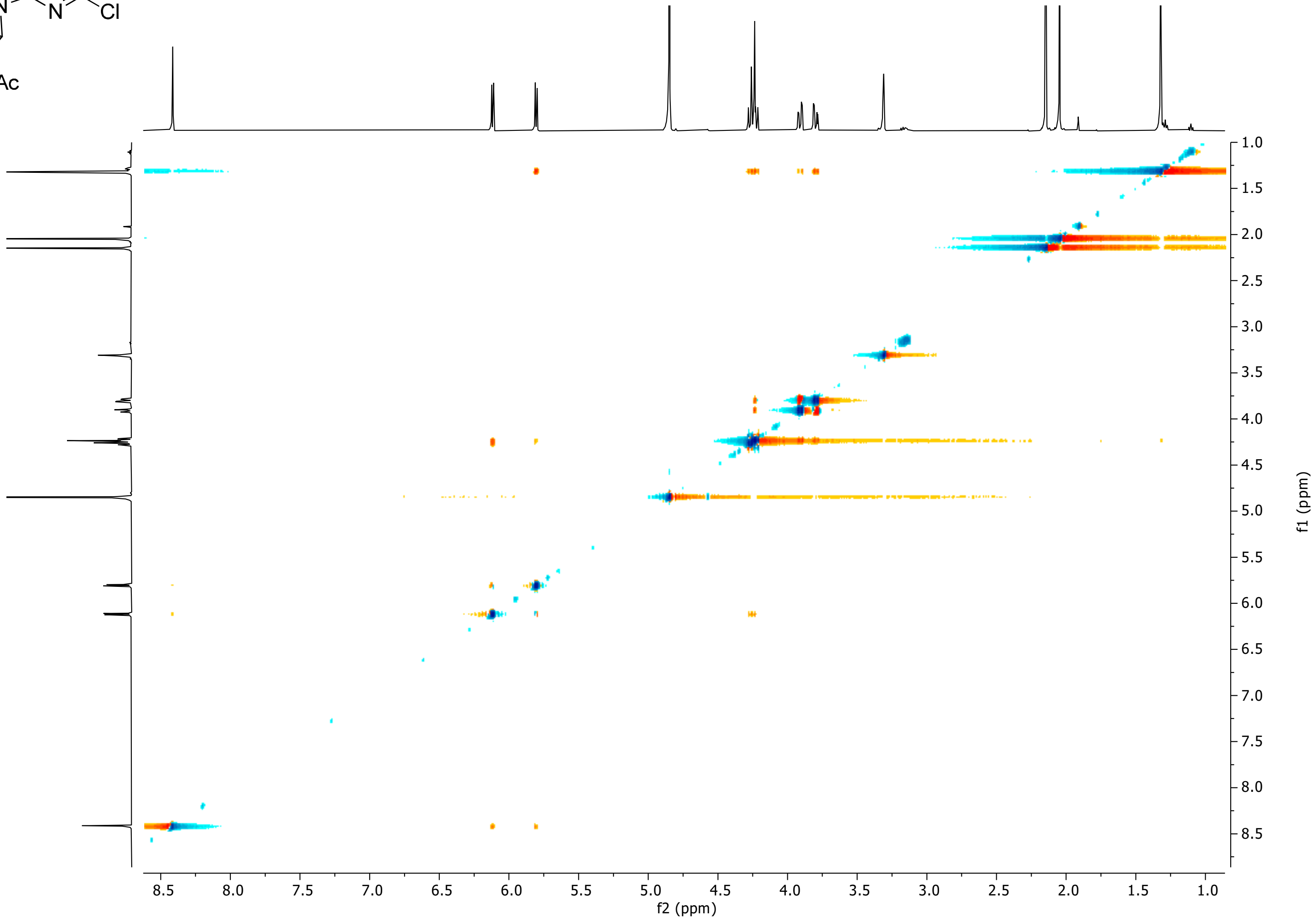

HMBC (500 MHz, CD_3OD)

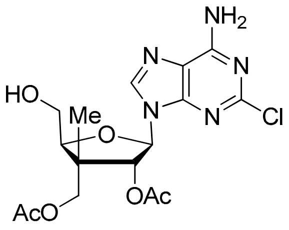

31

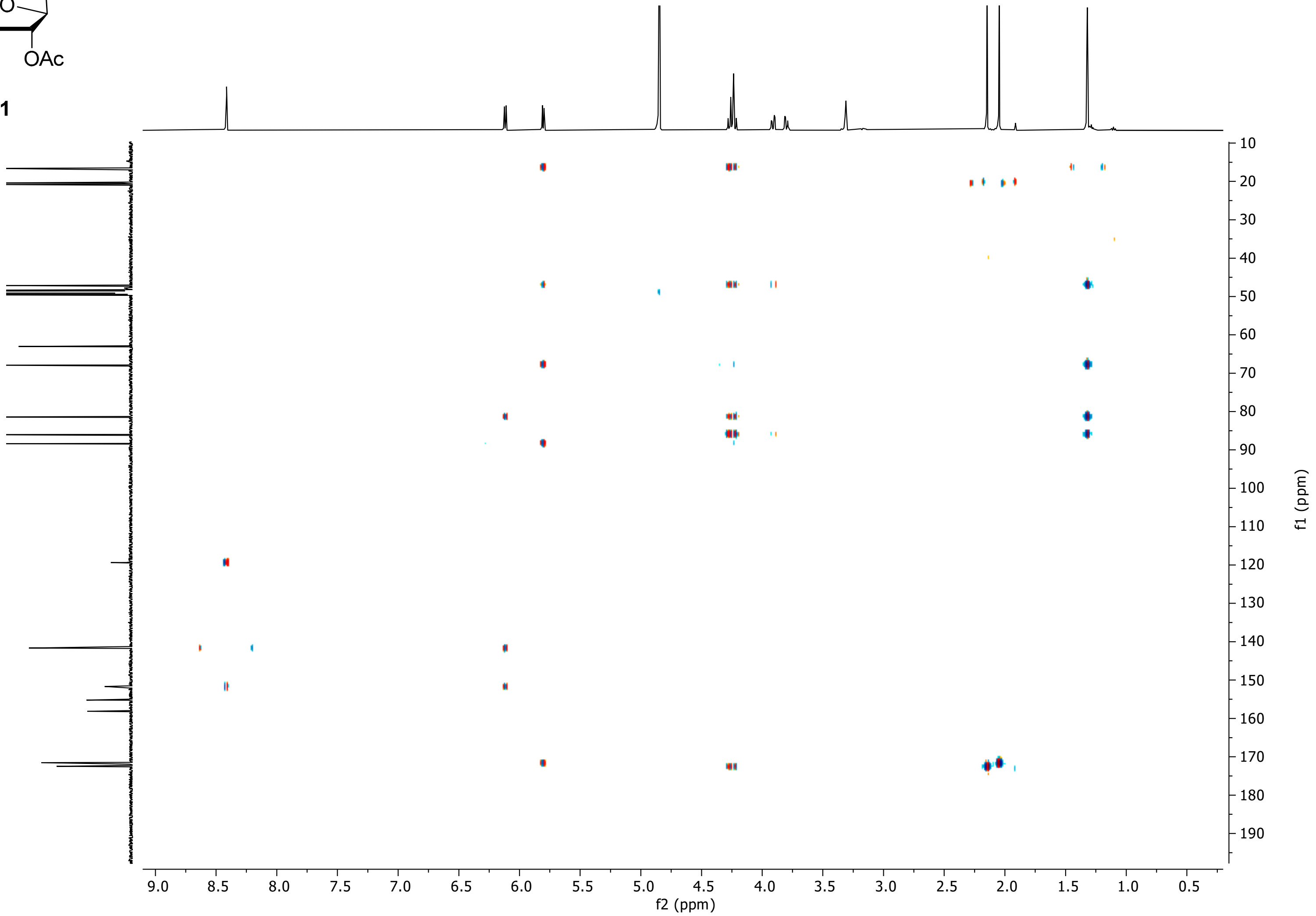

HSQC (500 MHz, CD_3OD)

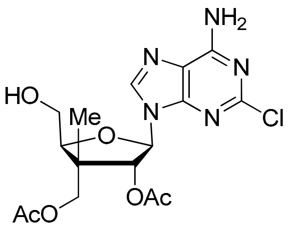

31

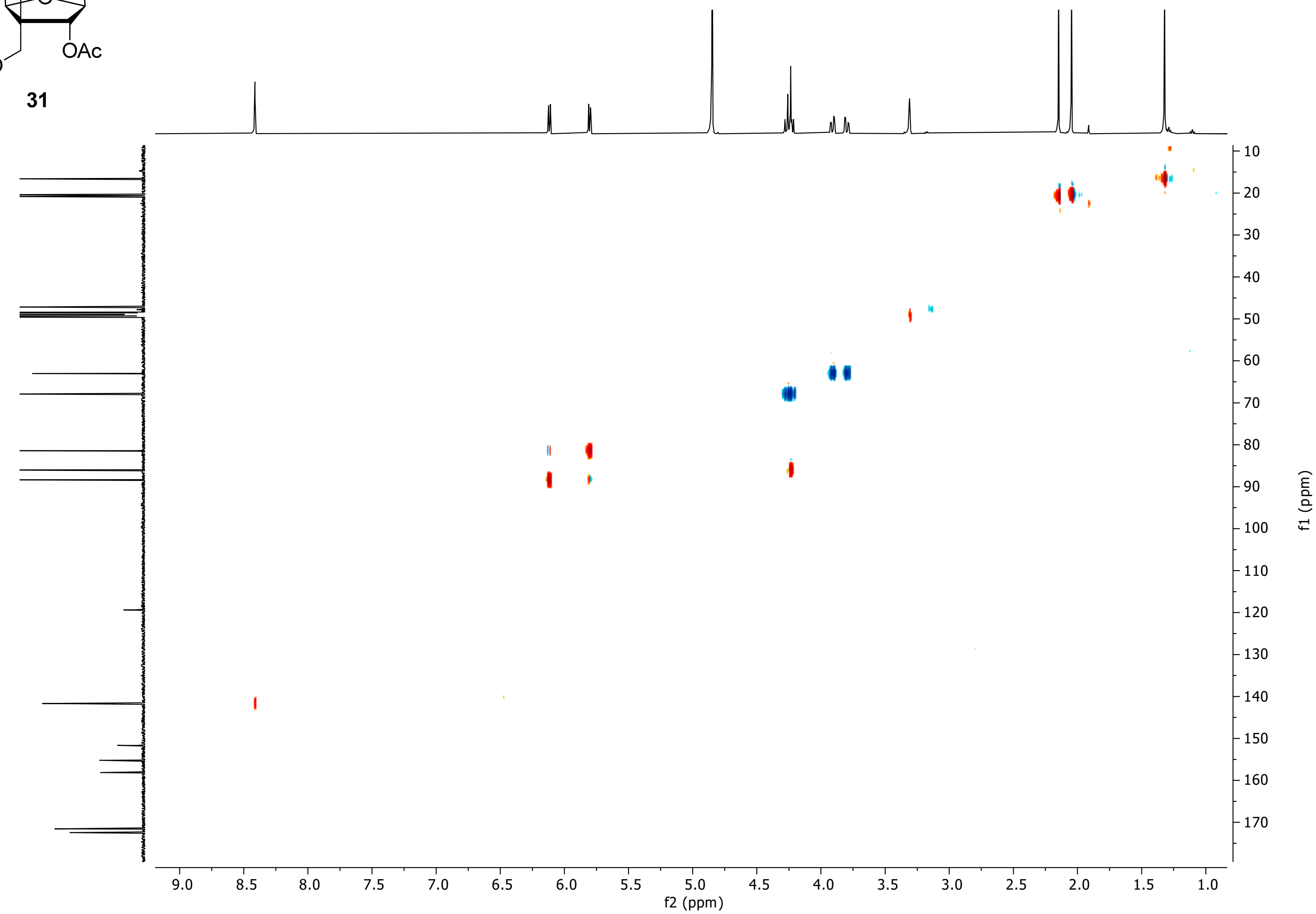

^{1}H-NMR (500 MHz, CD_3OD)

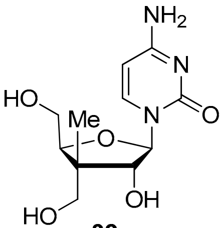

32

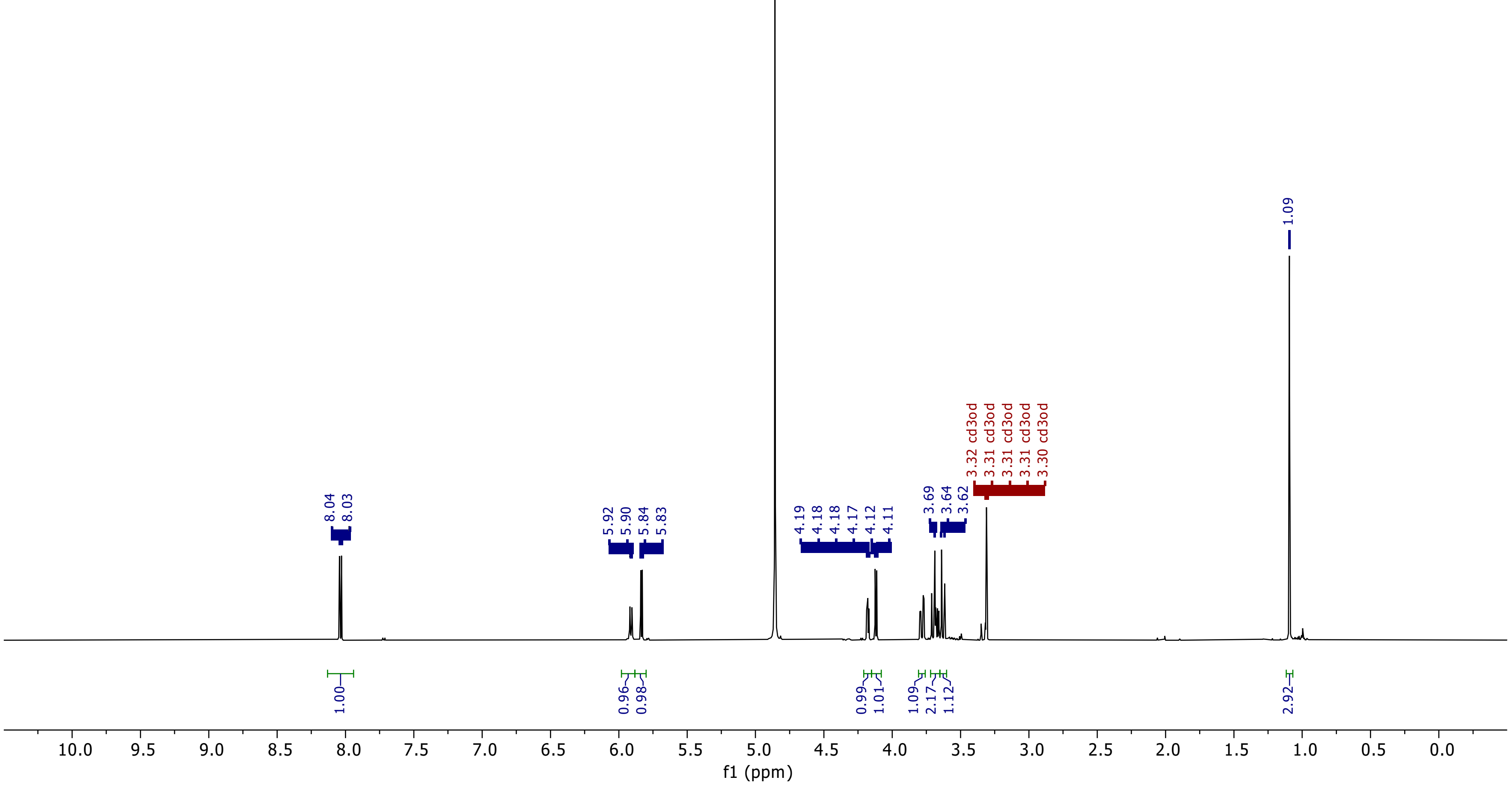

$^{13}C\{^{1}H\}$-NMR (126 MHz, CD_3OD)

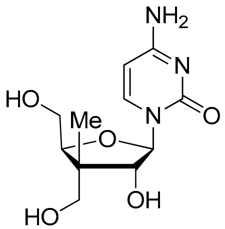

32

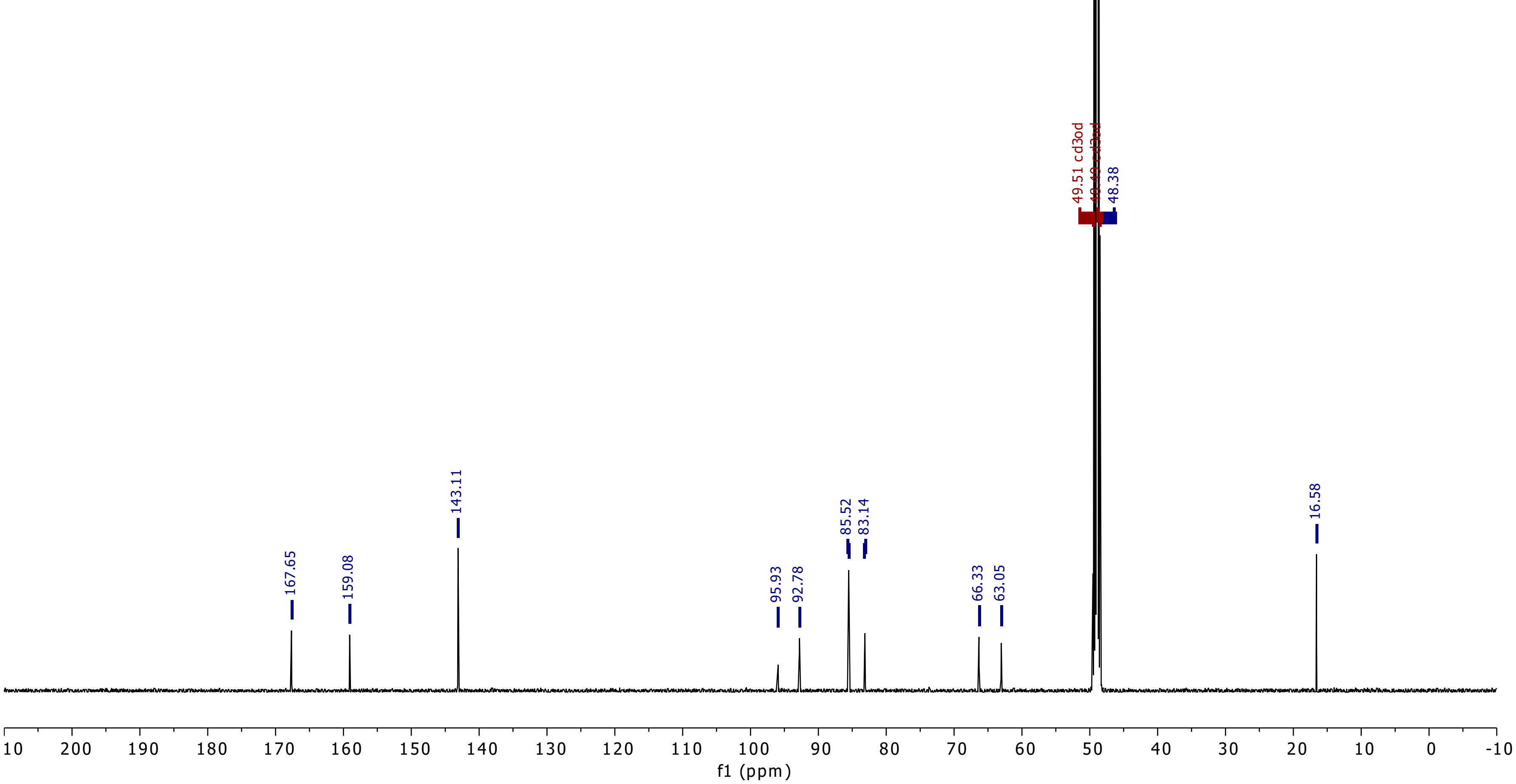

^{1}H-NMR (500 MHz, CD_3OD)

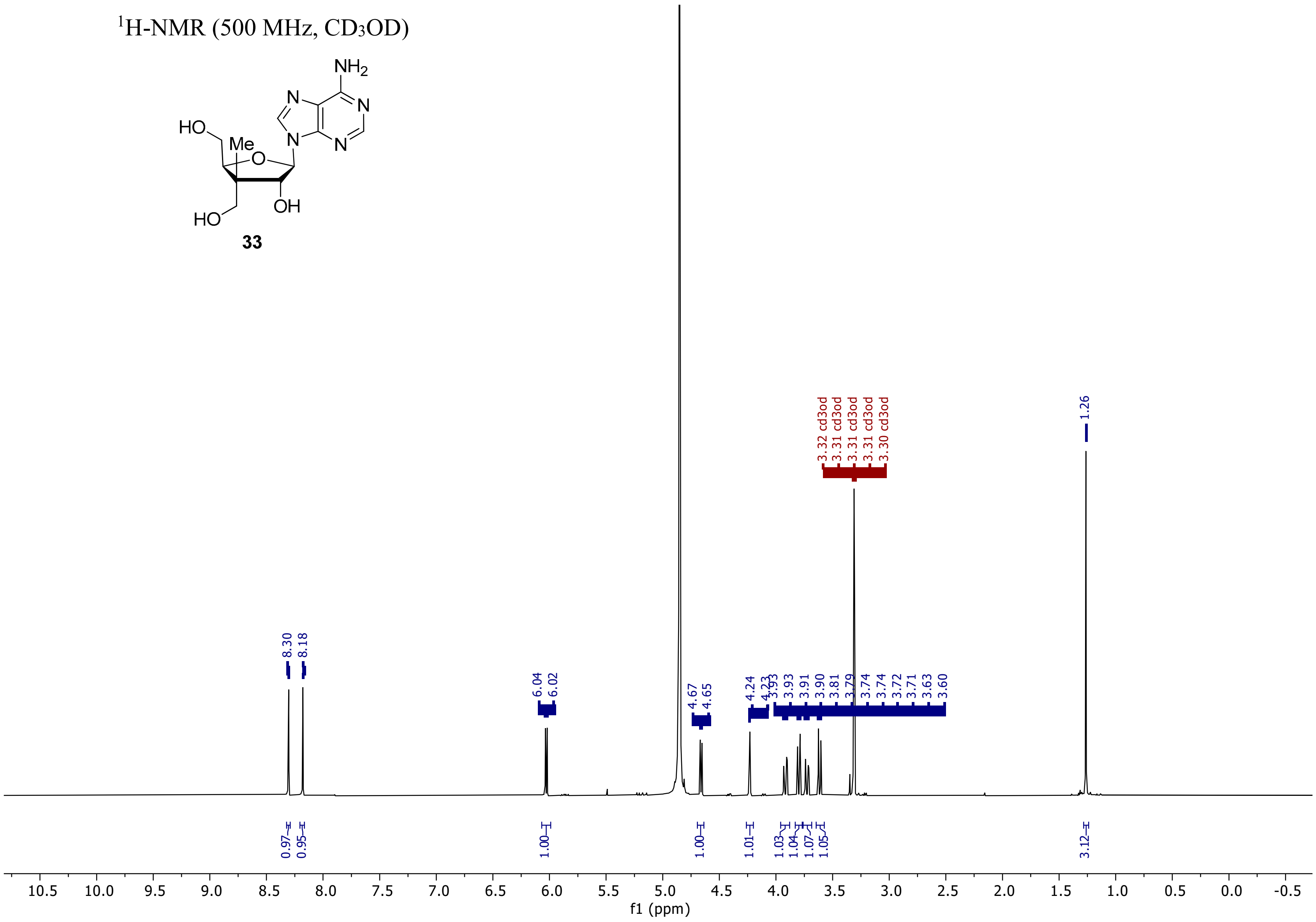

$^{13}C\{^{1}H\}$-NMR (126 MHz, CD_3OD)

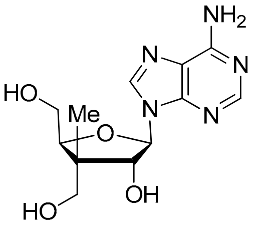

33

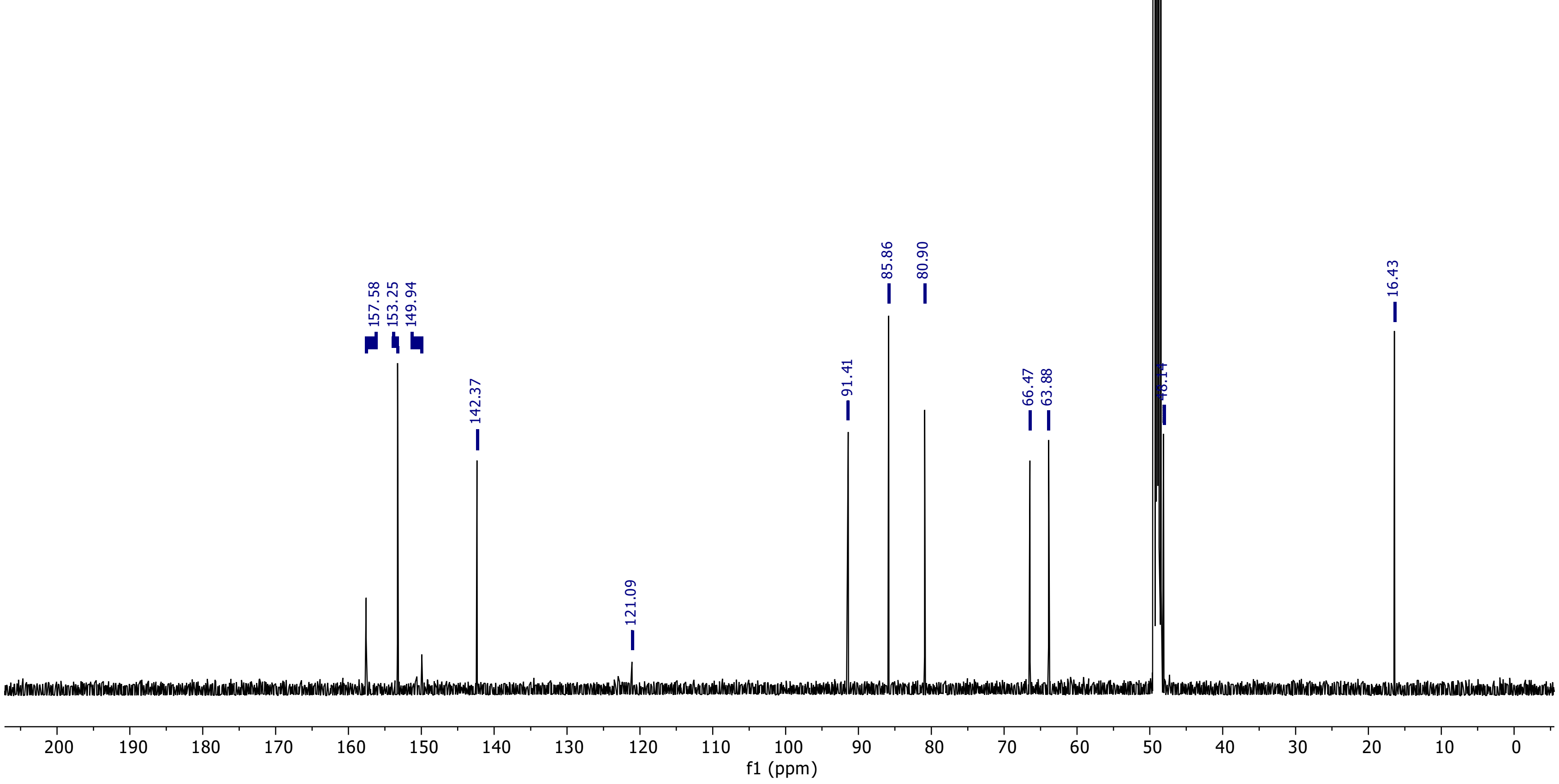

NOESY (500 MHz, CD_3OD)

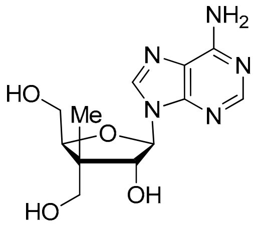

33

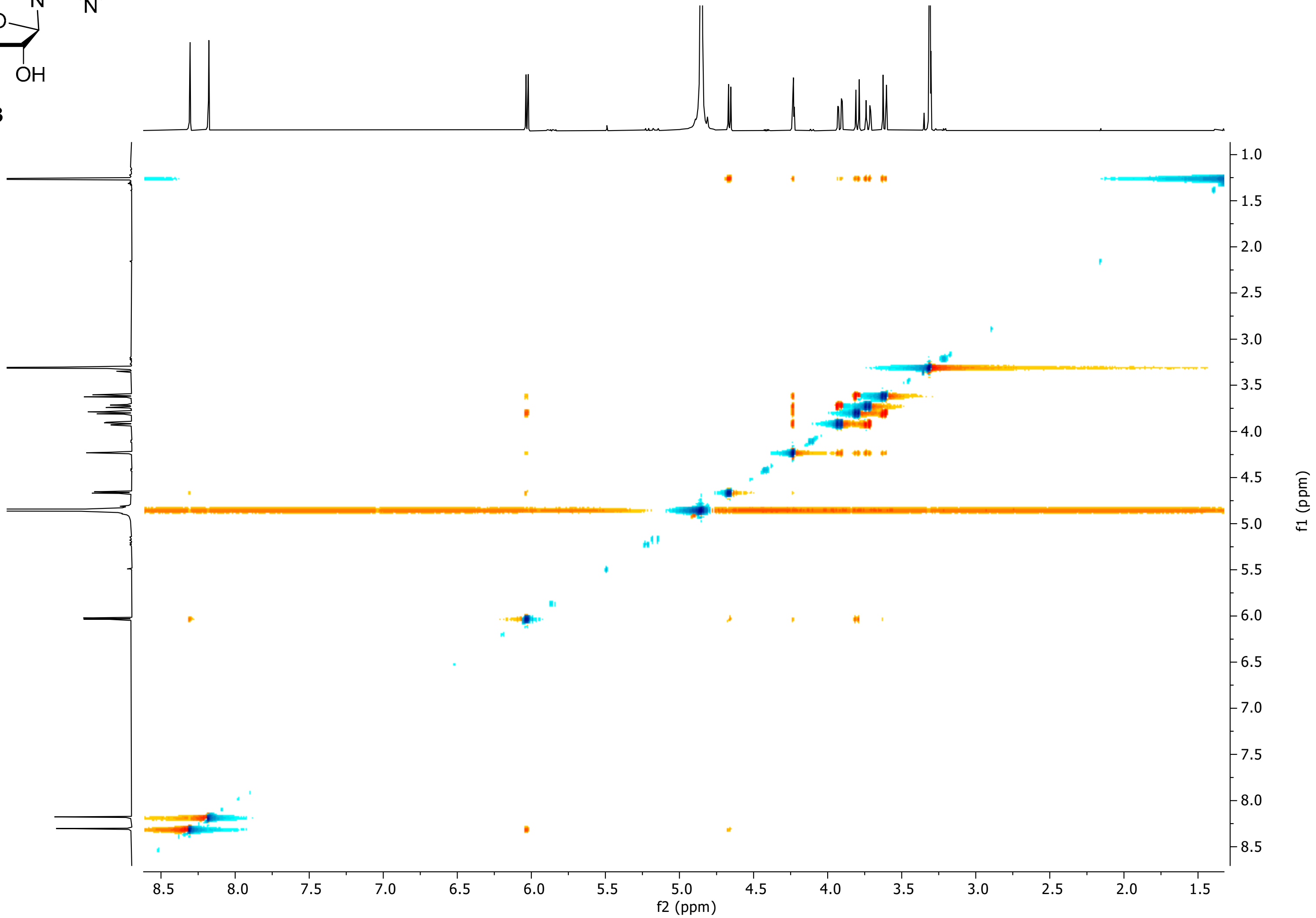

HMBC (500 MHz, CD_3OD)

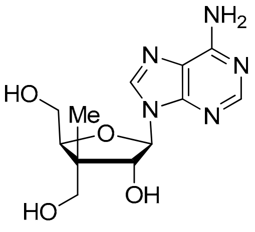

33

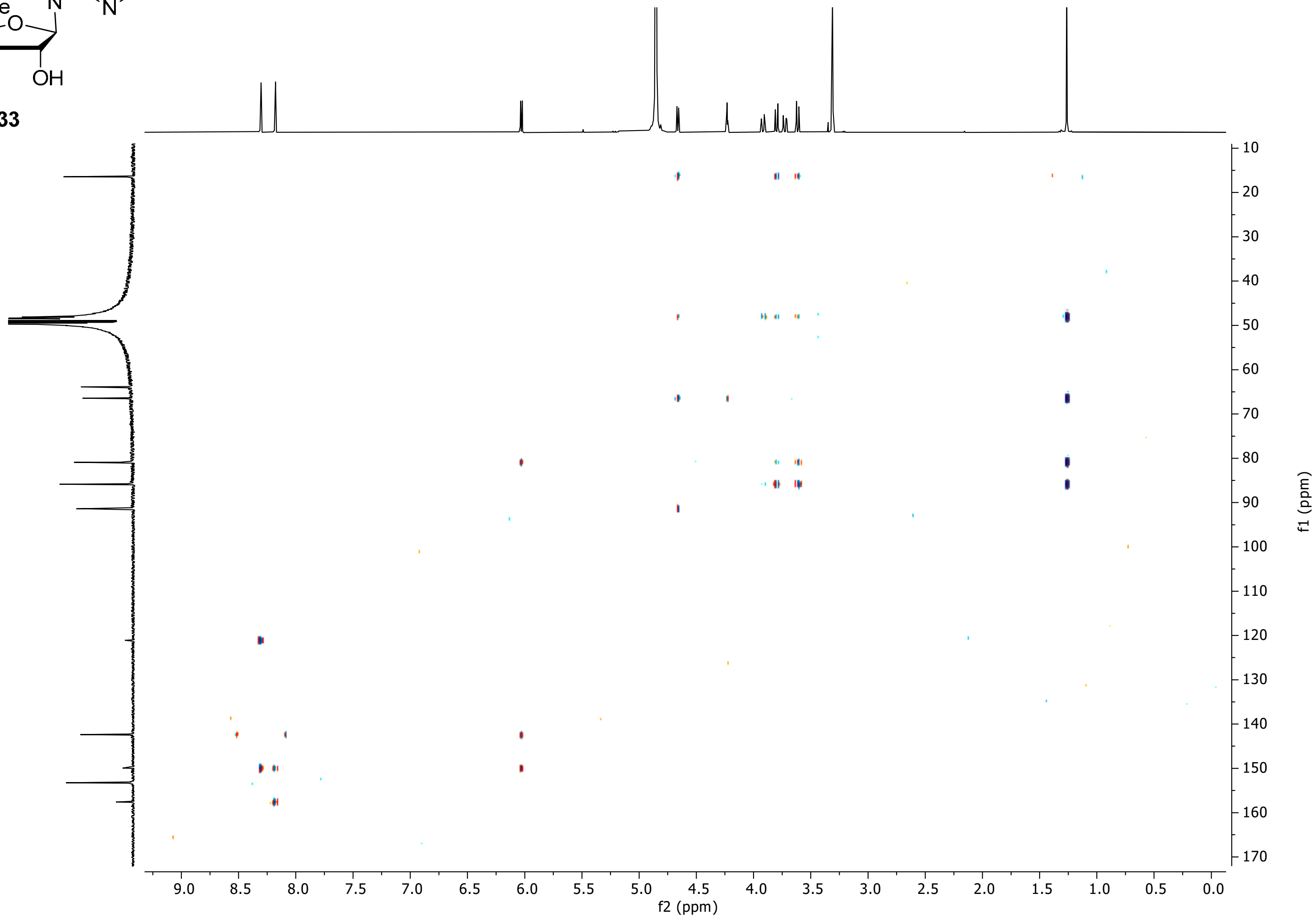

HSQC (500 MHz, CD_3OD)

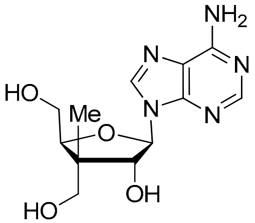

33

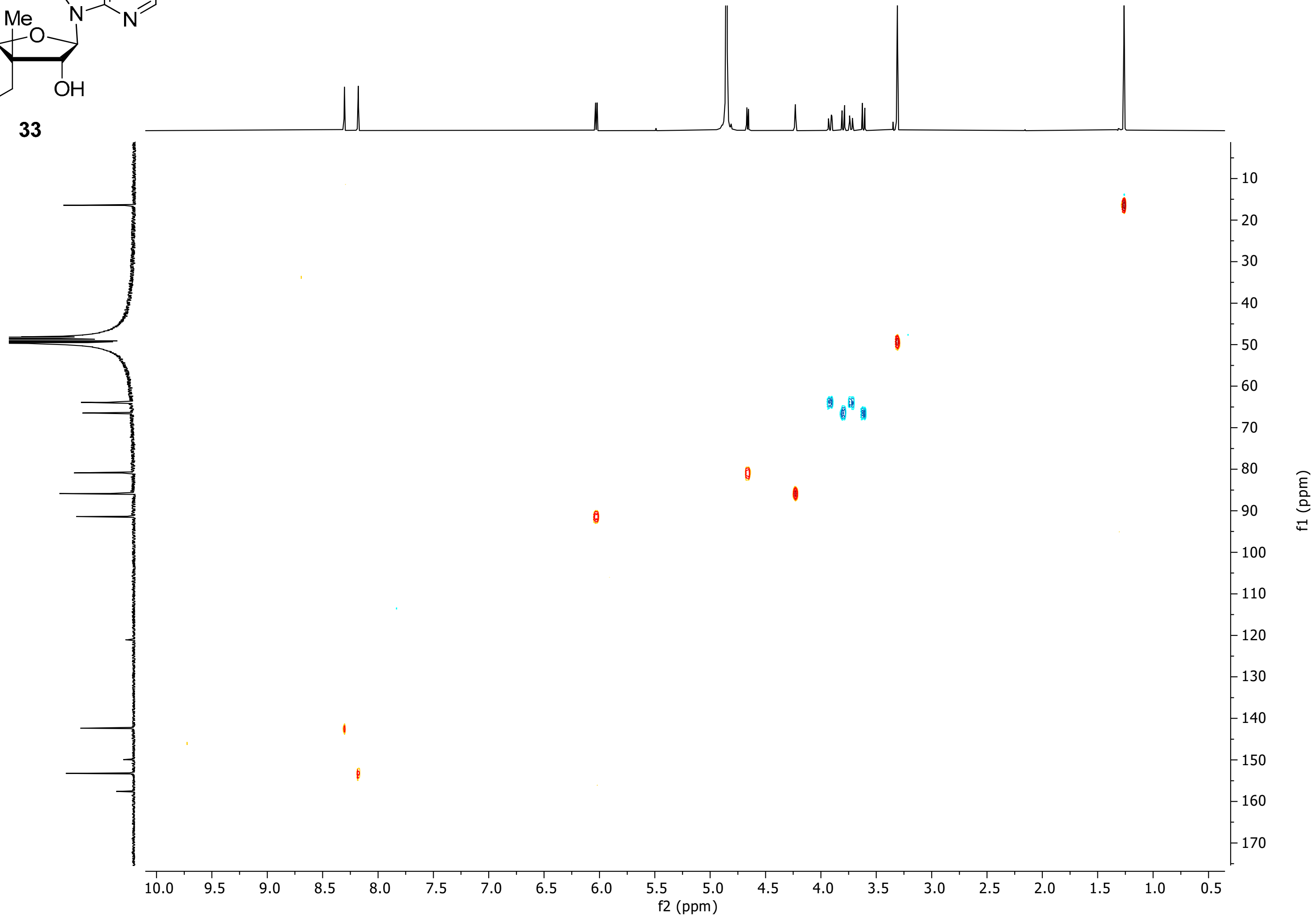

^{1}H-NMR (500 MHz, CD_3OD)

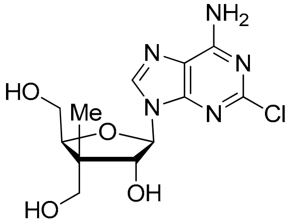

34

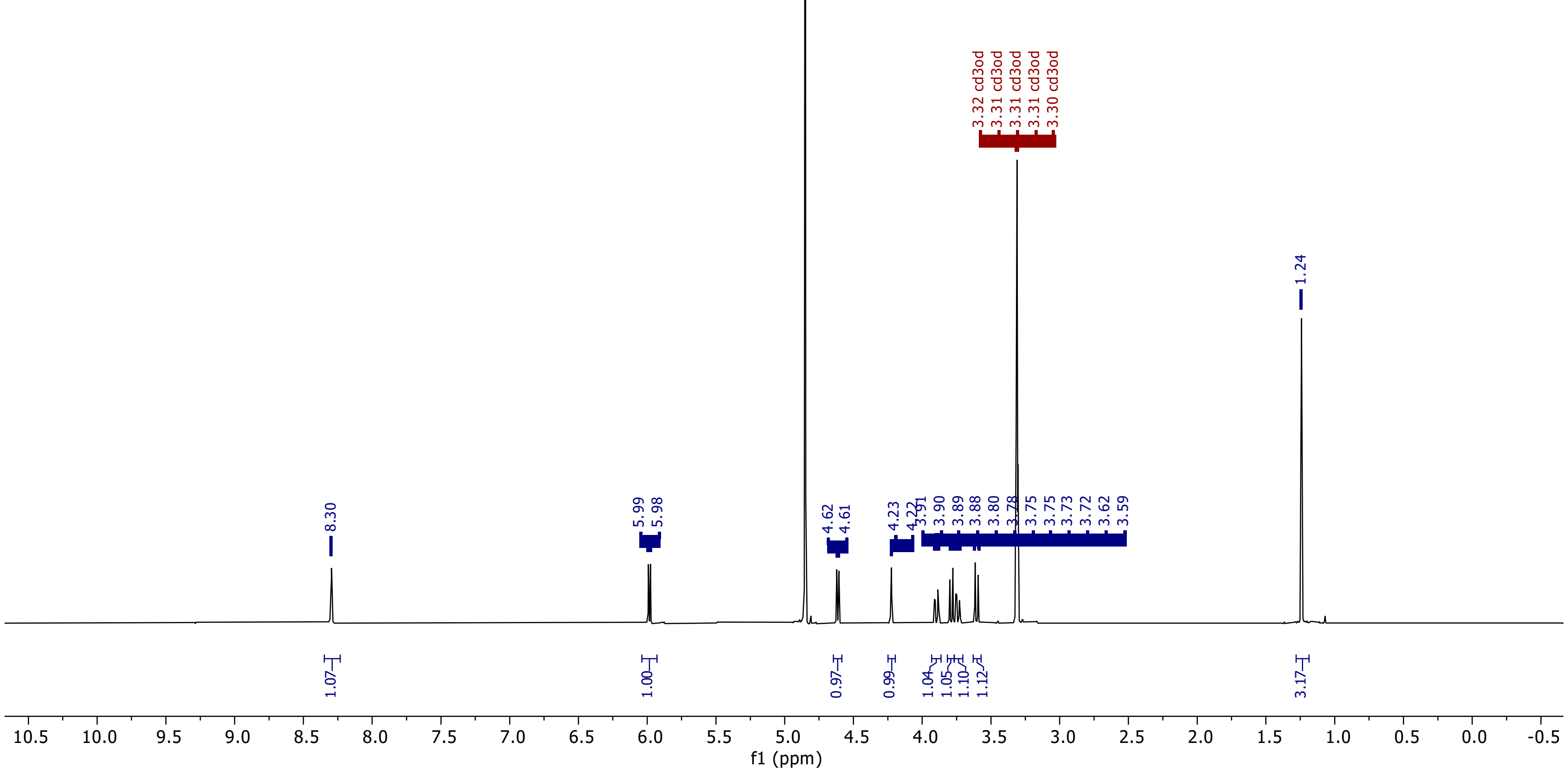

$^{13}C\{^{1}H\}$-NMR (126 MHz, CD_3OD)

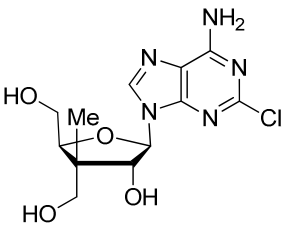

34

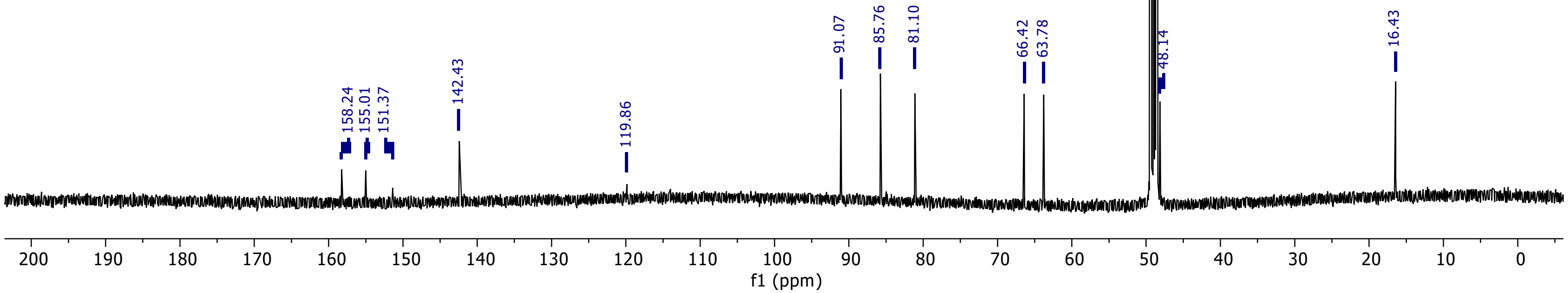

NOESY (500 MHz, CD_3OD)

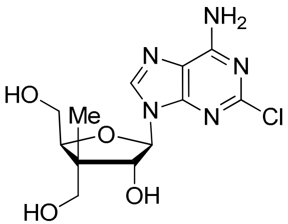

34

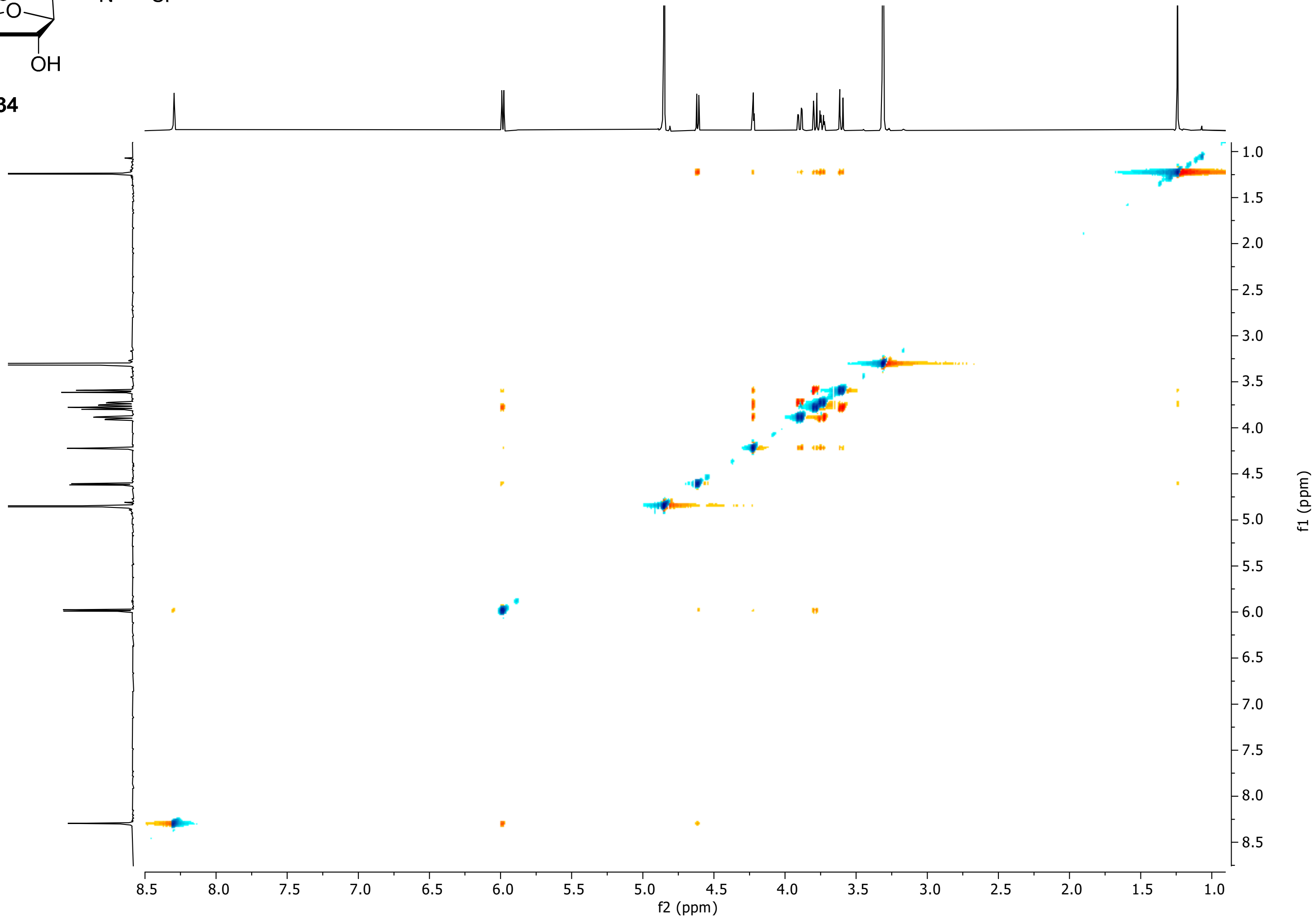

HMBC (500 MHz, CD_3OD)

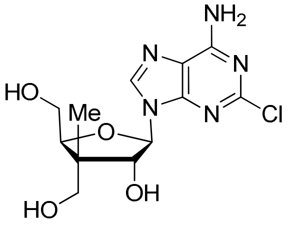

34

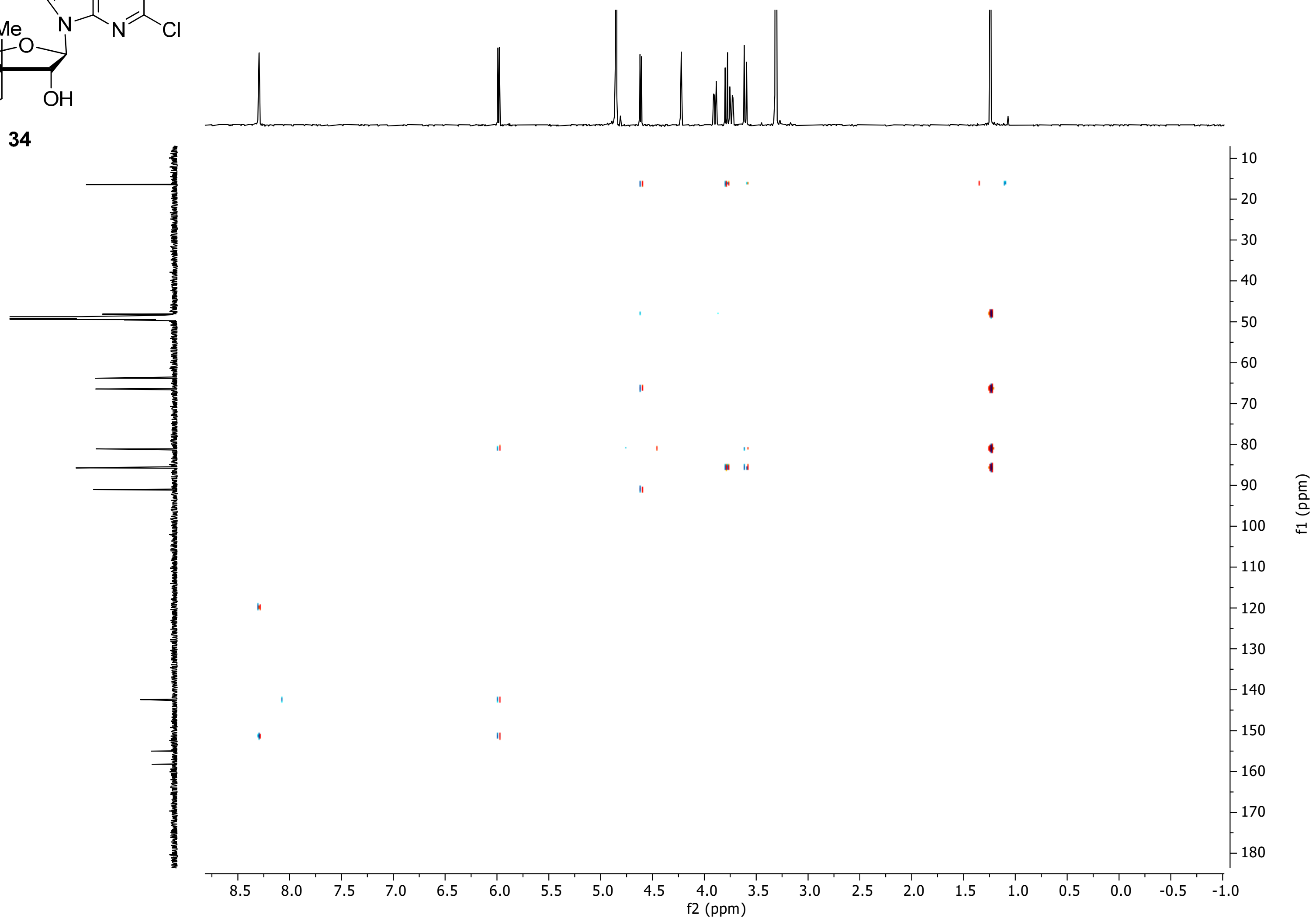

HSQC (500 MHz, CD3OD)

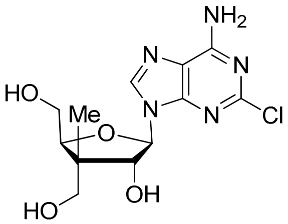

34

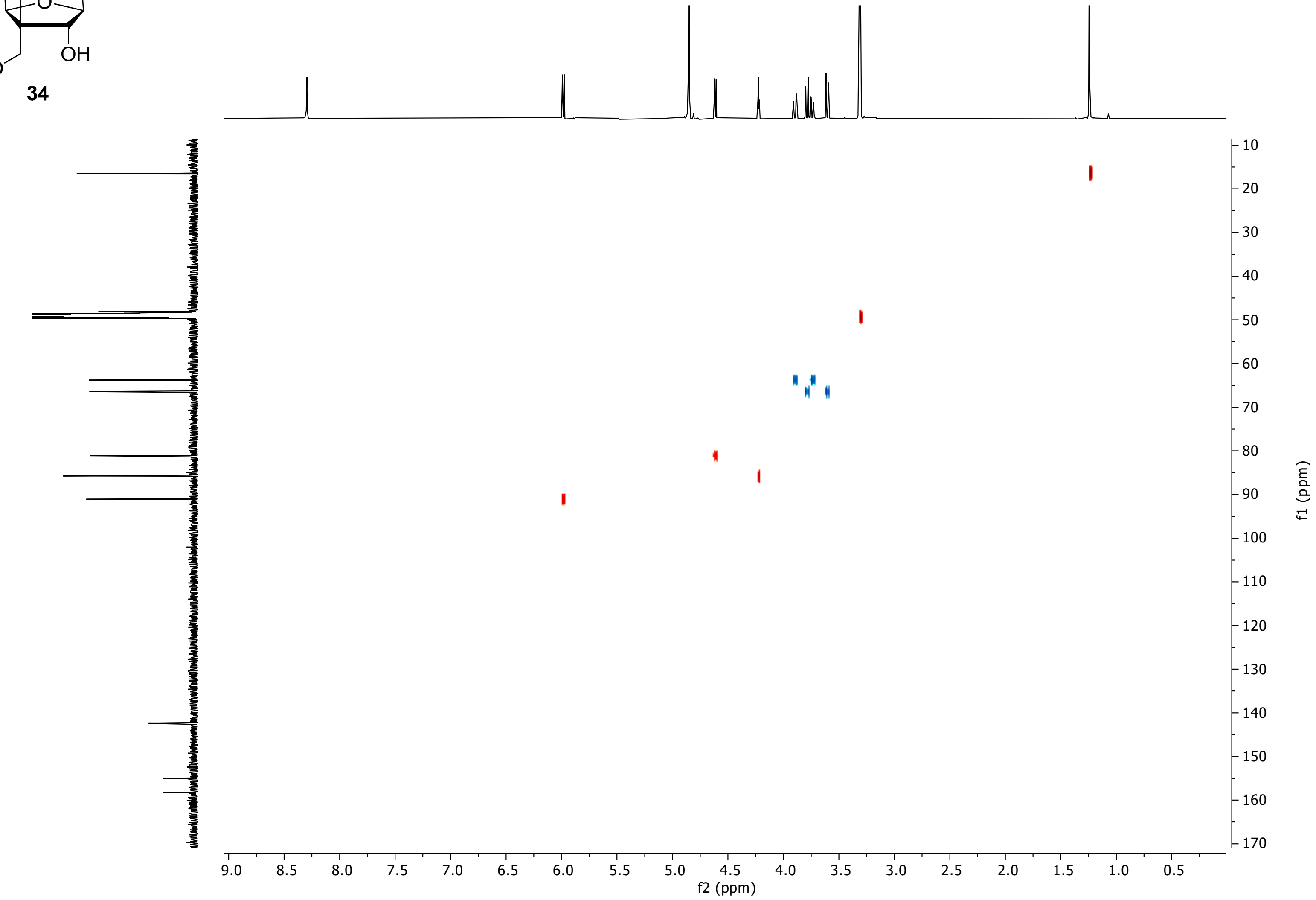

AM-ESP-1007

^{1}H-NMR (500 MHz, $CDCl_3$)

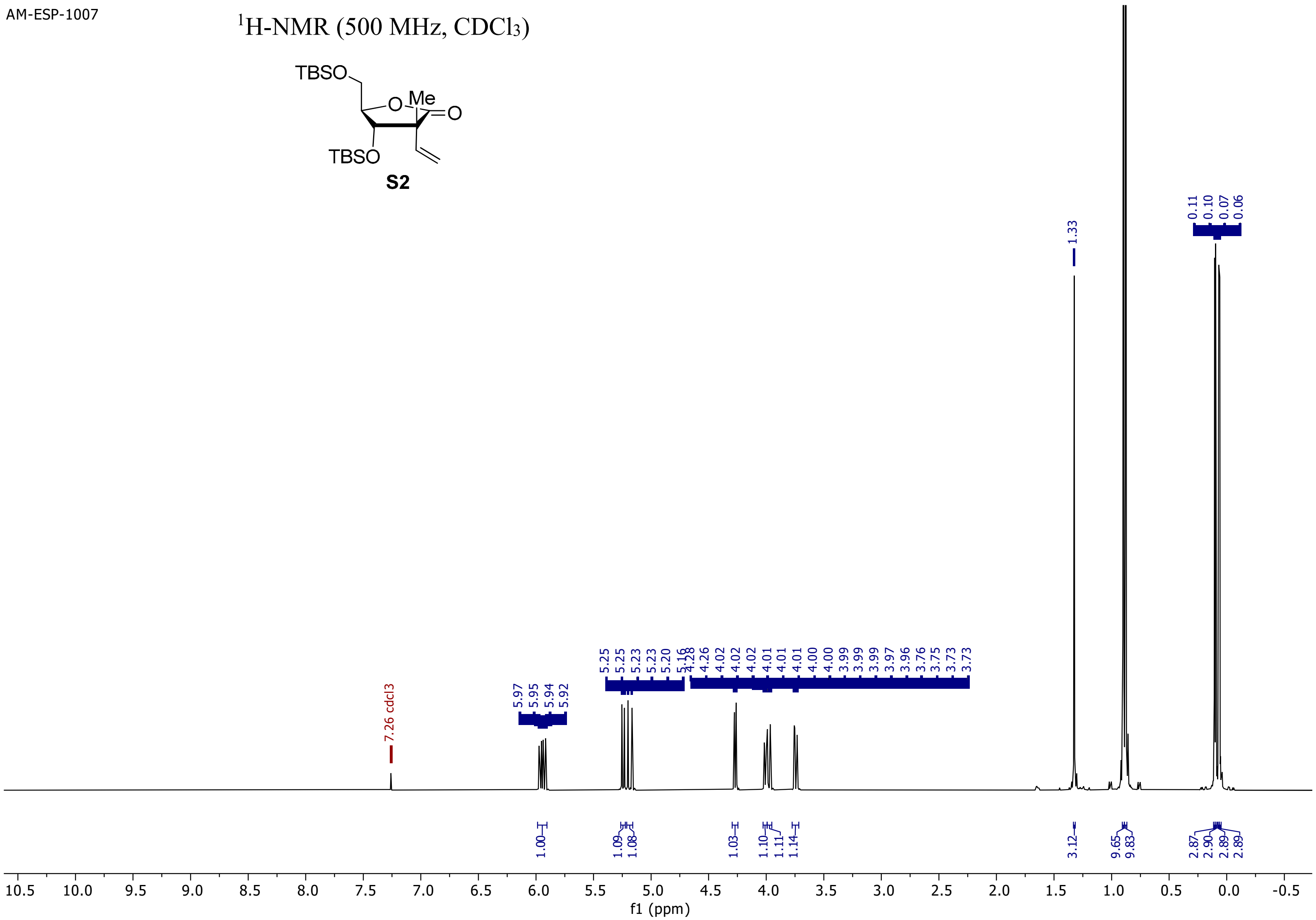

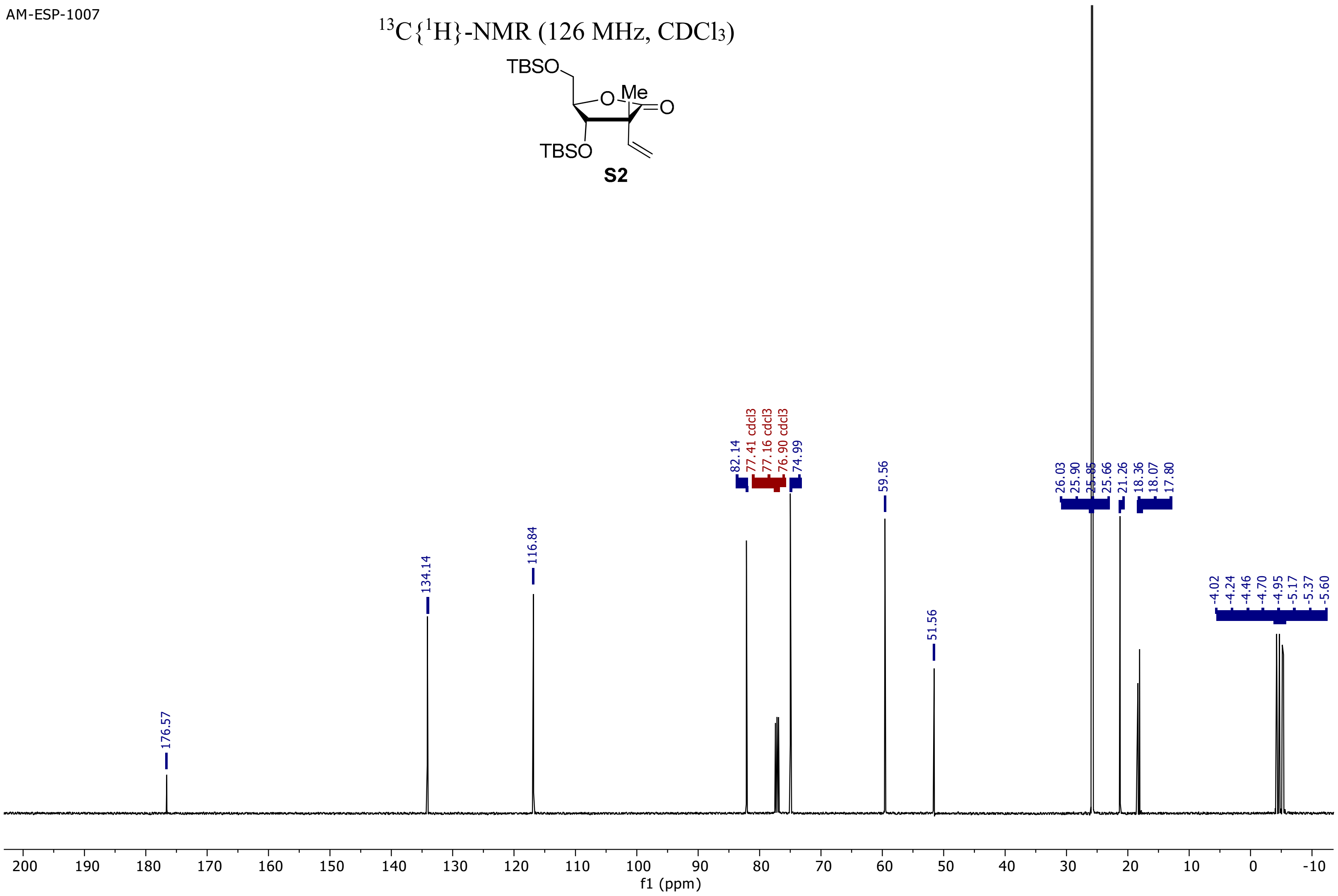
AM-ESP-1007
13C{1H}-NMR (126 MHz, CDCl3)
TBSO
O
Me
O
TBSO
S2
176.57
134.14
116.84
82.14
77.41 cdcl3
77.16 cdcl3
76.90 cdcl3
74.99
59.56
51.56
26.03
25.90
25.85
25.66
21.26
18.36
18.07
17.80
-4.02
-4.24
-4.46
-4.70
-4.95
-5.17
-5.37
-5.60
f1 (ppm)

^{1}H-NMR (500 MHz, $CDCl_3$)

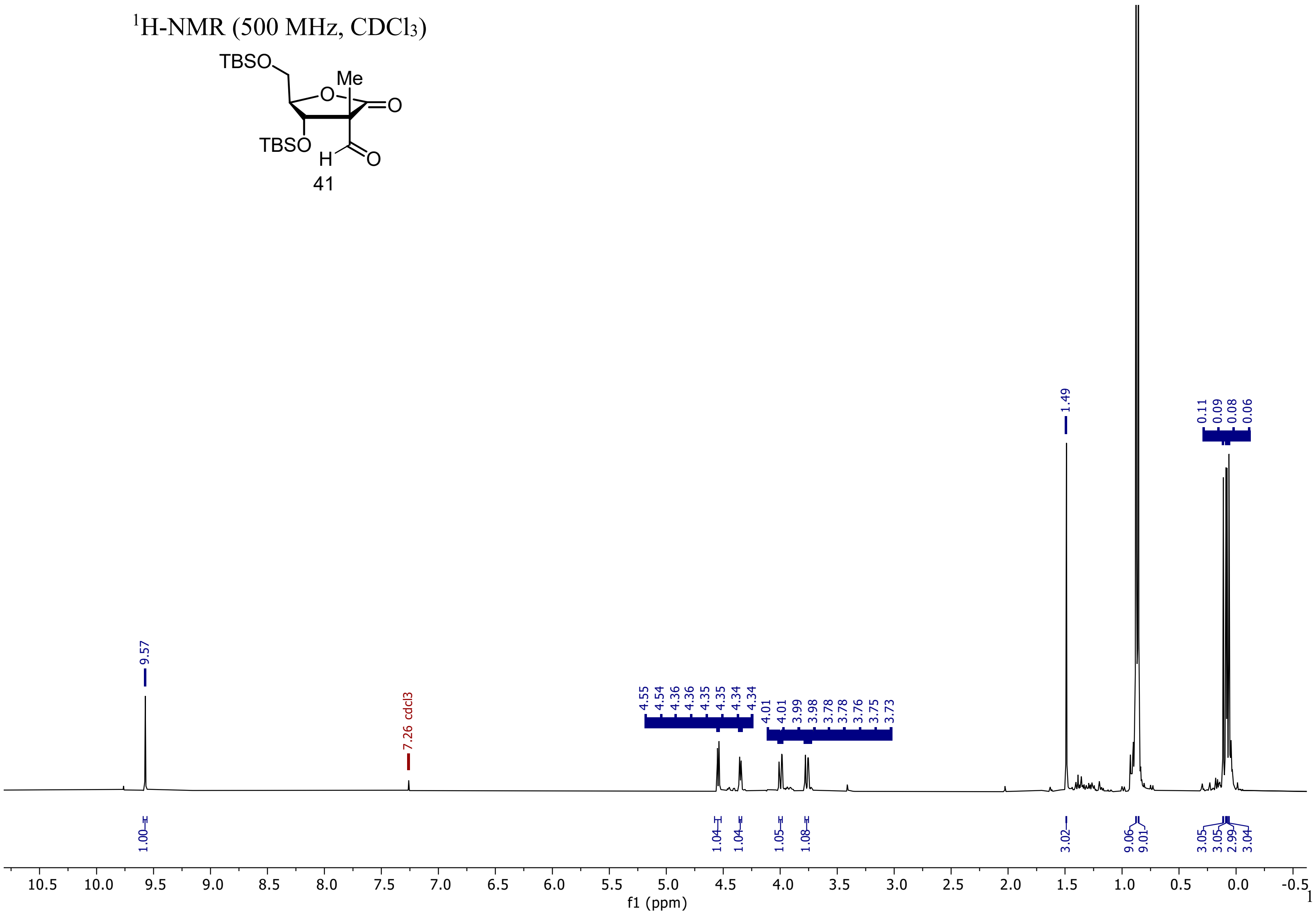

1

AM-ESP-873

$^{13}C\{^{1}H\}$-NMR (126 MHz, $CDCl_3$)

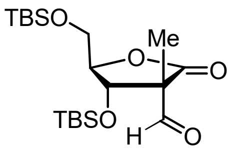

41

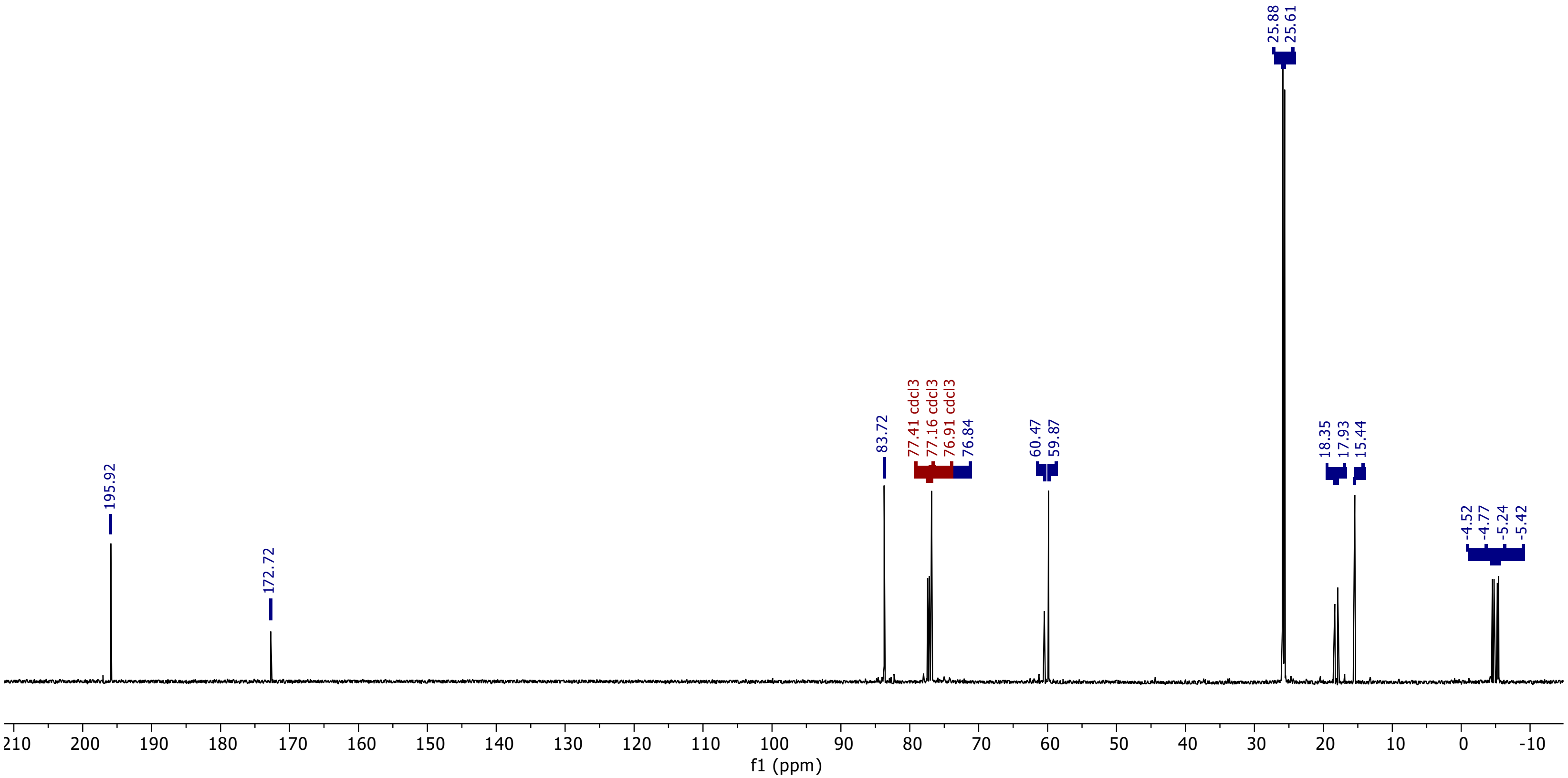

AM-ESP-800

^{1}H-NMR (500 MHz, $CDCl_3$)

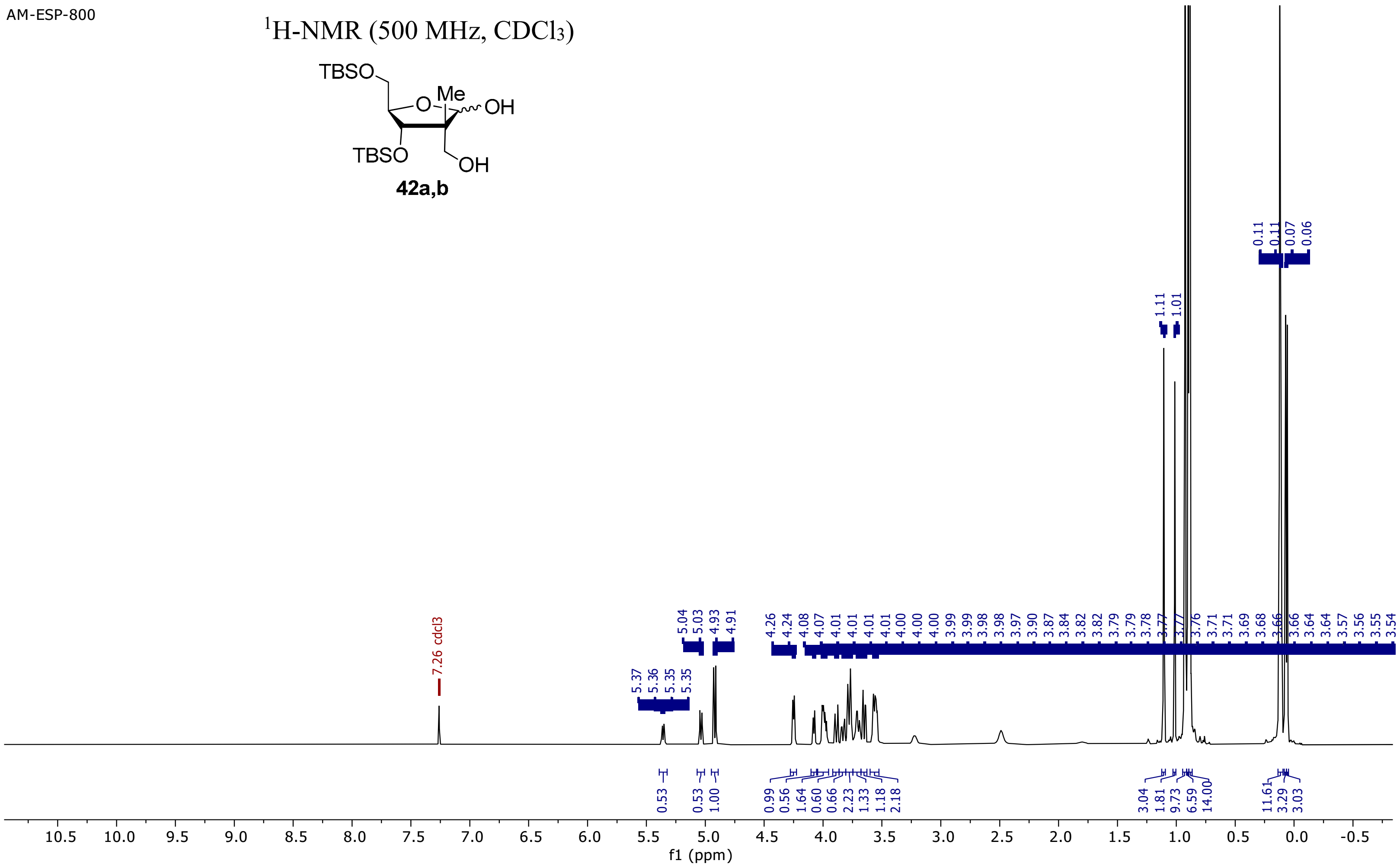

AM-ESP-800

$^{13}C\{^{1}H\}$-NMR (126 MHz, $CDCl_3$)

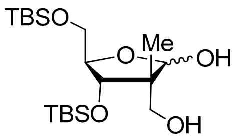

42a,b

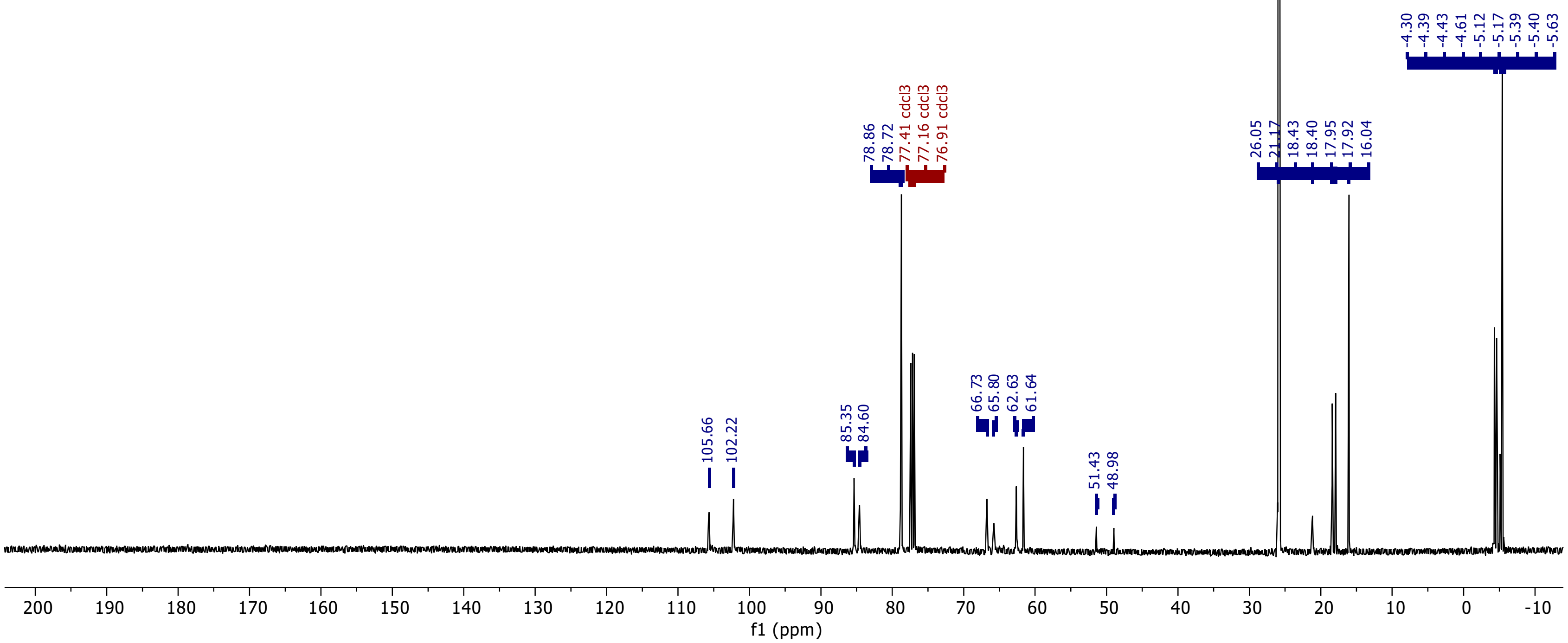

AM-ESP-828

^{1}H-NMR (500 MHz, $CDCl_3$)

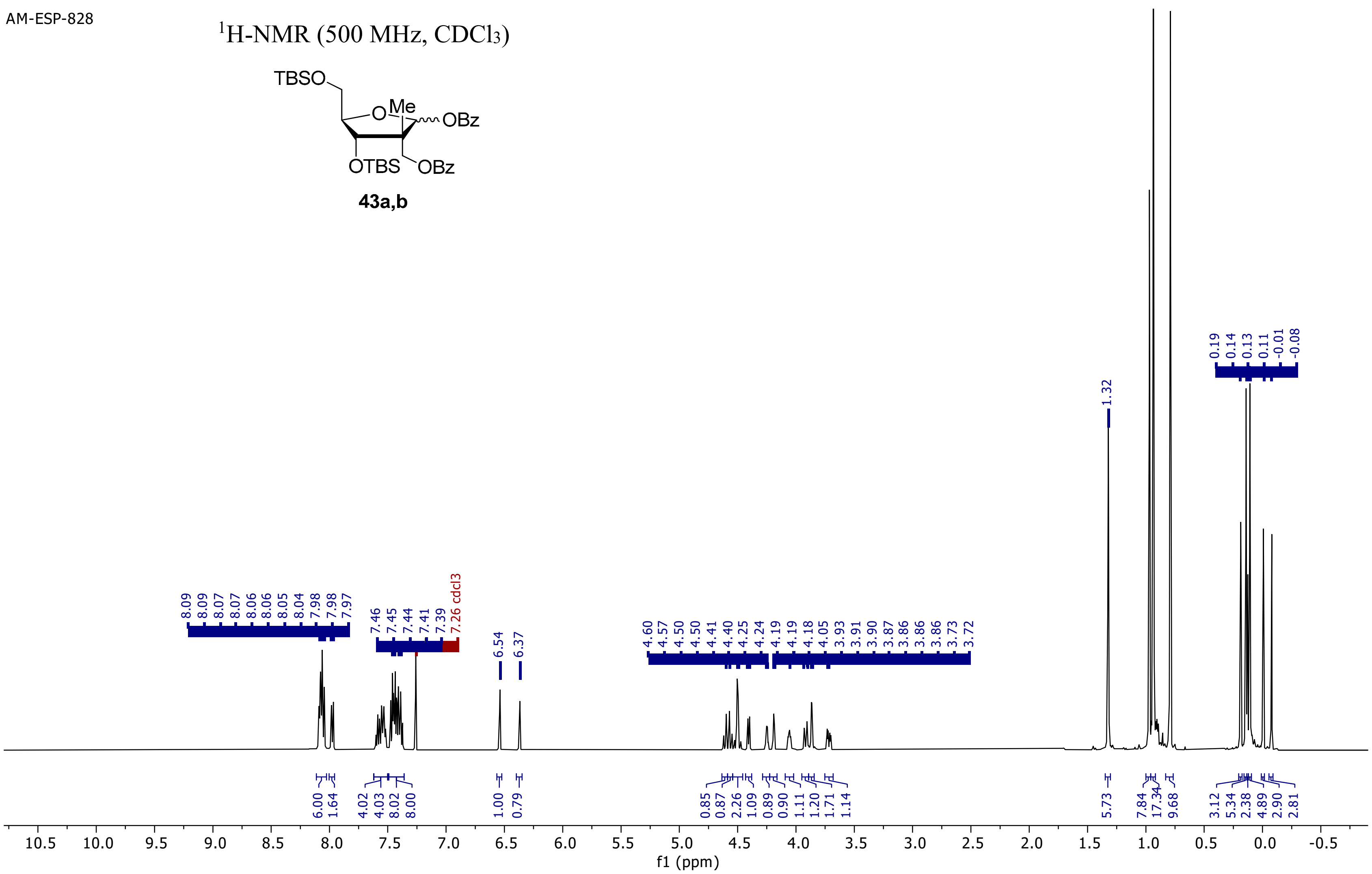

AM-ESP-828

$^{13}C\{^{1}H\}$-NMR (126 MHz, $CDCl_3$)

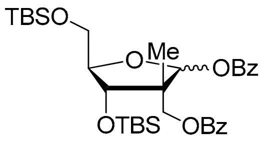

43a,b

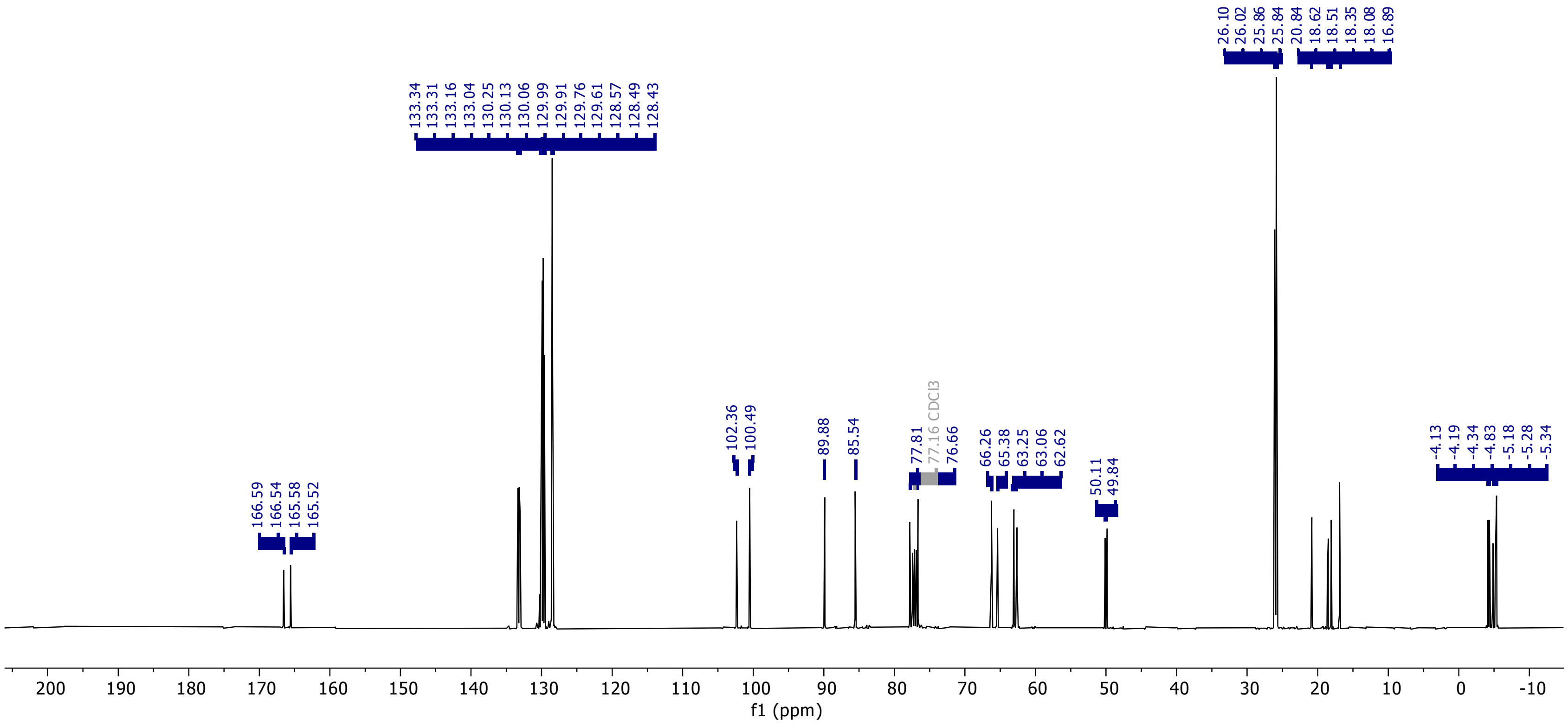

^{1}H-NMR (500 MHz, $CDCl_3$)

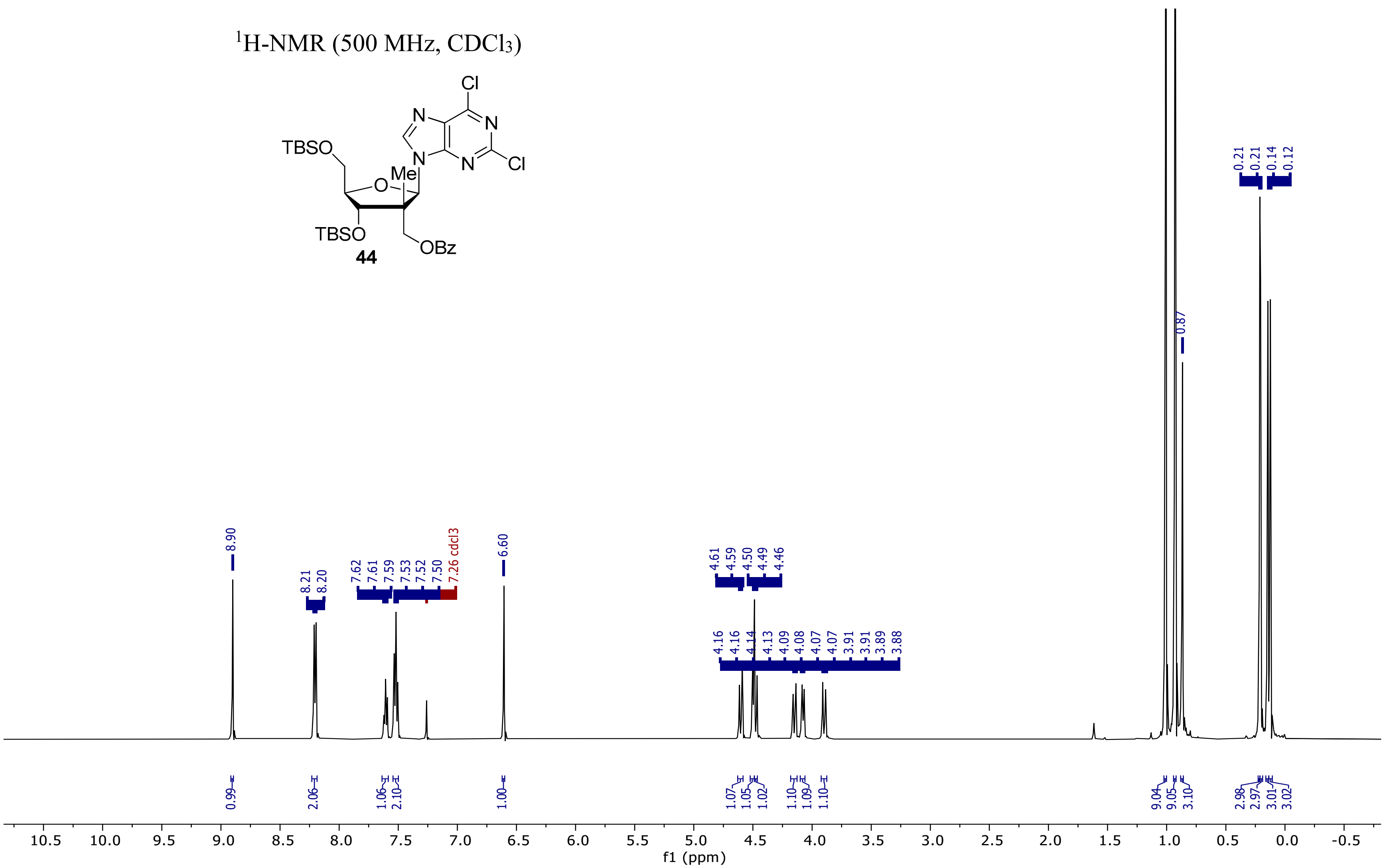

$^{13}C\{^{1}H\}$-NMR (126 MHz, $CDCl_3$)

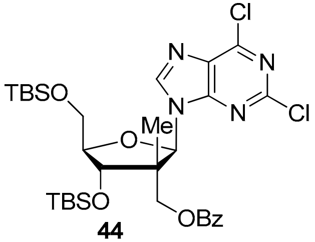

44

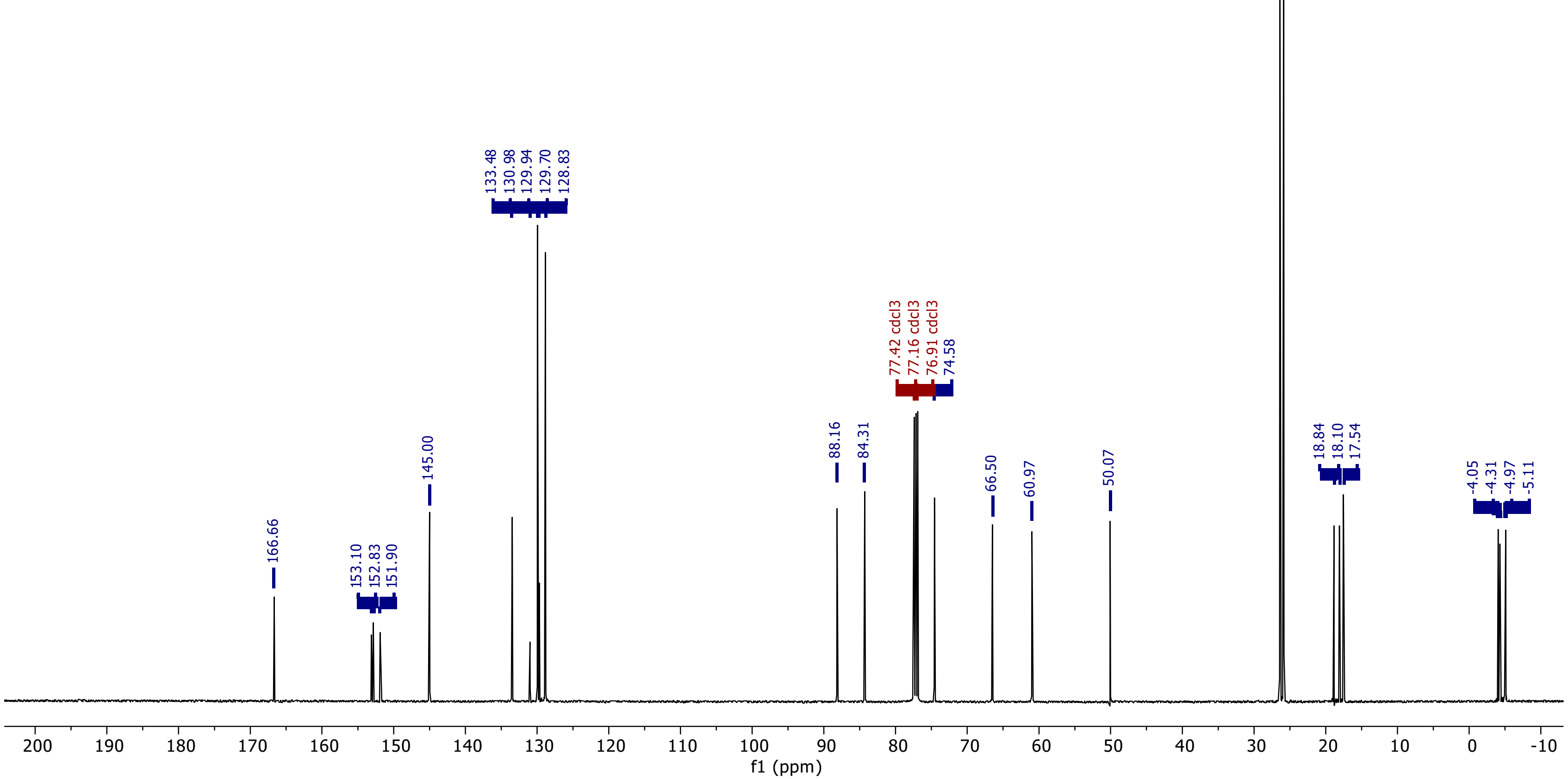

NOESY (500 MHz, CDCl3)

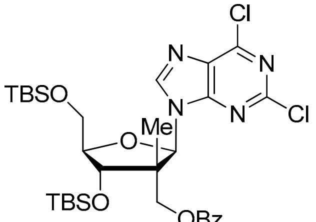

44

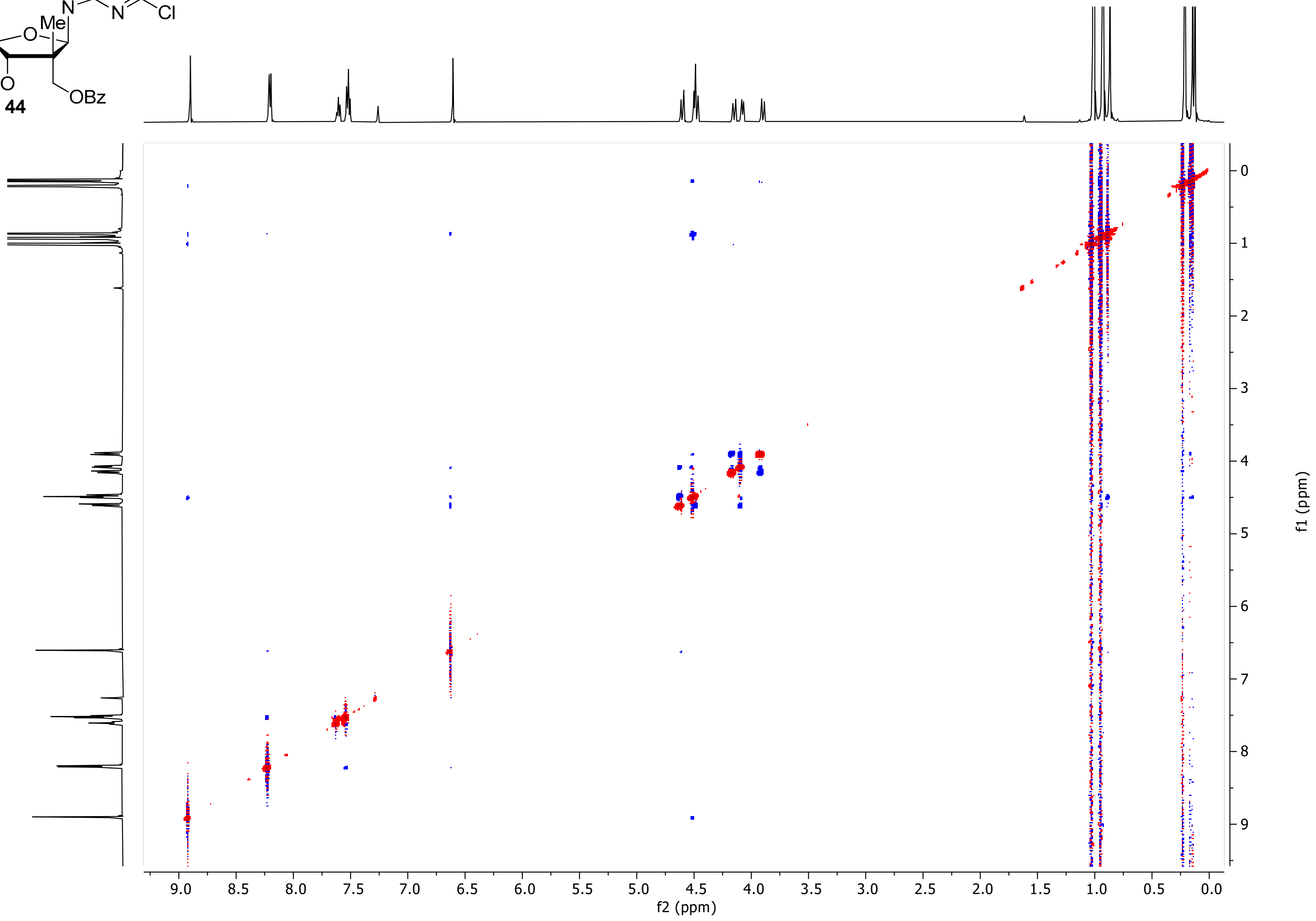

HMBC (500 MHz, $CDCl_3$)

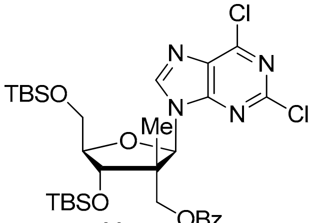

44

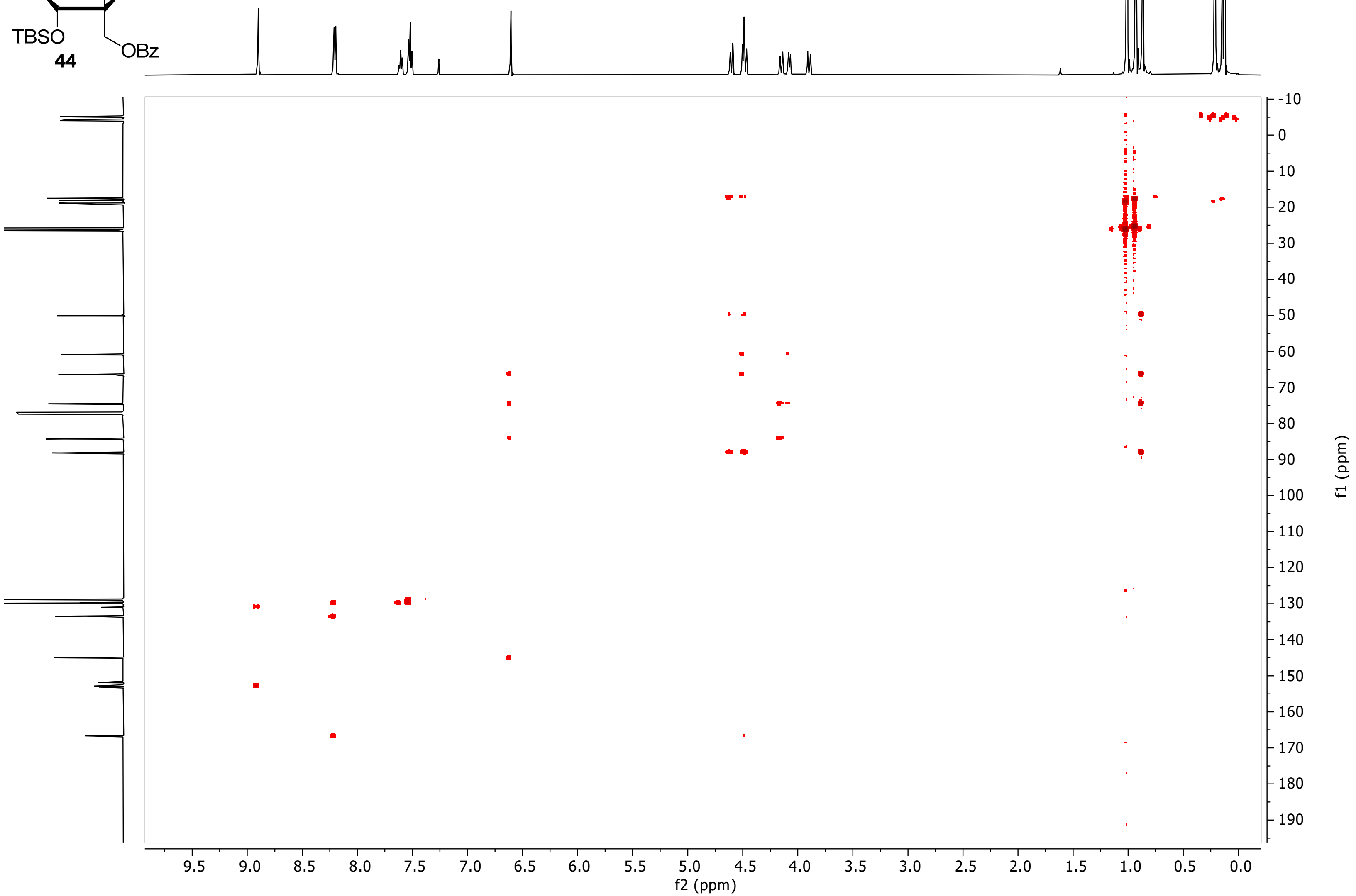

HSQC (500 MHz, $CDCl_3$)

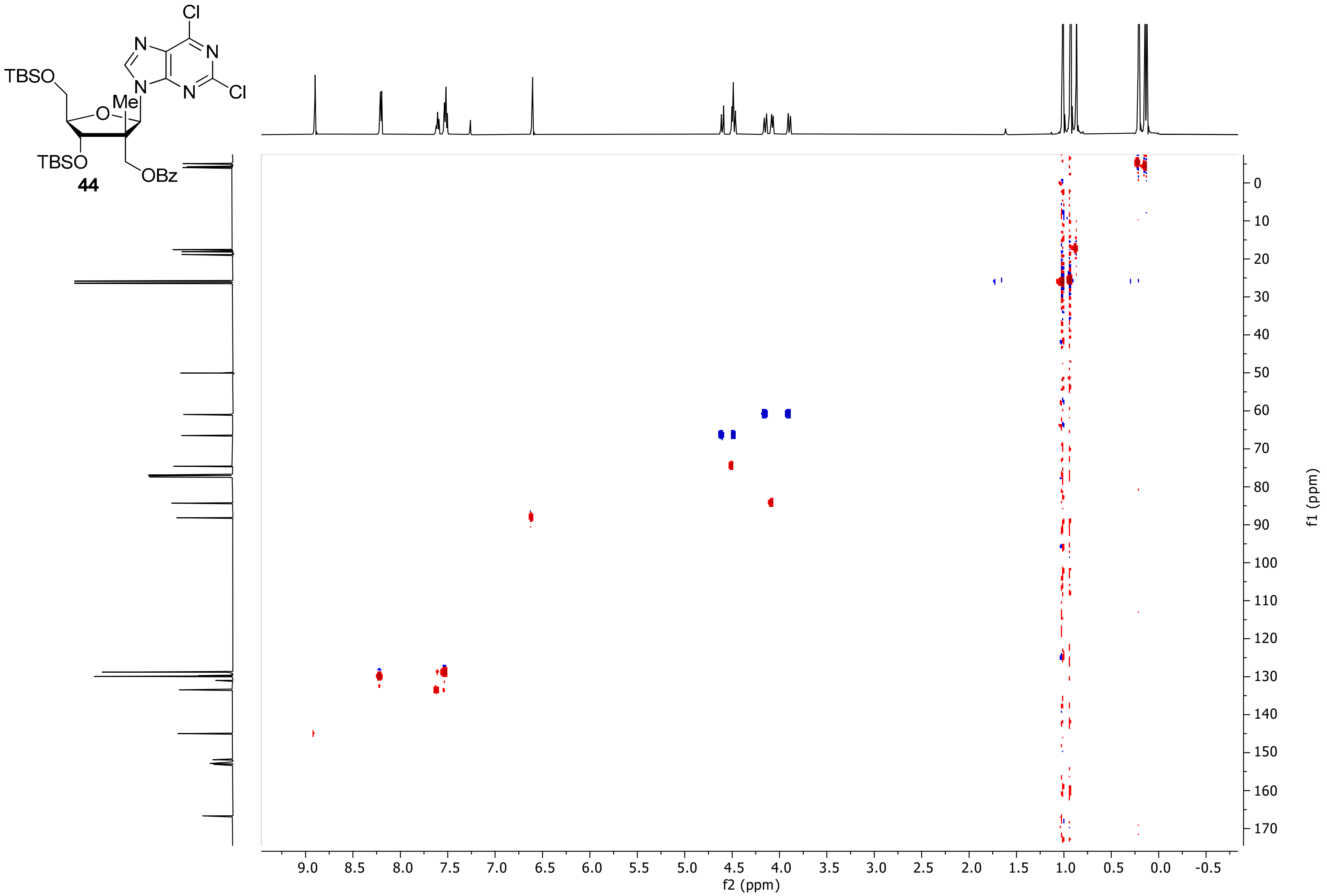

^{1}H-NMR (500 MHz, $CDCl_3$)

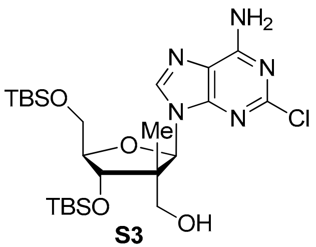

S3

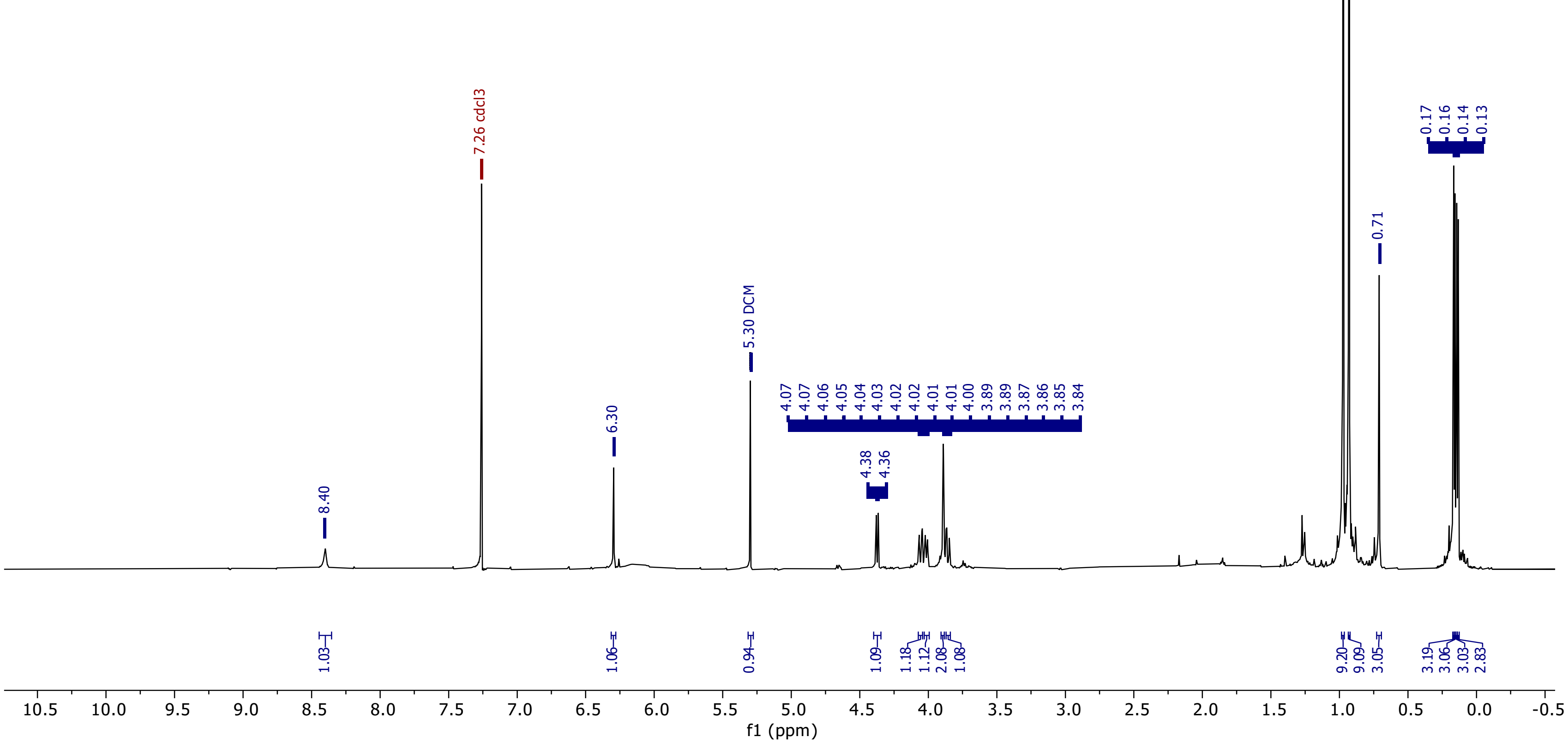

$^{13}C\{^{1}H\}$-NMR (126 MHz, $CDCl_3$)

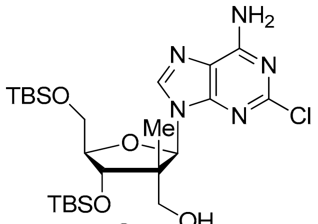

S3

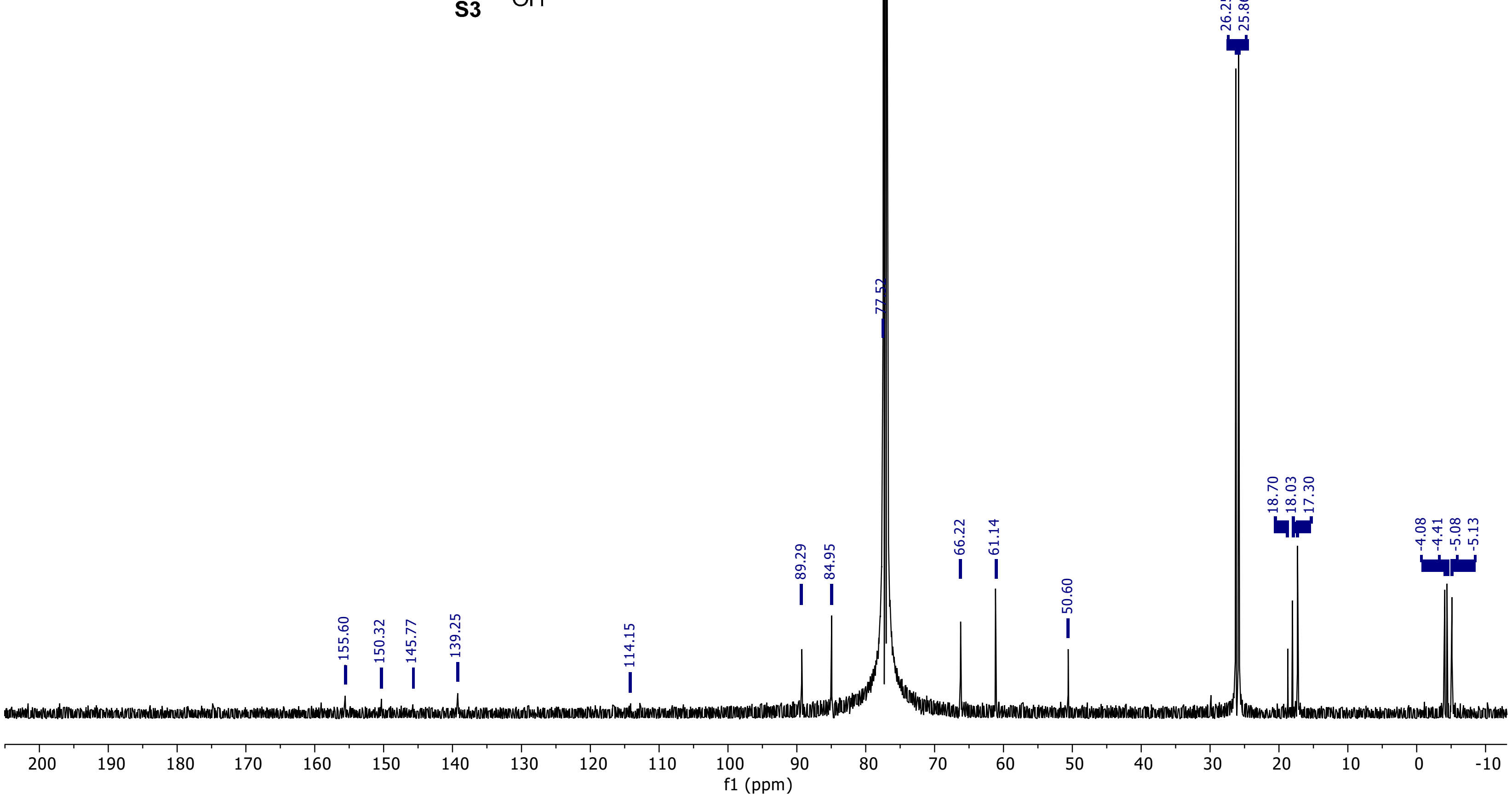

^{1}H-NMR (500 MHz, CD_3OD)

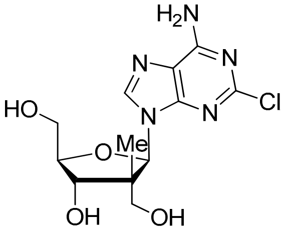

45

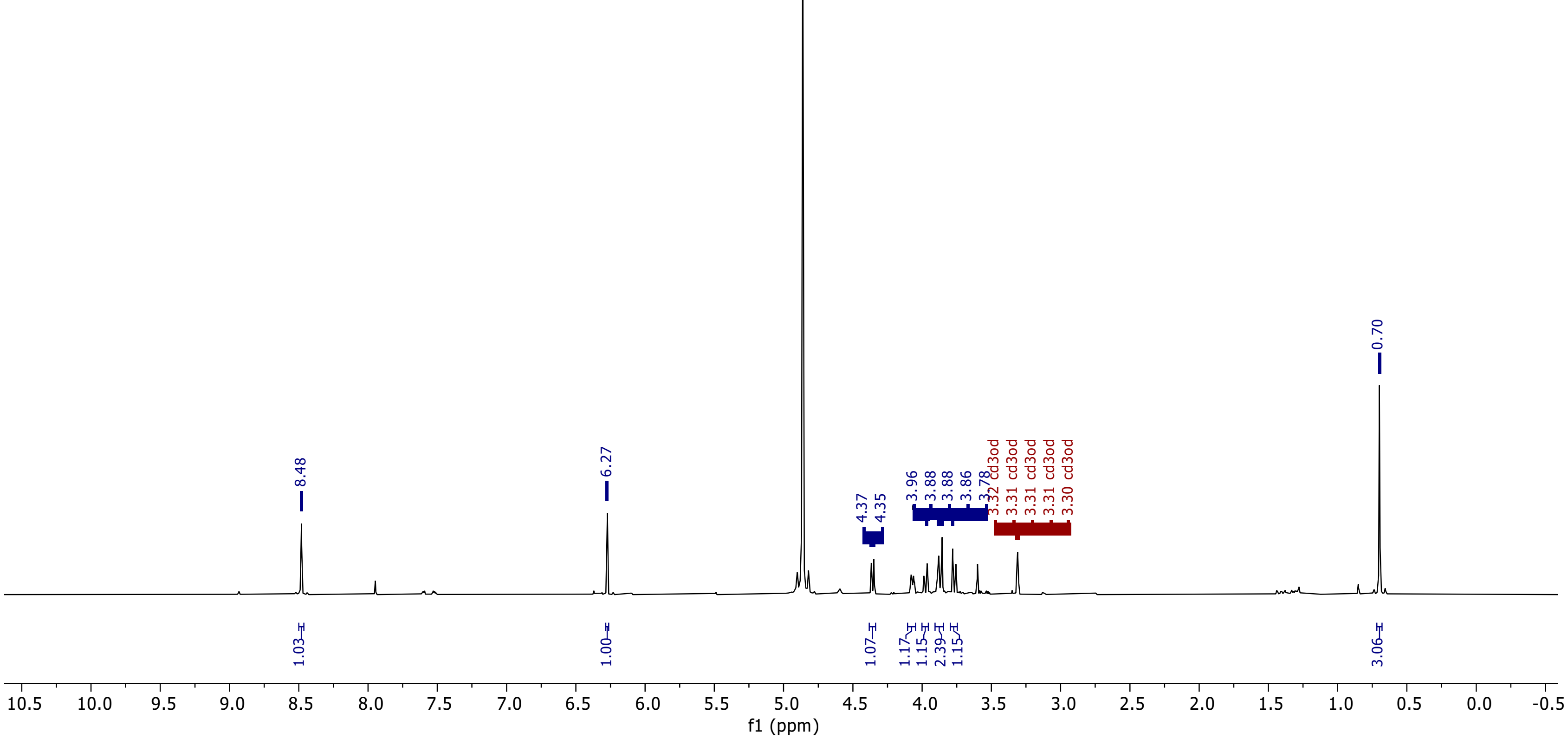

$^{13}C\{^{1}H\}$-NMR (126 MHz, CD_3OD)

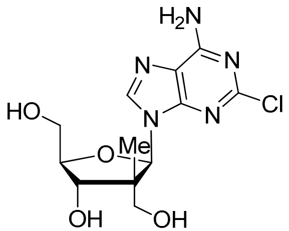

45

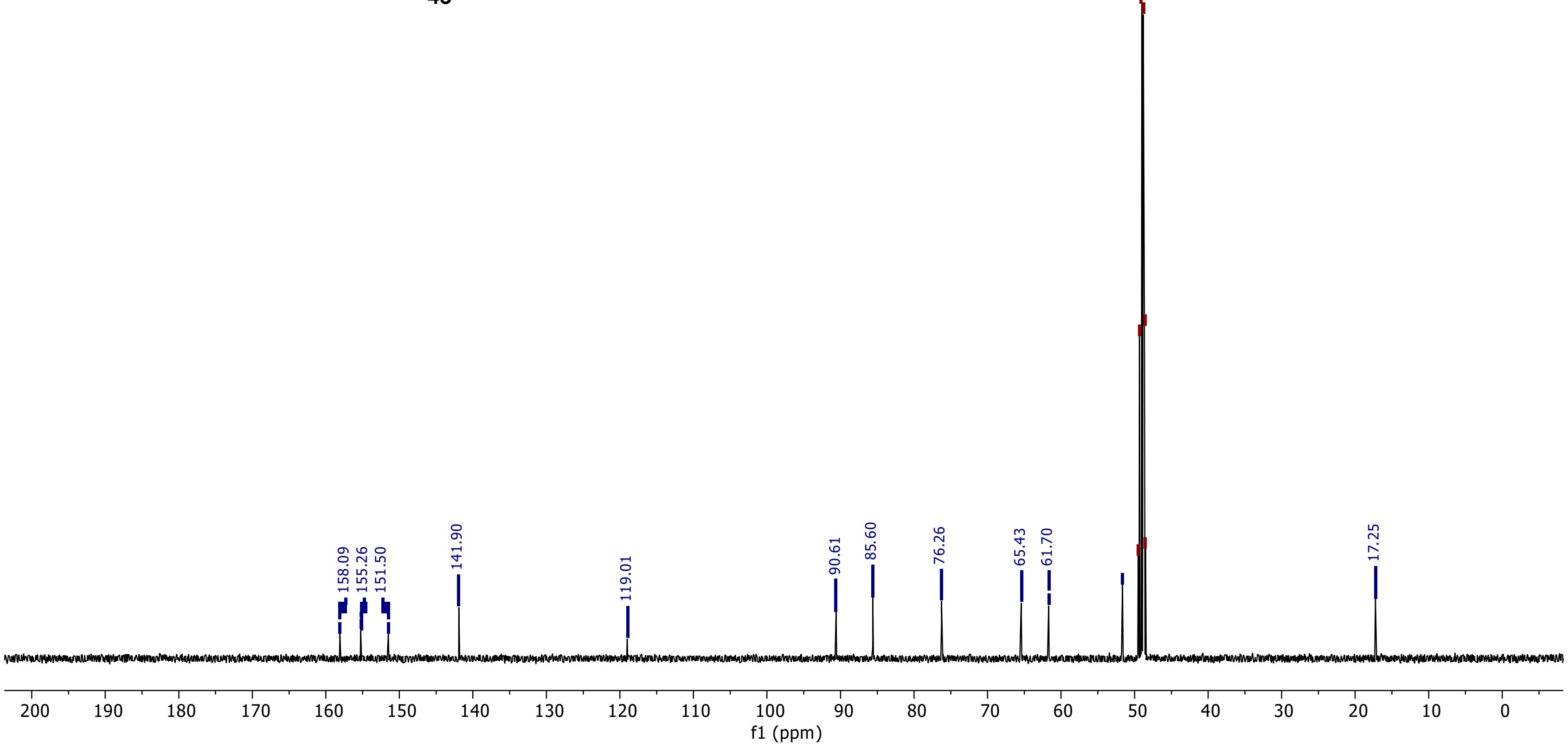

AM-ESP-873

^{1}H-NMR (500 MHz, CD_3OD)

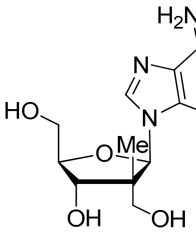

46

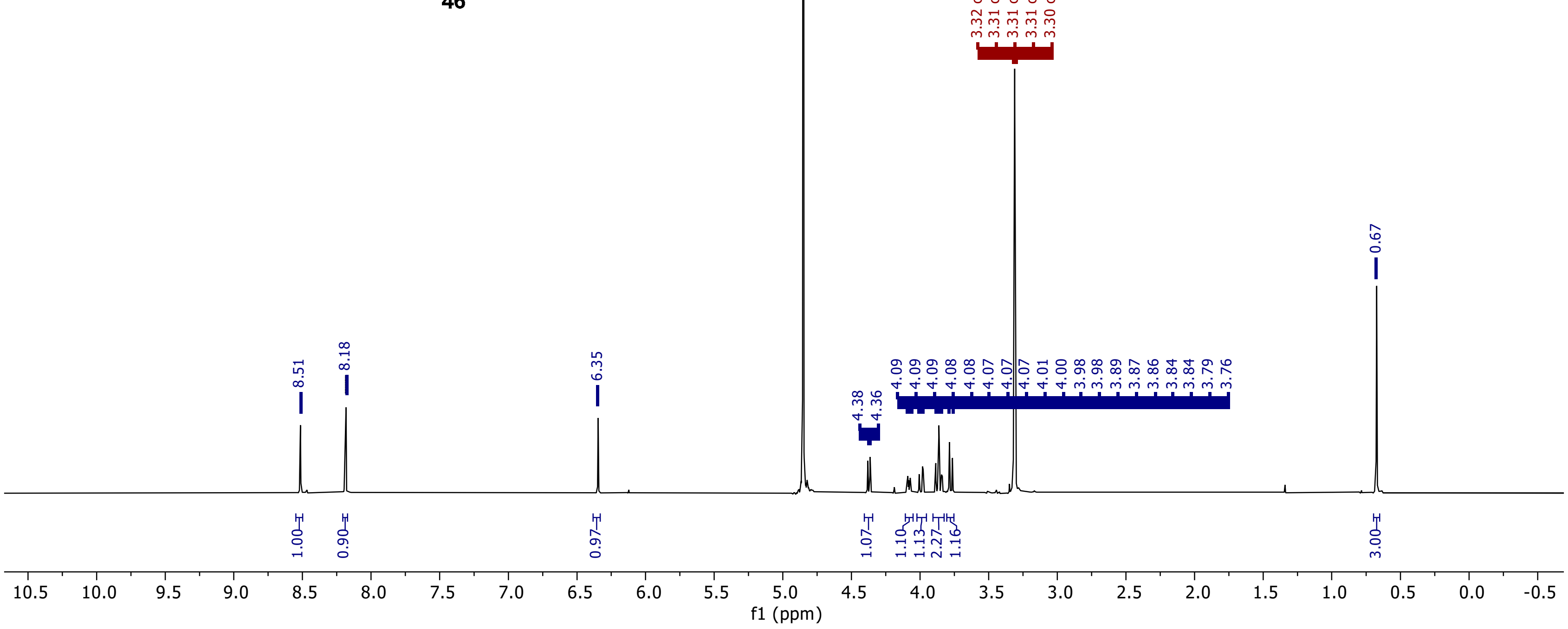

AM-ESP-880

$^{13}C\{^{1}H\}$-NMR (126 MHz, CD_3OD)

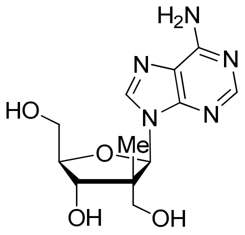

46

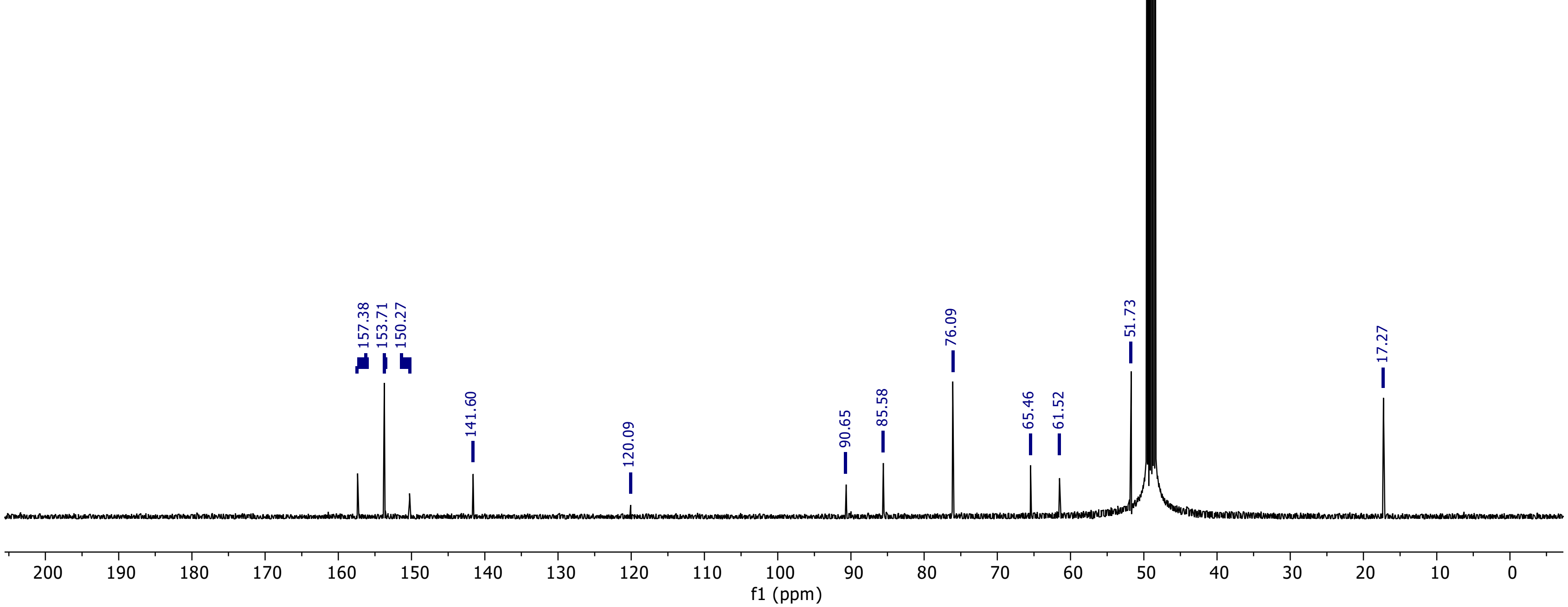

^{1}H-NMR (500 MHz, D_2O)

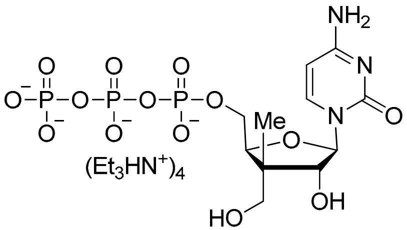

2 (**LCB-2330**)

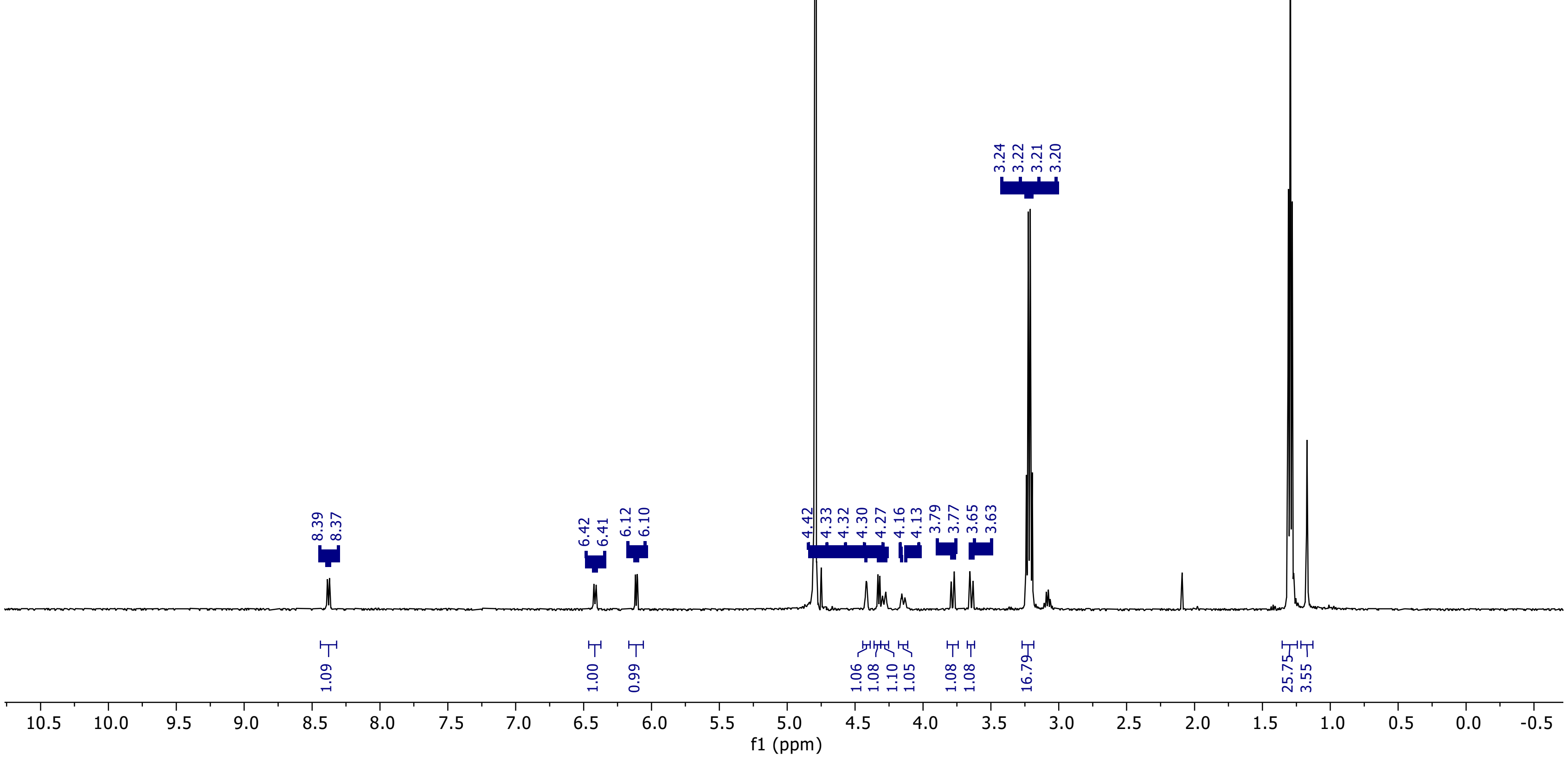

$^{13}C\{^{1}H\}$-NMR (126 MHz, D_2O)

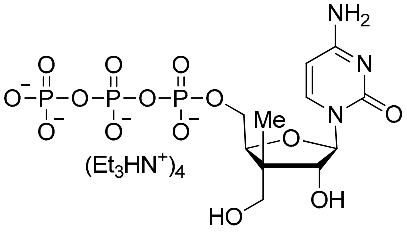

2 (**LCB-2330**)

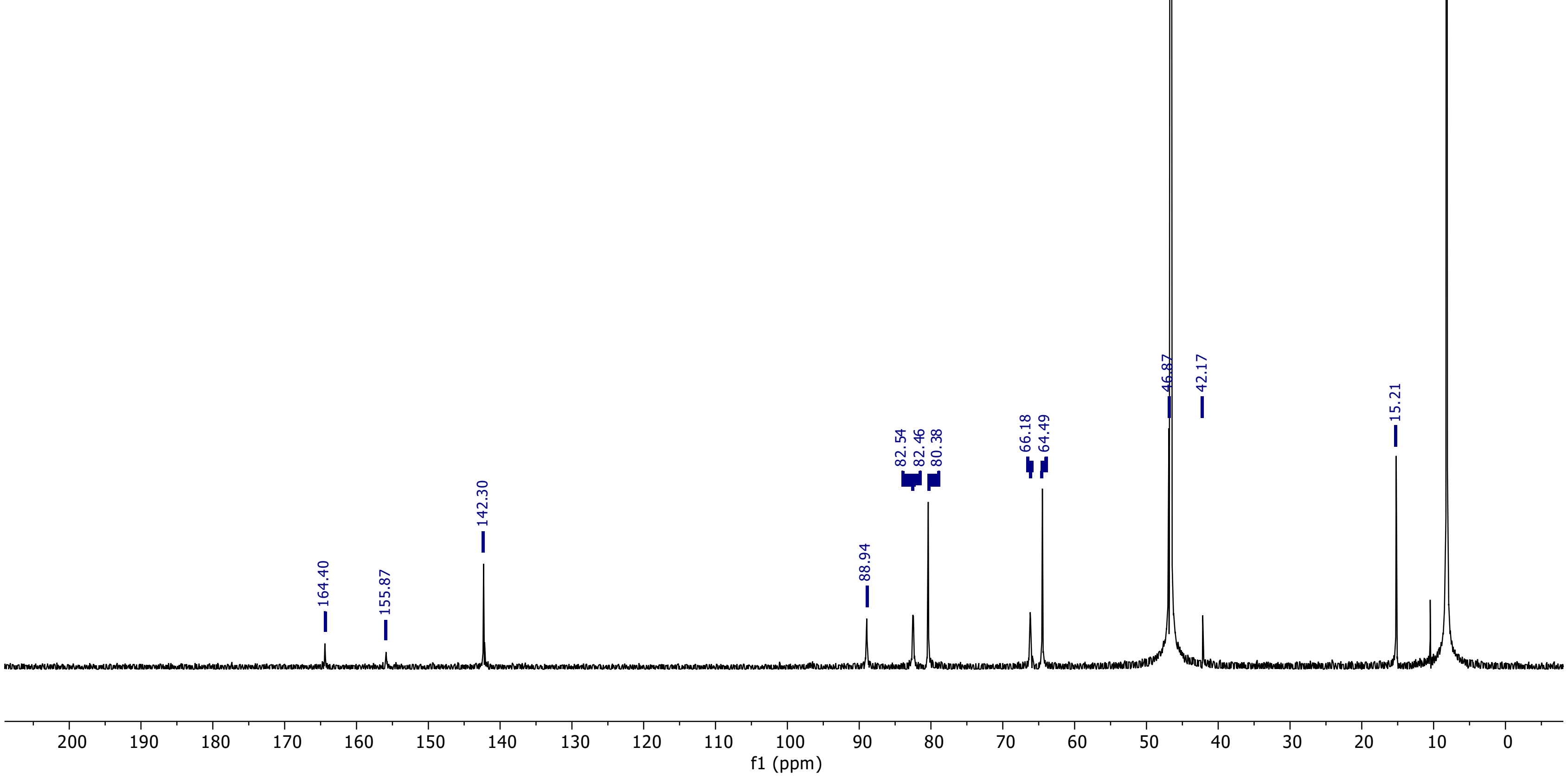

^{31}PNMR (162 MHz, D_2O)

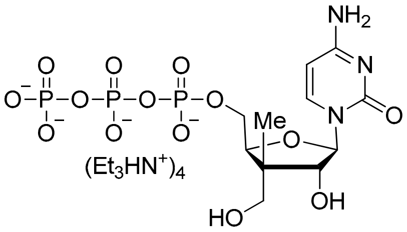

2 (**LCB-2330**)

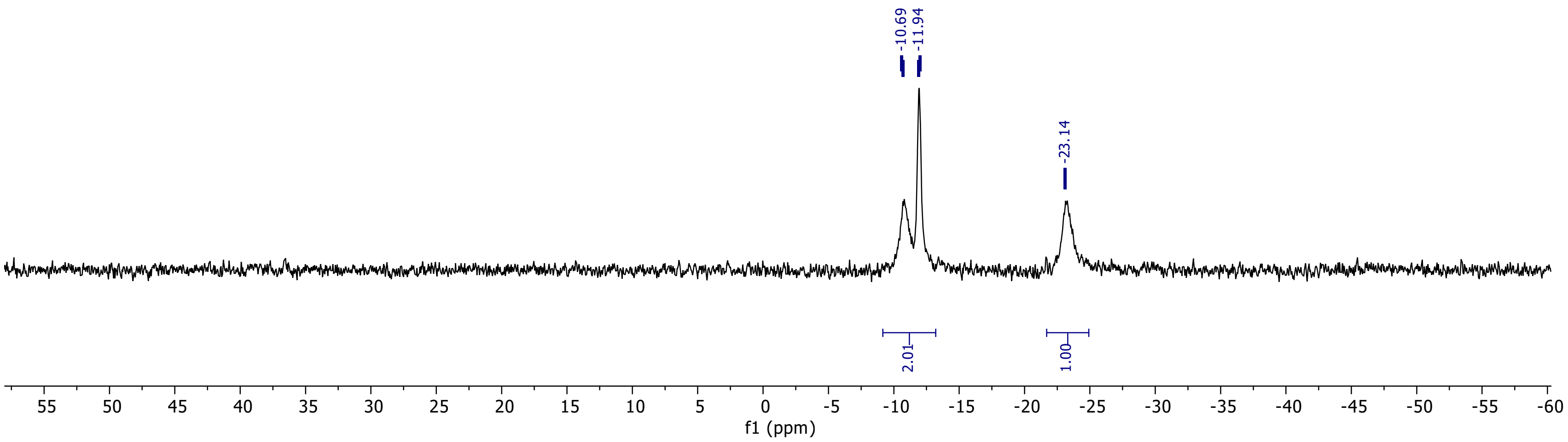

^{1}H-NMR (500 MHz, D_2O)

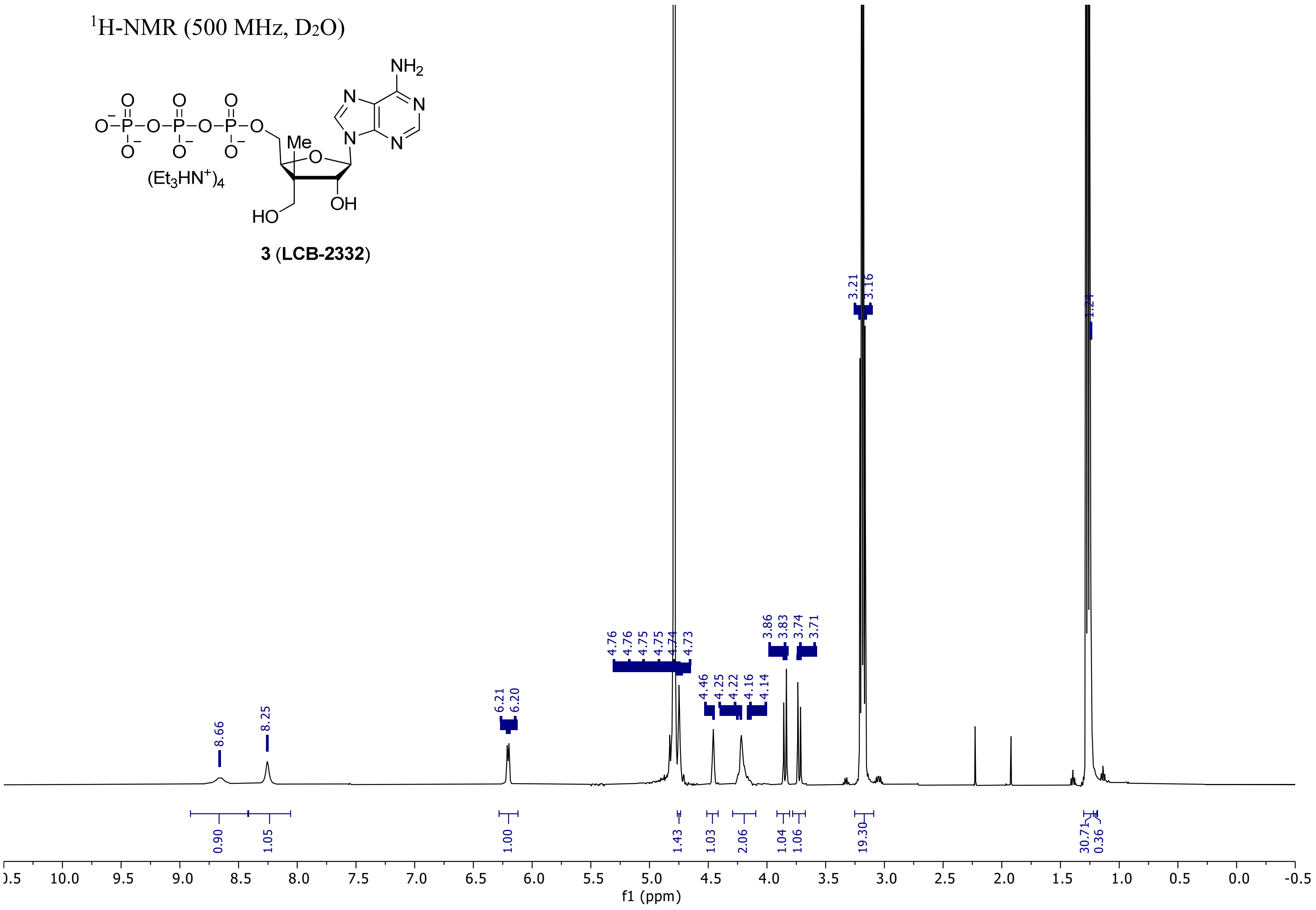

3 (**LCB-2332**)

$^{13}C\{^1H\}$-NMR (176 MHz, D_2O)

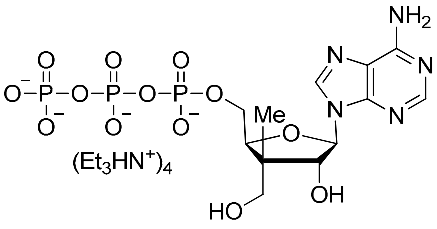

3 (**LCB-2332**)

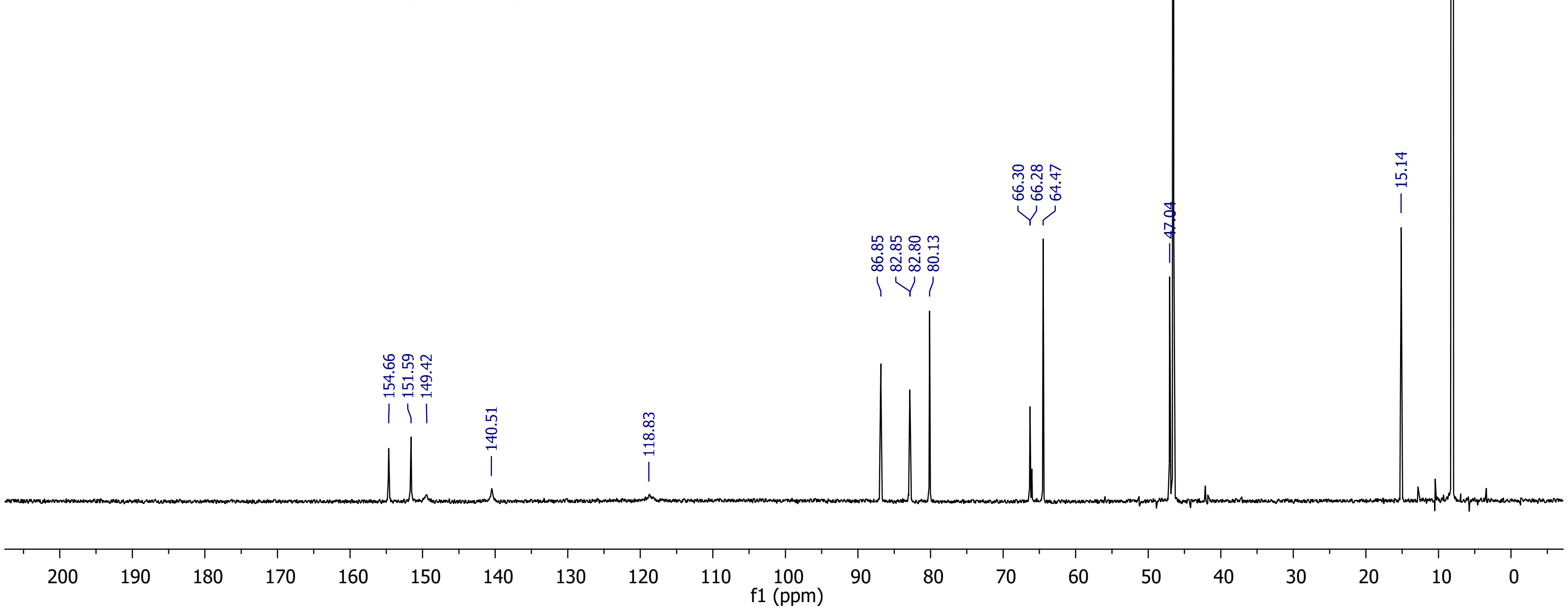

^{31}P NMR (162 MHz, D_2O)

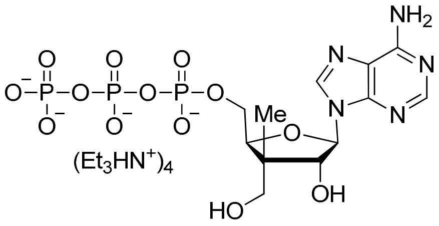

3 (**LCB-2332**)

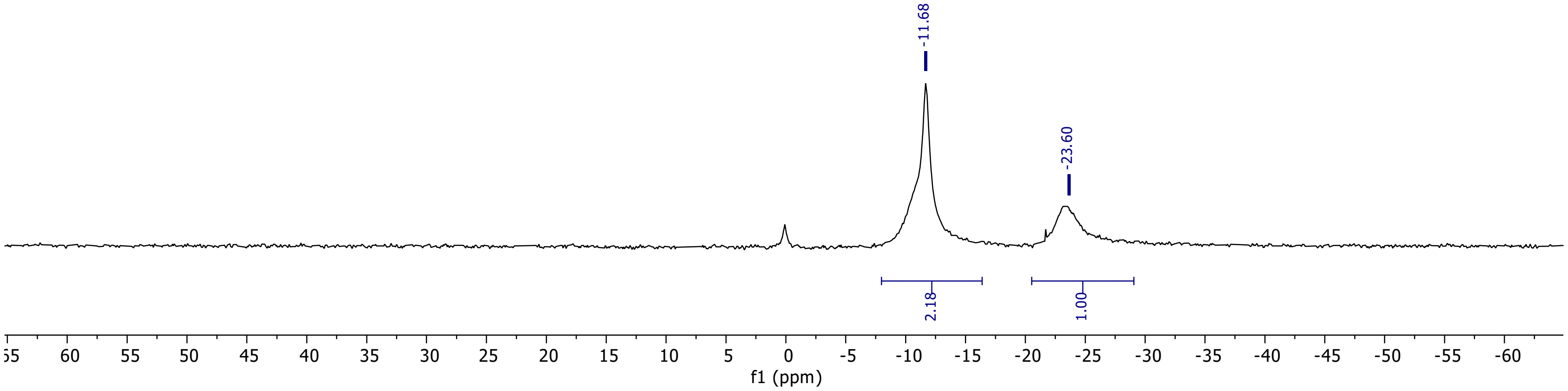

AM-ESP-765

^{1}H-NMR (500 MHz, D_2O)

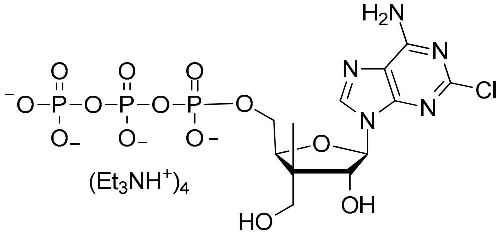

4 (**LCB 2337**)

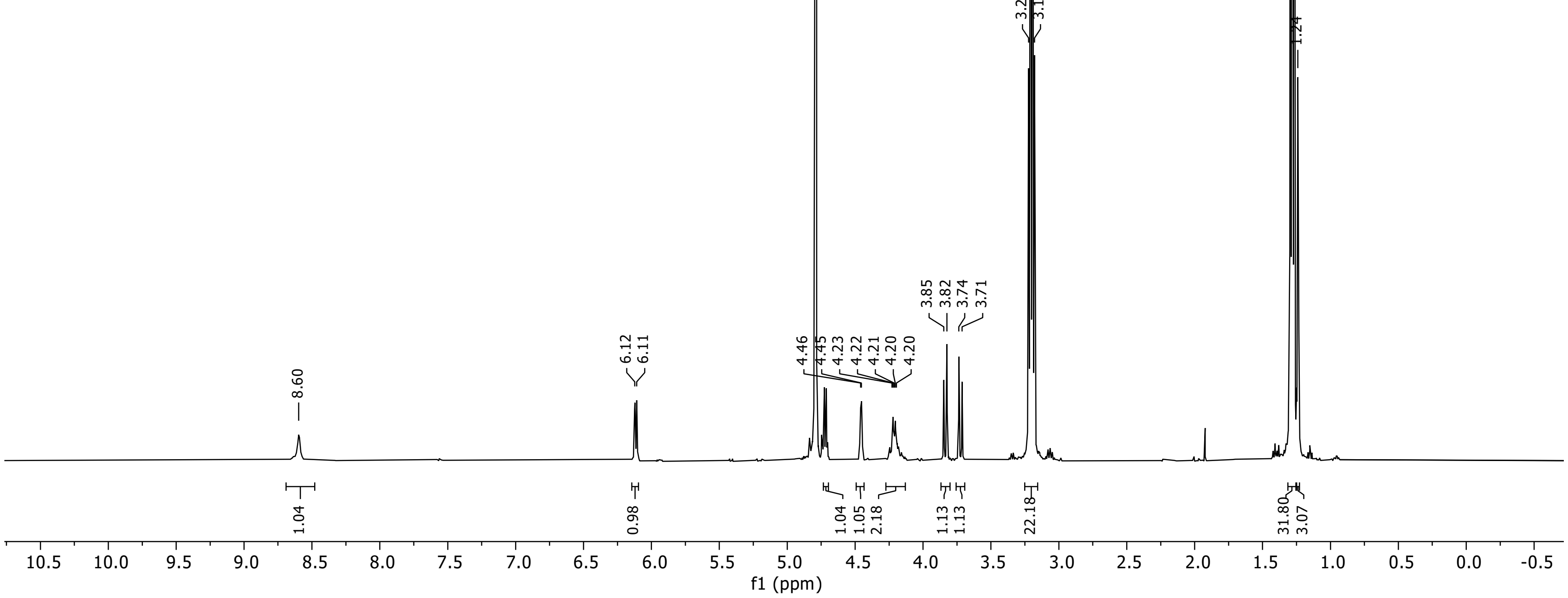

AM-ESP-765

$^{13}C\{^{1}H\}$-NMR (126 MHz, D_2O)

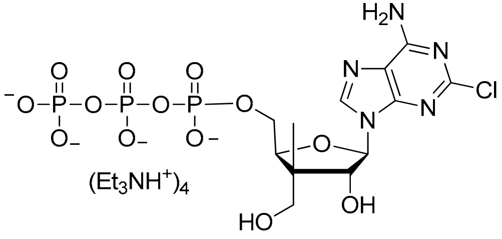

4 (**LCB 2337**)

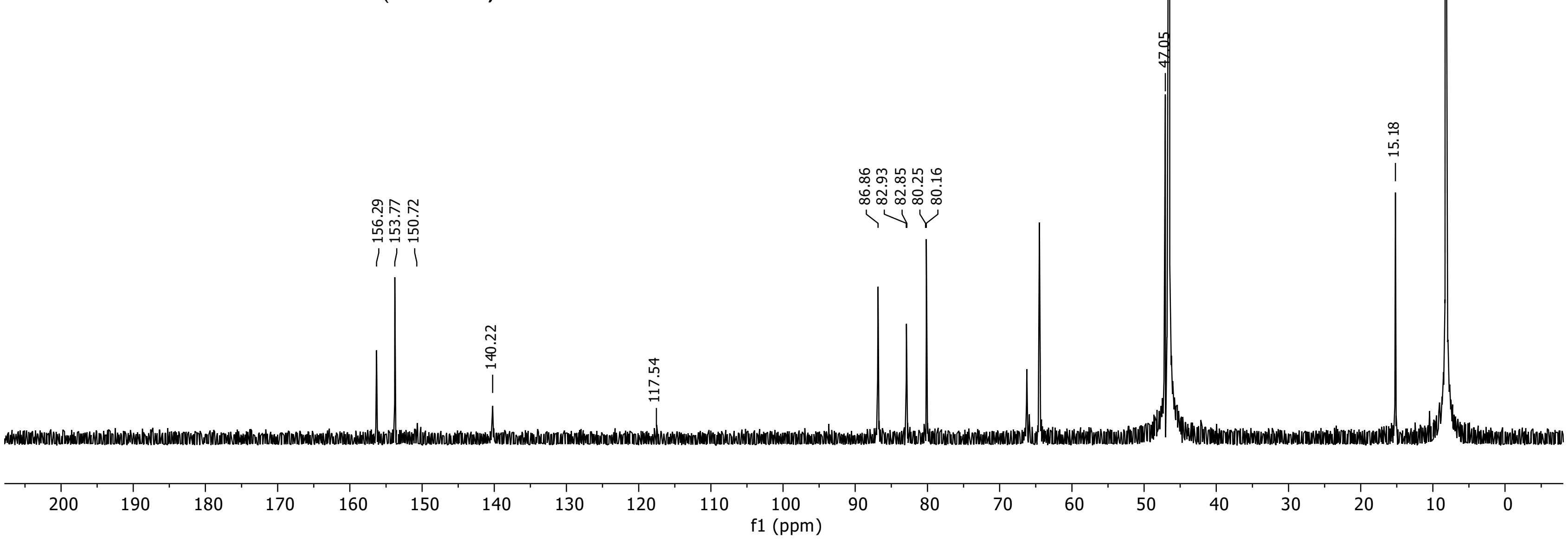

AM-ESP-765

^{31}P-NMR (162 MHz, D_2O)

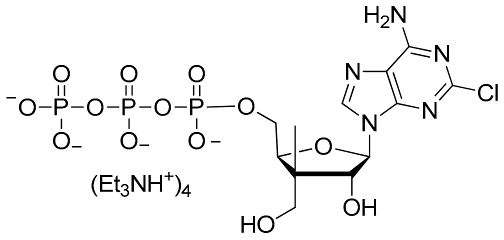

4 (**LCB 2337**)

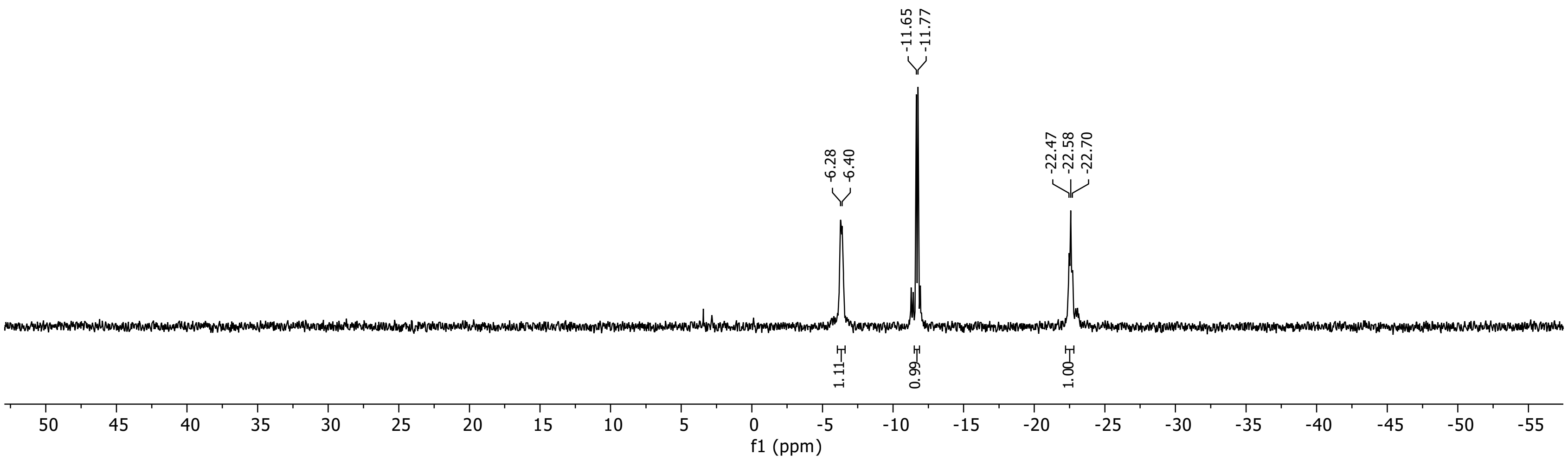

AM-ESP-327

^{1}H-NMR (700 MHz, D_2O)

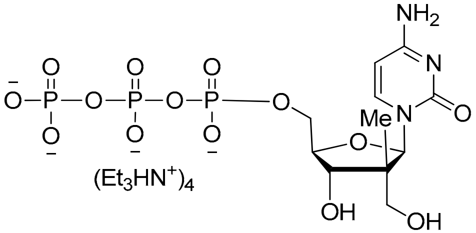

5 (**LCB 2289**)

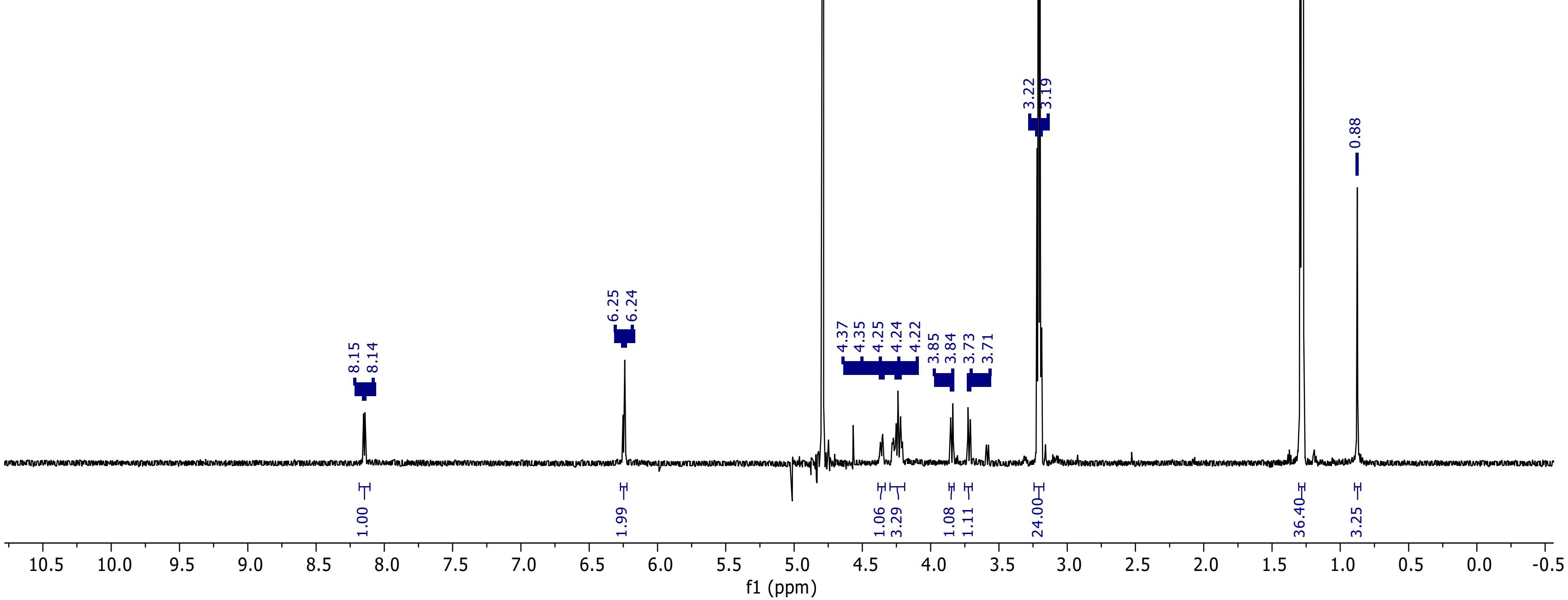

AM-ESP-327

$^{13}C\{^1H\}$-NMR (176 MHz, D_2O)

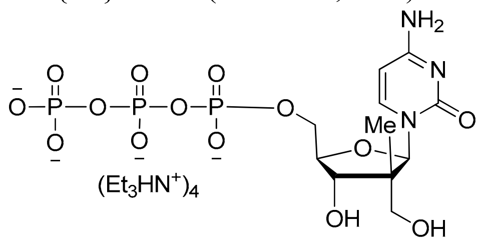

5 (**LCB 2289**)

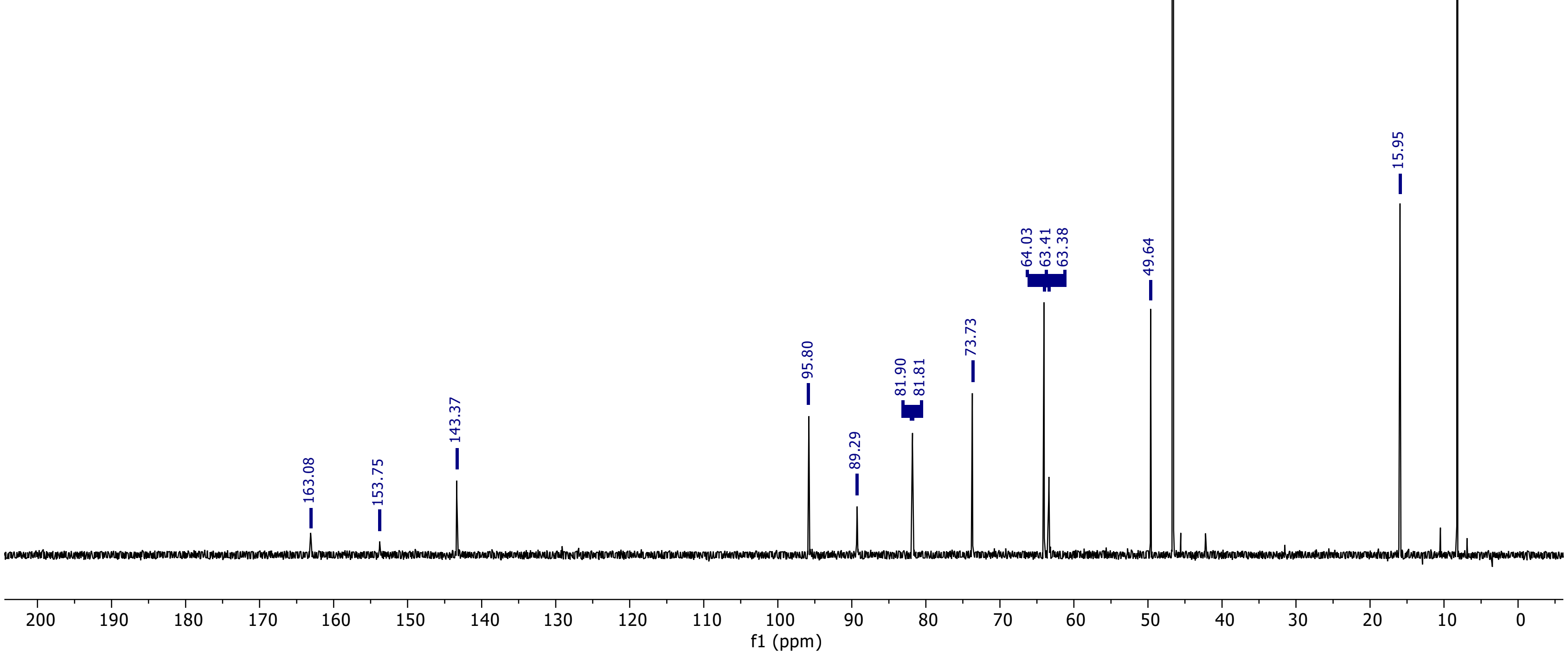

AM-ESP-327

^{31}P-NMR (162 MHz, D_2O)

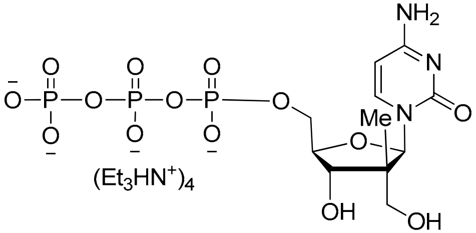

5 (**LCB 2289**)

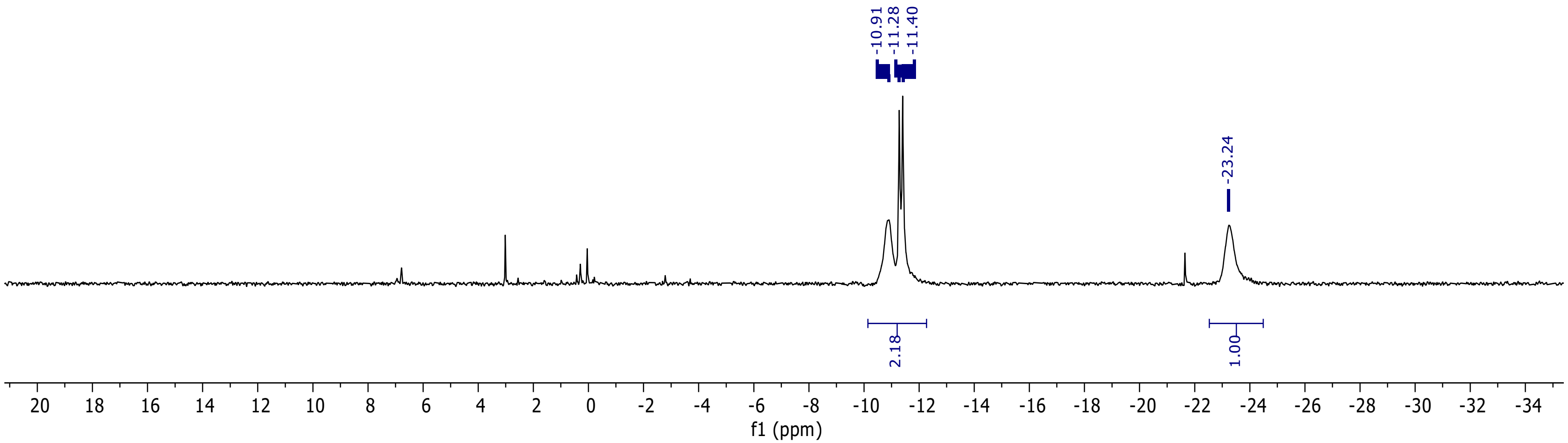

AM-ESP-1011

^{1}H-NMR (500 MHz, D_2O)

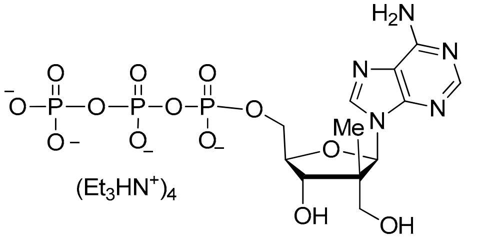

6 (**LCB 2344**)

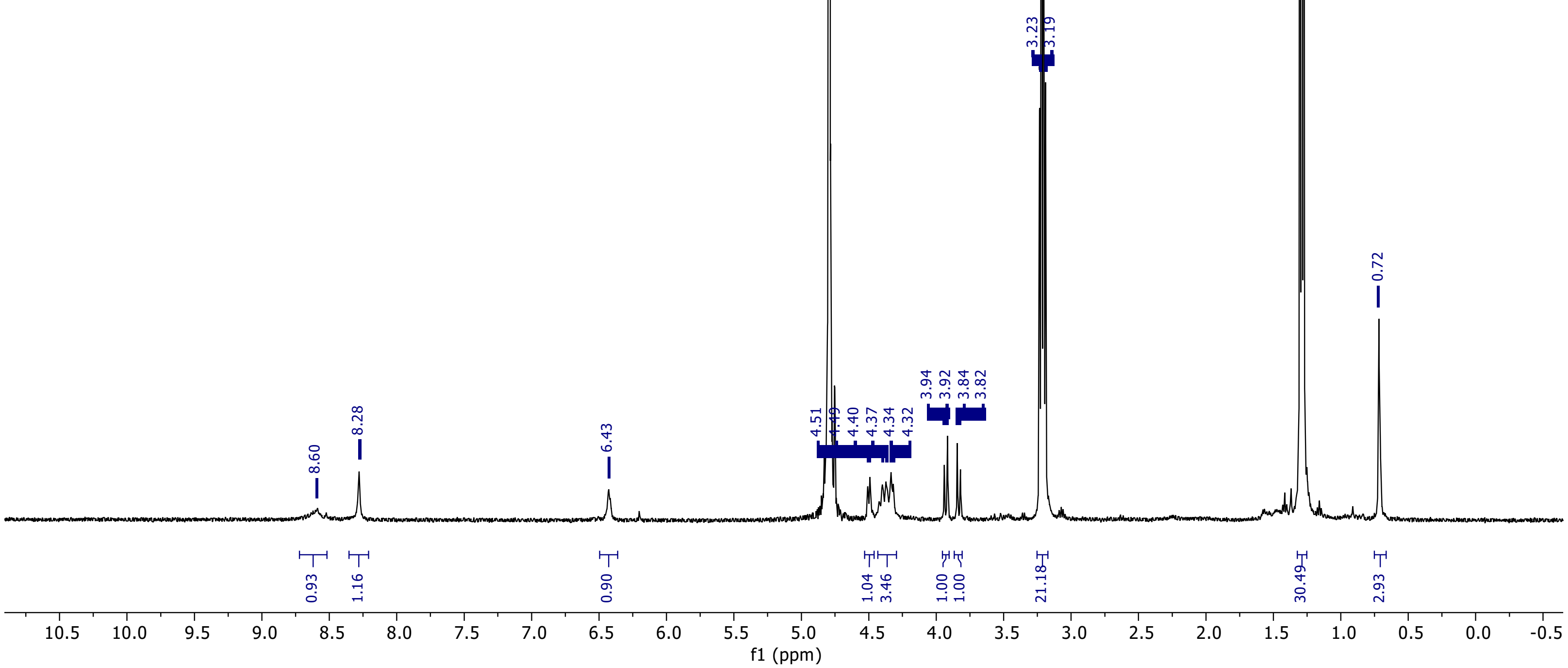

AM-ESP-875

$^{13}C\{^{1}H\}$-NMR (176 MHz, D_2O)

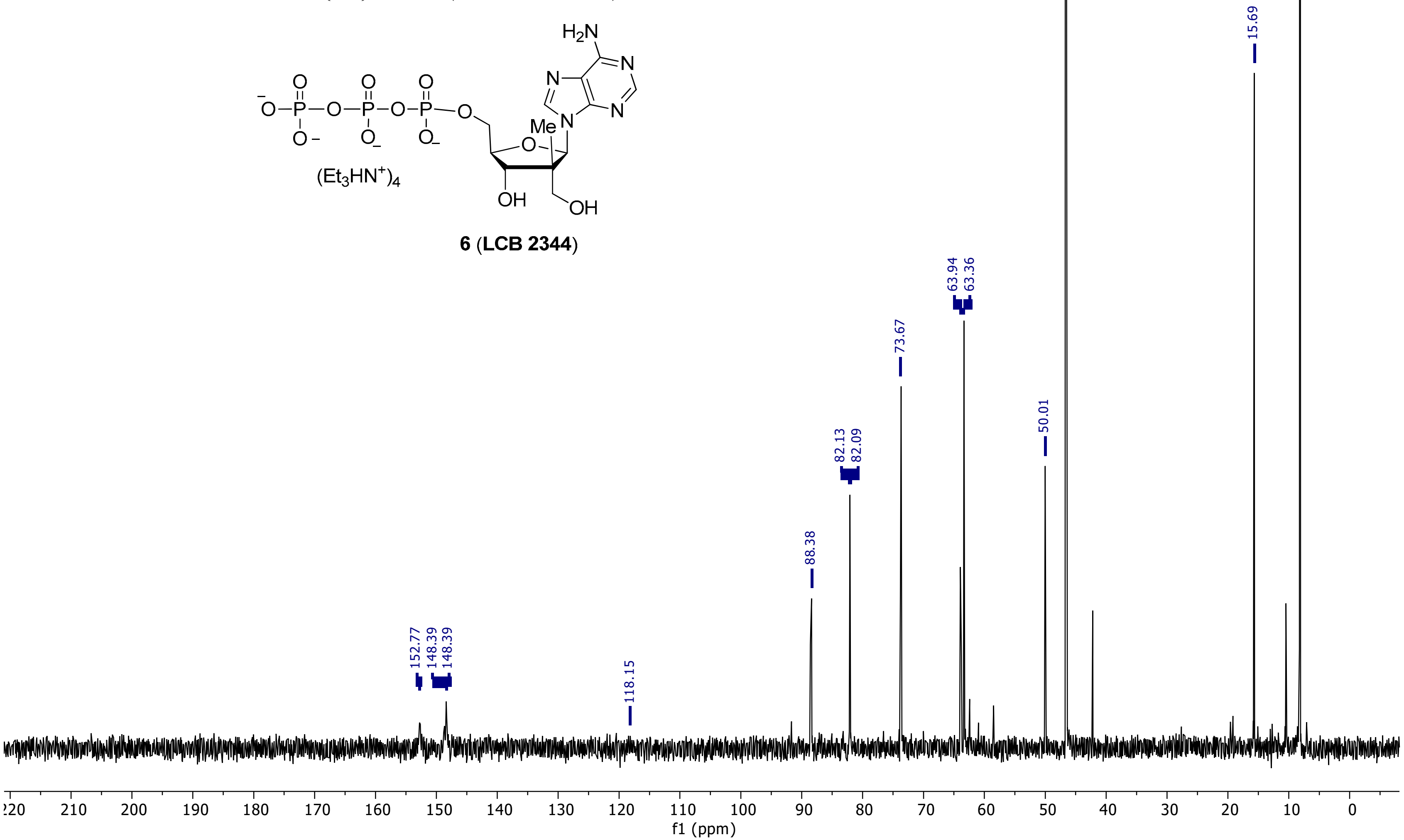

AM-ESP-878

^{31}P-NMR (162 MHz, D_2O)

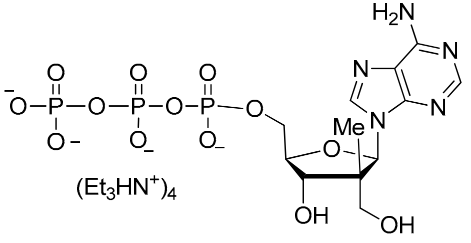

6 (**LCB 2344**)

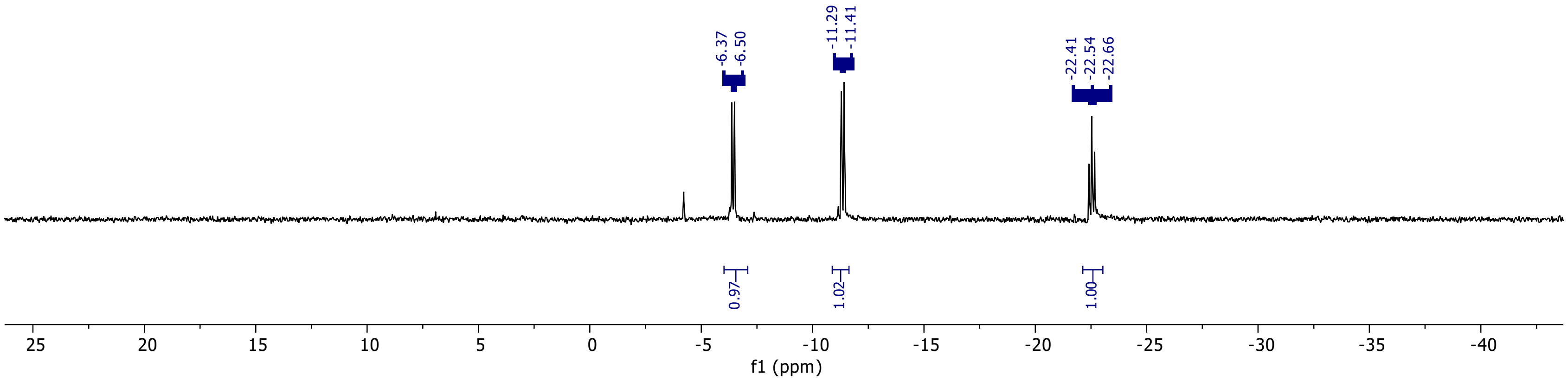

AM-ESP-854

^{1}H-NMR (700 MHz, D_2O)

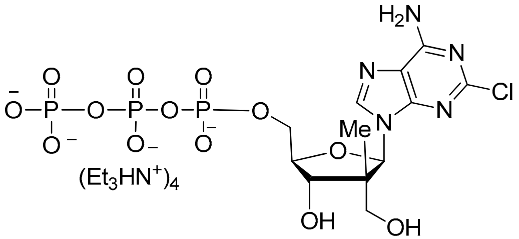

7 (**LCB 2279**)

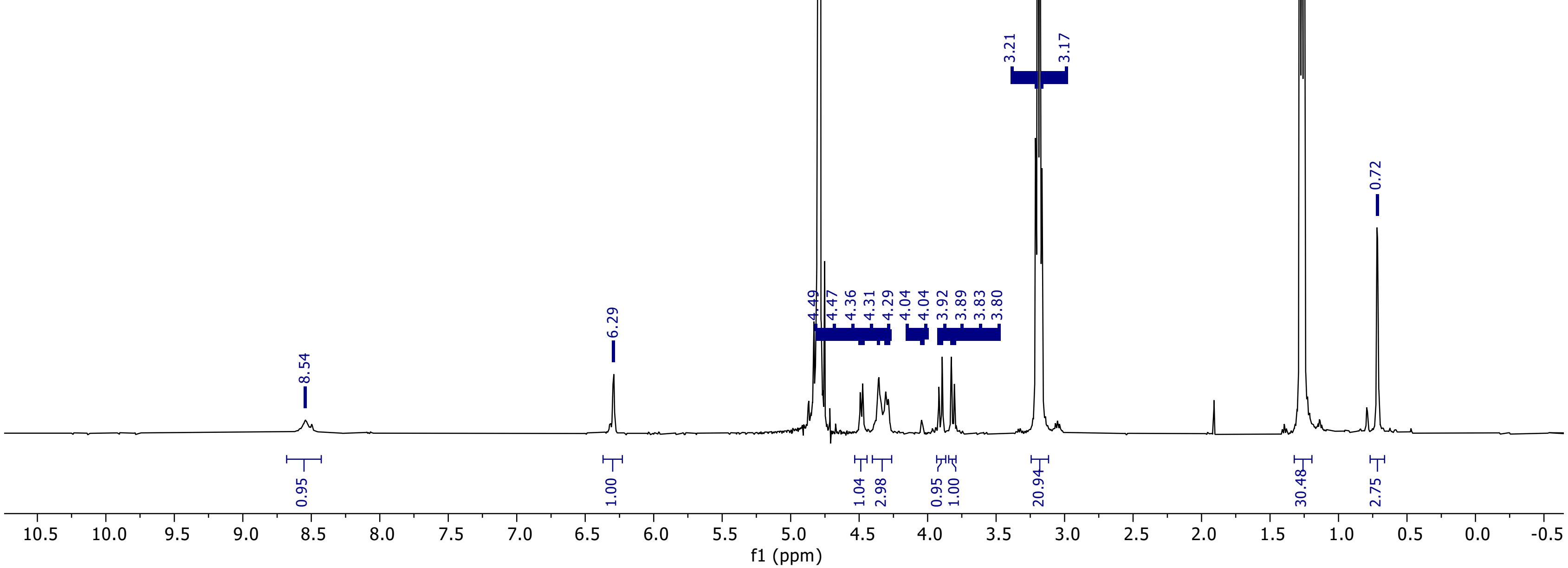

$^{13}C\{^{1}H\}$-NMR (176 MHz, D_2O)

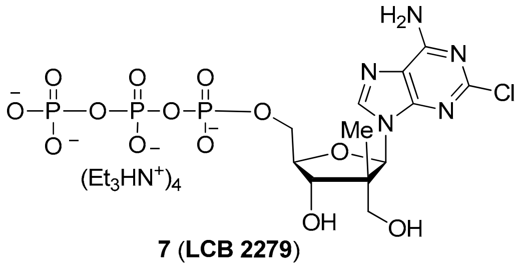

7 (**LCB 2279**)

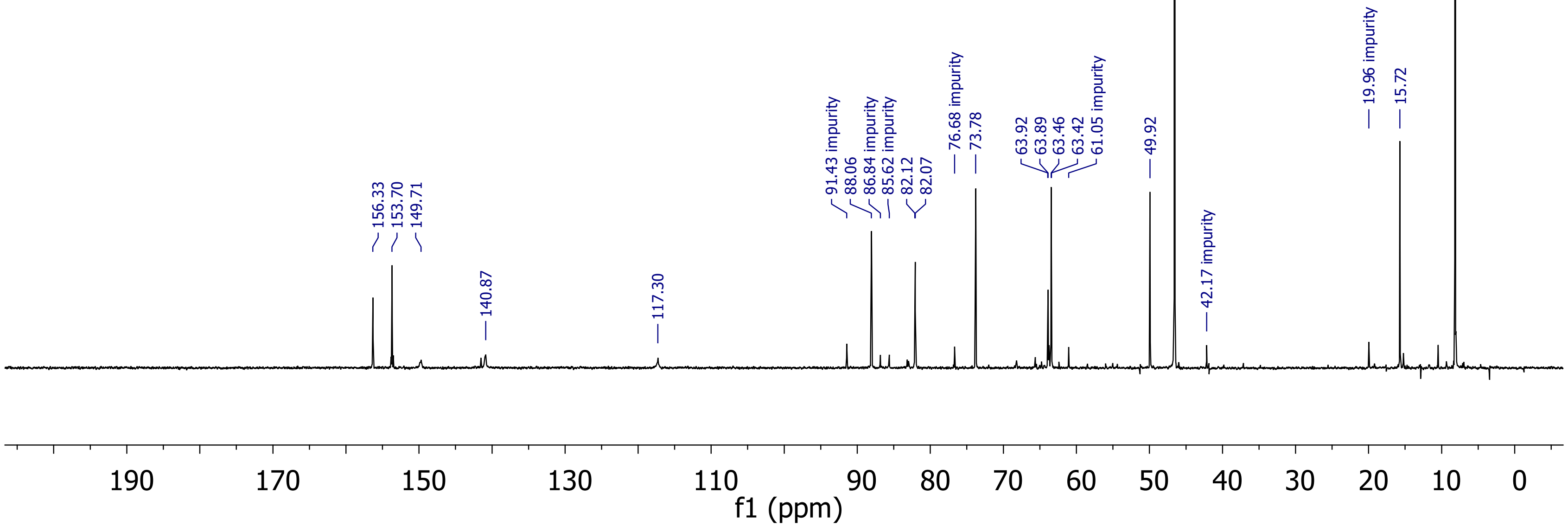

AM-ESP-854

^{31}P-NMR (162 MHz, D_2O)

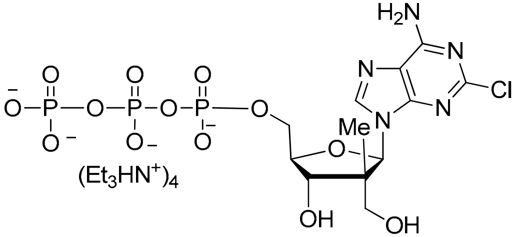

7 (**LCB 2279**)

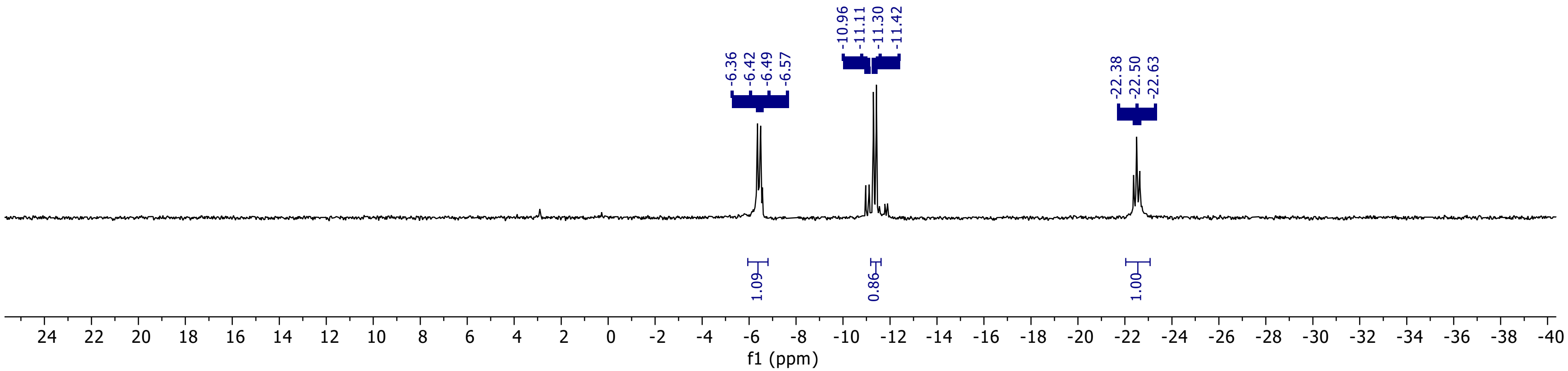

Supplement: Supplementary file 1 [file molecules-27-00564-s001.zip › molecules-1537446-supplementary.pdf]
